# Ligand Growing Experiments Suggested 4-amino and 4-ureido pyridazin-3(2*H*)-one as Novel Scaffold for FABP4 Inhibition

**DOI:** 10.3390/ph15111335

**Published:** 2022-10-28

**Authors:** Letizia Crocetti, Giuseppe Floresta, Chiara Zagni, Divya Merugu, Francesca Mazzacuva, Renan Rodrigues de Oliveira Silva, Claudia Vergelli, Maria Paola Giovannoni, Agostino Cilibrizzi

**Affiliations:** 1 Dipartimento NEUROFARBA—Pharmaceutical and Nutraceutical Section, via Ugo Schiff 6, Sesto Fiorentino, 50019 Florence, Italy; 2 Dipartimento di Scienze del Farmaco e della Salute, Università di Catania, Viale A. Doria 6, 95125 Catania, Italy; 3 Institute of Pharmaceutical Science, King’s College London, Stamford Street, London SE1 9NH, UK; 4 School of Health, Sport and Bioscience, University of East London, London E15 4LZ, UK; 5 Medicines Development, Centre for Therapeutic Innovation, University of Bath, Bath BA2 7AY, UK

**Keywords:** fatty acid binding protein, FABP4, FABP4is, FABP4 inhibitors, pyridazinone, computing assisted molecular design

## Abstract

Fatty acid binding protein (FABP4) inhibitors are of synthetic and therapeutic interest and ongoing clinical studies indicate that they may be a promise for the treatment of cancer, as well as other diseases. As part of a broader research effort to develop more effective FABP4 inhibitors, we sought to identify new structures through a two-step computing assisted molecular design based on the established scaffold of a co-crystallized ligand. Novel and potent FABP4 inhibitors have been developed using this approach and herein we report the synthesis, biological evaluation and molecular docking of the 4-amino and 4-ureido pyridazinone-based series.

## 1. Introduction

Fatty acids (FAs) are long carbon chain organic carboxylic acids responsible for different actions in the human organism [1,2]. Their chronic high concentration in circulation leads to various disorders [3,4], including atherosclerosis [5], diabetes [6] and obesity [7]. Considering that their chemical structure is characterized by high lipophilicity, FAs are insoluble in water, and their trafficking into the body requires specific carriers such as the fatty acid-binding proteins (FABPs). [8]. Since their discovery, FABPs have been classified into different families based on their localization in the human body, such as A-FABP (adipocyte), B-FABP (brain), E-FABP (epidermal), H-FABP (muscle and heart), I-FABP (intestinal), Il-FABP (ileal), L-FABP (liver), M-FABP (myelin), and T-FABP (testis). FABP4 (aP2 or A-FABP) is the subtype expressed in adipocytes [9], and the research into small molecule inhibitors for such protein initially started when it was reported that knockout animal models of FABP4 produced protective effects against the development of insulin resistance [10], as well as several pathological events linked to the metabolic syndrome and atherosclerosis [11,12,13]. Interestingly, pharmacological approaches with small molecules that inhibit the normal function of the protein are also valid in this regard, demonstrating similar results as the genetic procedures by mimicking the phenotype of FABP4-deficient mice [14]. This family of transporter proteins also has a role in cancer progression [15], and it was discovered that non-physiological expressions of FABPs are present in some of the most common cancers such as renal cell carcinoma, bladder and prostate, as well as other types of cancer cells [16,17,18]. It was recently discovered that FABP4 promotes the metastasis and invasion of colon cancer and that the treatment with a classical small molecule inhibitor (BMS309403) weakened the migration and invasion of colon cancer cells [19]. FABP4 leads also to abnormal metastasis patterns in ovarian cancer, and recent findings demonstrate that the protein is responsible for the disease’s aggressivity, contributing to poor prognosis in this tumor [20]. Moreover, the transporter has also been shown to play a role in accelerating glioblastoma cell growth [21]. All these recent findings related to cancer research proved that FABP4 targeting may represent an effective and promising therapeutic strategy against oncological conditions, in addition to the established effects on metabolic and cardiovascular diseases.

Recently, a variety of effective FABP4 inhibitors (FABP4i) have been developed, but unfortunately, none of them is currently in the clinical research phases [14,22]. Computer-aided drug design represents a promising and effective tool for the identification of molecular hits as FABP4i [23,24,25,26,27]. In line with our recent interest in the development of new antitumor compounds and the identification of novel bioactive heterocycles [28,29,30,31,32], herein we report the design, synthesis and in vitro characterization of 4-amino and 4-ureido pyridazinone-based series of FABP4i inspired by the scaffold hopping of an established ligand co-crystallized within the protein.

## 2. Results and Discussion 

### 2.1. Heterocyclic Small-Molecule Design 

**Figure 1:**
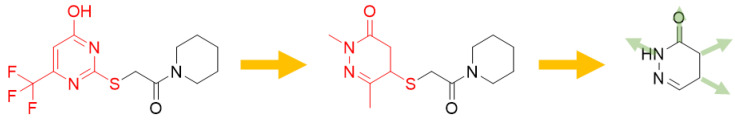
Schematic representation of the computer assisted design of the 4-amino and 4-ureido pyridazinones.

To generate a novel series of FABP4 inhibitors we have exploited a two-step computing assisted molecular design. As shown in Figure 1, in the first step of the drug-design process we focused on the search for bioisosteric-replacements/scaffold hopping of the pyrimidine scaffold of the co-crystallyzed ligand (2-[(2-oxo-2-piperidin-1 -ylethyl)sulfanyl]-6-(trifluoromethyl)pyrimidin-4-ol; pdbID: 1TOU). Our bioisosteric replacement analysis led to the selection of three nitrogen-containing heterocyclic frameworks, i.e., pyridazinones, pyridines and benzo[*d*]thiazole (see Supplementary Materials). Considering the synthetic accessibility of pyridazinone-based molecules and that pyridazinone was not investigated earlier as a scaffold to access FABP4 inhibitors, we envisaged to use this heterocycle to carry out automated ligand growing experiments inside the FABP4 cavity, as described in the Section 3, leading to 52 target molecules. The compounds were then synthesized and screened against FABP4 and the chemical structures are reported in Table 1 and Table 2. Both the scaffold hopping and the ligand growing experiments were conducted using Spark (https://www.cresset-group.com/products/spark/ accessed on 15 June 2022) [33].

### 2.2. Chemistry

**Scheme 1:**
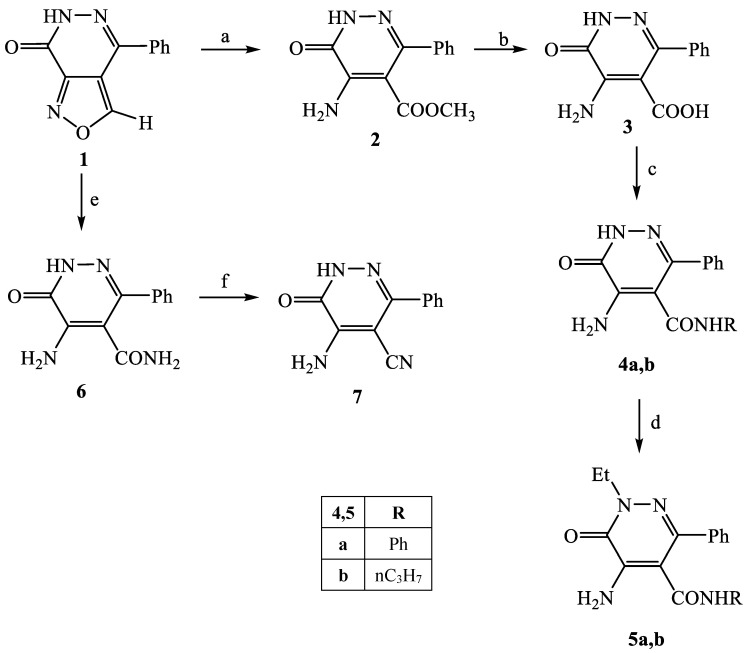
Reagents and conditions: (**a**) Et_3_N, CH_3_OH, 60 °C, 2 h; (**b**) 6N NaOH, EtOH, reflux, 30 min; (**c**) (i) SOCl_2_, Et_3_N, r.t., 30 min; (ii) R-NH_2_, anhydrous THF, r.t., 2 h; (**d**) CH_3_CH_2_Br, K_2_CO_3_, anhydrous DMF, reflux, 30–90 min; (**e**) 33% NH_4_OH, C_5_H_11_N, 60 °C, 90 min; (**f**) POCl_3_, 60 °C, 2h.

**Scheme 2:**
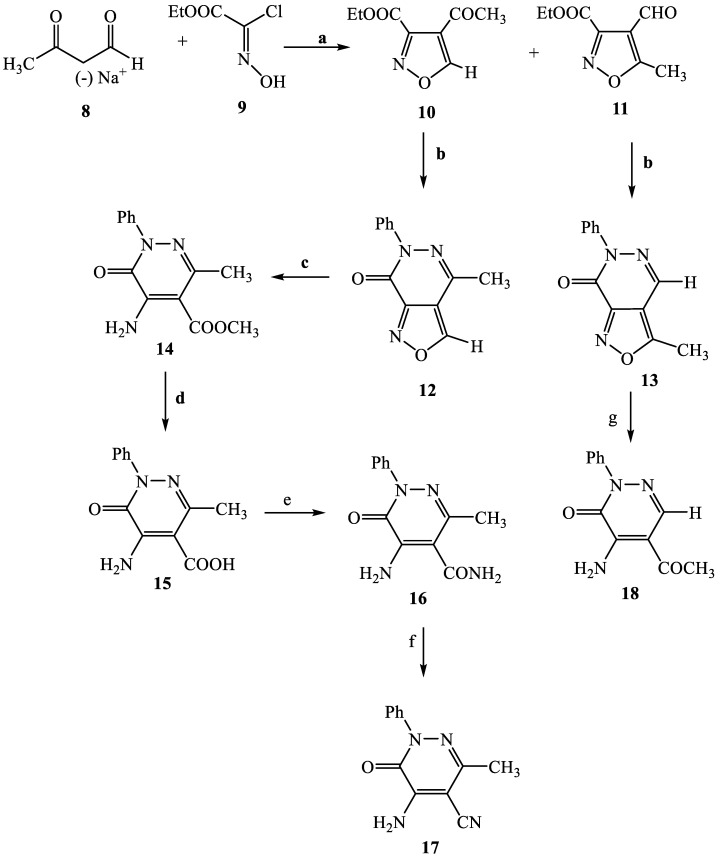
Reagents and conditions: (**a**) anhydrous EtOH, 0 °C, 1h; (**b**) phenylhydrazine, PPA, EtOH, 70 °C, 30 min; (**c**) CH_3_OH, Et_3_N, 60 °C, 2h; (**d**) NaOH, EtOH, reflux, 30 min; (**e**) (i) SOCl_2_, Et_3_N, reflux, 30 min.; (ii) 33% NH_4_OH, anhydrous THF, r.t., 15 min; (**f**) POCl_3_, 60 °C, 2h; (**g**) HCOONH_4_, 10% Pd/C, EtOH, reflux, 2 h.

**Scheme 3:**
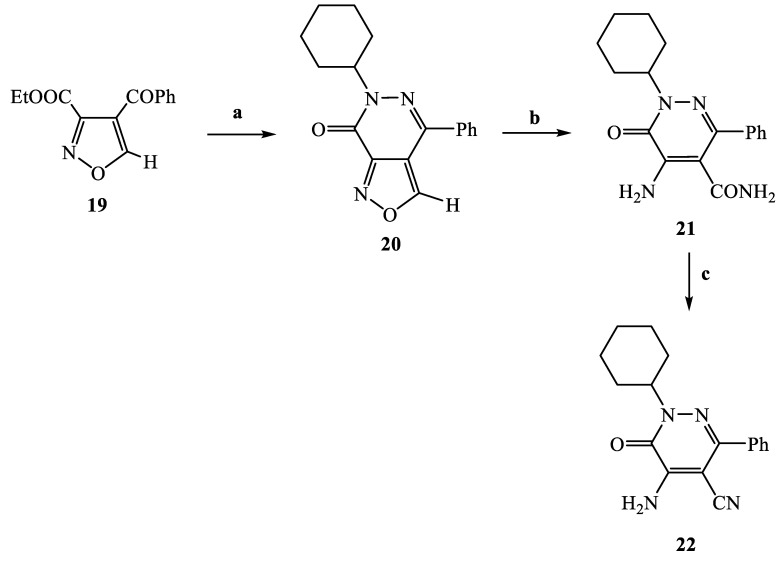
Reagents and conditions: (**a**) cyclohexylhydrazine, PPA, EtOH, 70 °C, 30 min; (**b**) 33% NH_3_, piperidine, 60 °C, 90 min; (**c**) POCl_3_, 60 °C, 2h.

**Scheme 4:**
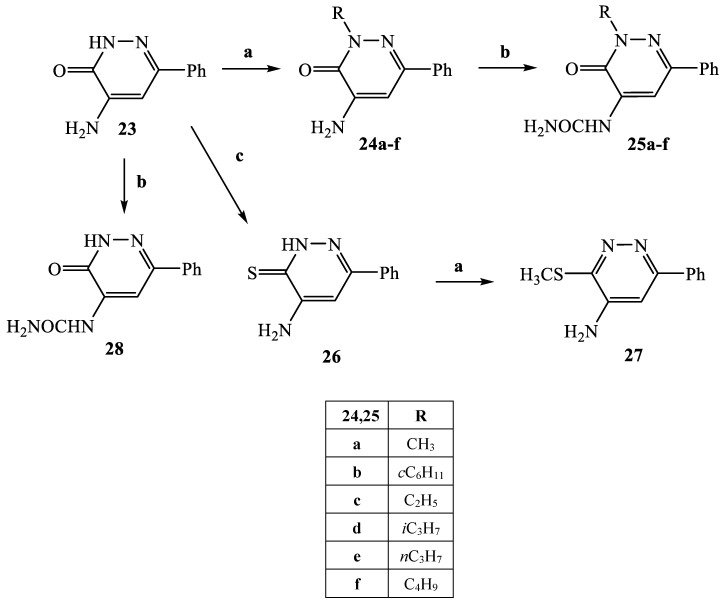
Reagents and conditions: (**a**) suitable R-Br, K_2_CO_3_, anhydrous DMF, reflux, 1–4 h; (**b**) (i) dry THF, CH_3_COONa, 0 °C then triphosgene, reflux, 2 h; (ii) NH_3_ 33%, 0 °C, 1 h; (**c**) Lawesson’s reagent, anhydrous toluene, reflux, 5 h.

**Scheme 5:**
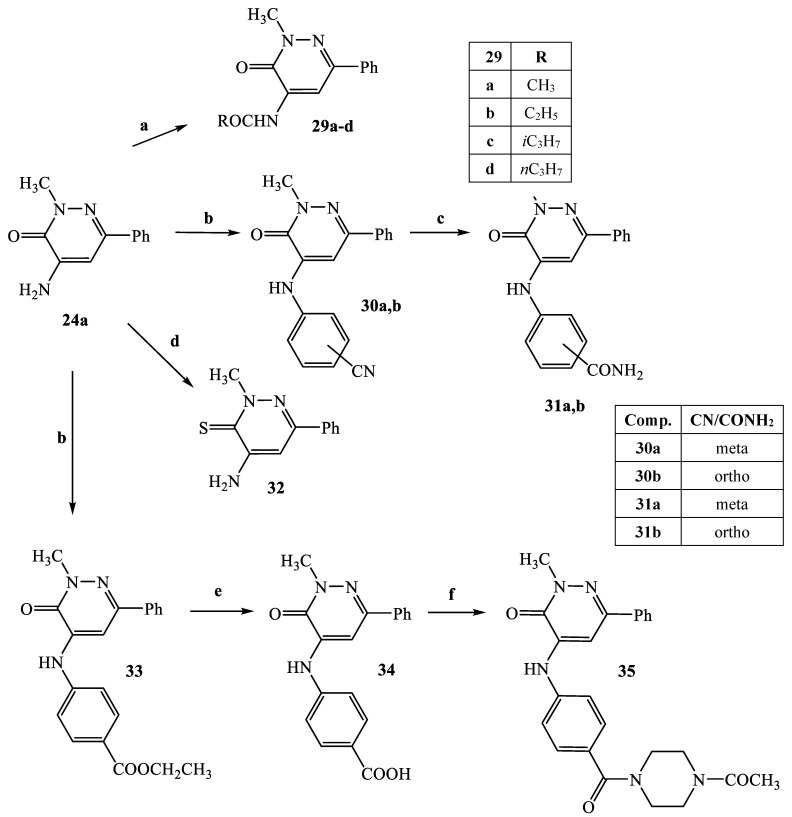
Reagents and conditions: (**a**) suitable (R-CO_2_)O, anhydrous C_6_H_5_N, closed tube, 140 °C, 5 h; (**b**) 2/3-cyanophenylboronic acid (for **30a,b**) or 4-ethoxycarbonylphenylboronic acid (for **33**), Cu(Ac)_2_, Et_3_N, dry CH_2_Cl_2_, r.t., 12 h; (**c**) H_2_SO_4_ 80%, 80 °C, 4 h; (**d**) Lawesson’s reagent, anhydrous toluene, reflux, 10 h; (**e**) NaOH 6N, EtOH 96%, reflux, 1 h; (**f**) (i) SOCl_2_, Et_3_N (catalytic), reflux, 1 h; (ii) anhydrous THF, 1-acetylpiperazine, 0 °C then r.t., 1 h.

**Scheme 6:**
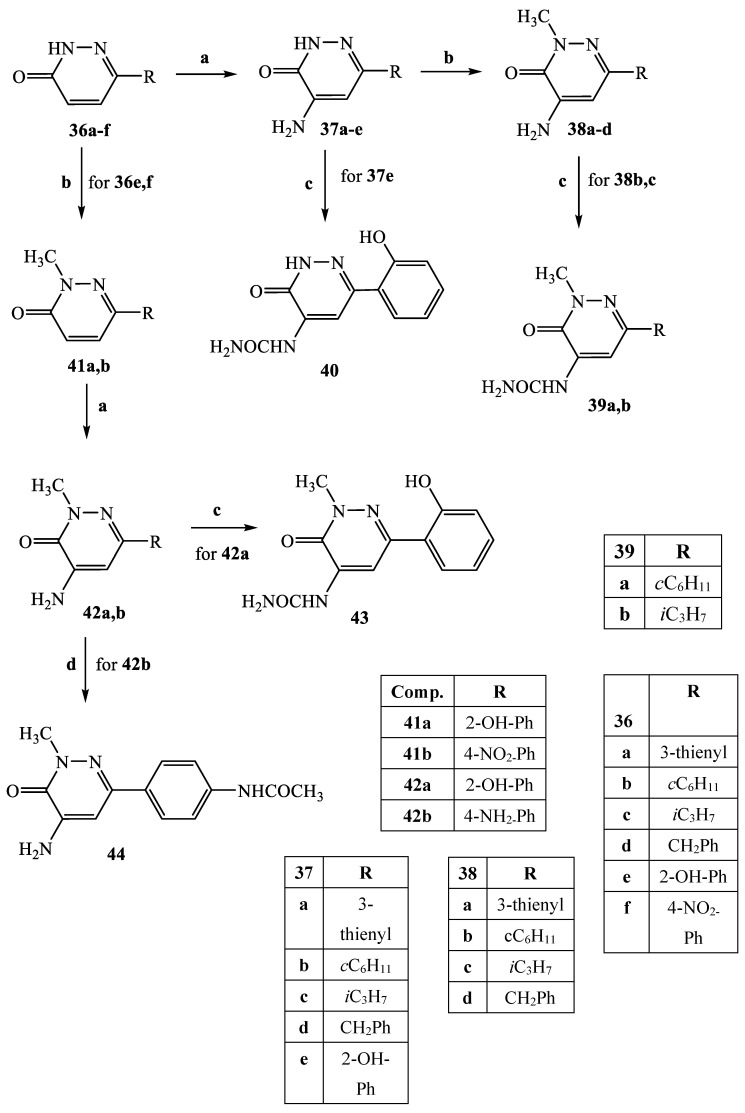
Reagents and conditions: (**a**) NH_2_NH_4_·H_2_O, sealed/pressure vessel, 180 °C, 12 h; (**b**) CH_3_I, K_2_CO_3_, anhydrous DMF, 80 °C, 2–4 h; (**c**) (i) anhydrous THF, CH_3_COONa, 0 °C then triphosgene, reflux, 2 h; (ii) NH_4_OH 33%, 0 °C, 1 h; (**d**) ClCOCH_3_, anhydrous THF, 0 °C, then r.t., 20 min.

**Scheme 7:**
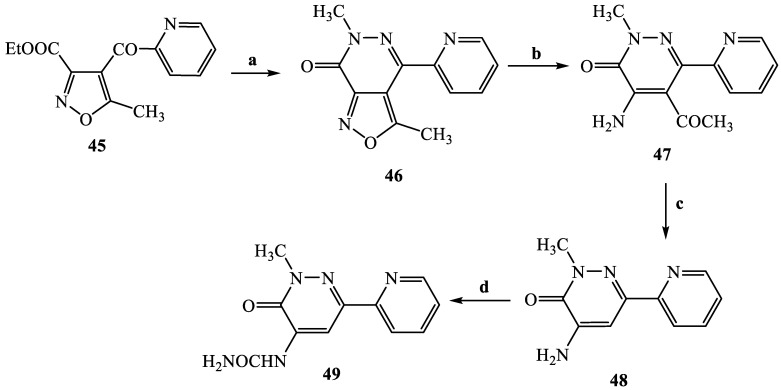
Reagents and conditions: (**a**) CH_3_(NH)NH_2_, EtOH 96%, r.t., 2 h; (**b**) HCOONH_4_, Pd/C, EtOH 96%, reflux, 2h; (**c**) HBr 48%, sealed/pressure vessel, 130 °C, 3 h; (**d**) (i) anhydrous THF, CH_3_COONa, 0 °C then triphosgene, reflux, 2 h; (ii) 33% NH_4_OH, 0 °C, 1 h.

**Scheme 8:**
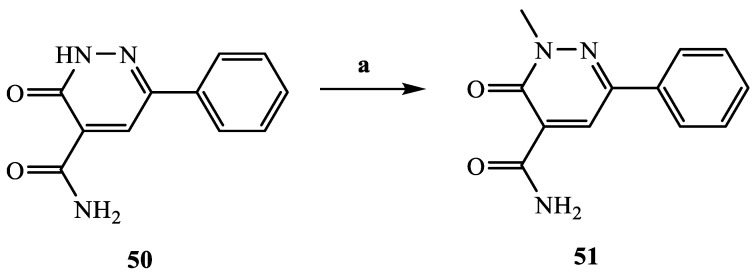
Reagents and conditions: (**a**) CH_3_I, K_2_CO_3_, anhydrous DMF, 80 °C, 2 h.

**Scheme 9:**
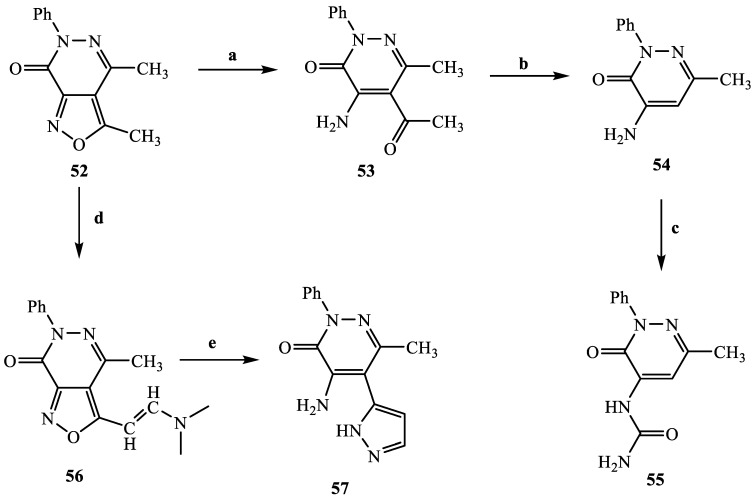
Reagents and conditions: (**a**) HCOONH_4,_ Pd/C 10%, EtOH 96%, reflux, 2 h; (**b**) HBr 48%, sealed/pressure vessel, 130 °C, 3 h; (**c**) (i) anhydrous THF, CH_3_COONa, 0 °C then triphosgene, reflux, 2 h; (ii) NH_4_OH 33%, 0 °C, 1 h; (**d**) DMF-DMA, 90 °C, 1 h; (**e**) NH_2_NH_4_·H_2_O, anhydrous EtOH, 70 °C, 10 h.

The synthetic procedures carried out to obtain the target compounds containing the pyridazinone scaffold are reported in Scheme 1, Scheme 2, Scheme 3, Scheme 4, Scheme 5, Scheme 6, Scheme 7, Scheme 8 and Scheme 9. The structures were confirmed on the basis of analytical and spectral data. Scheme 1 shows the synthetic pathway affording the final compounds **4a,b**, **5a,b**, **6** and **7**. Intermediate **2** [34] was obtained starting from isoxazole-pyridazinone **1**, synthesized by adopting previously reported protocols [29,30,31,32] and using methanol and triethylamine for opening the isoxazole *nucleus*. The subsequent hydrolysis (acid **3** [35]) and acylation with thionyl chloride, triethylamine and appropriate amine led to final compounds **4a,b**. Products **5a,b** were obtained from alkylation reaction of **4a,b** with ethyl bromide in standard conditions. The opening of isoxazole *core* of the starting material **1** with 33% NH_4_OH afforded to amide **6** [28] which, by subsequent dehydration with POCl_3_, led to compound **7**. The synthesis of final compounds **16**–**18** is reported in Scheme 2. The reaction between sodium salt of diketone **8** with the commercially available ethyl chloro(hydroximino)acetate **9** in ethanol led to a mixture of isomers **10** and **11 [36]** that were cyclized to isoxazole-pyridazinone **12** and **13** using phenylhydrazine and PPA. After chromatographic separation, the latter were subjected to a series of reactions to obtain the compounds **16**–**18**. The treatment of intermediate **13** with ammonium formate and Pd/C provided compound **18**, while the treatment of **12** with methanol and triethylamine led to pyridazinone **14**. Intermediate **14** was first hydrolyzed to acid (**15**), then converted to amide (**16**) and finally treated with POCl_3_ to obtain the cyano derivative **17**. The final compounds **21** and **22** were obtained through a procedure similar to that shown in Scheme 1 for amide derivative **6** and cyano derivative **7**, using intermediate **20** as the starting material, which was obtained by reaction of cyclohexyl hydrazine and PPA with isoxazole **19 [34]** (see Scheme 3). Scheme 4 reports the synthesis of the pyridazinone-based derivatives of type **24** and **25** (unsubstituted at position 5), compound **28** and the thio-derivative **27**. Intermediate **23** [37] was reacted with the appropriate brominated alkylating agent in presence of potassium carbonate and dry DMF to afford **24a**–**f** derivatives (**24a**, [38]; **24c**, [34]). The formation of urea derivatives of type **25** was carried out using sodium acetate and triphosgene in dry THF at reflux, and then treated with ammonia. The urea **28** was directly obtained from intermediate **23** using the same conditions used for compound type **25**. The transformation of the carbonyl (C=O) in thiocarbonyl group (C=S) was carried out using the Lawesson’s reagent in toluene (**26**) and the subsequently alkylation with methyl iodide in standard condition led to the thio derivative **27**. In Scheme 5 and Scheme 6 are reported the synthetic procedures of other un-substituted pyridazinones at position 5, but bearing different groups/functions at position 4 and 6. In particular, Scheme 5 depicts the synthetic pathways for compounds with a phenyl ring at position 6 and a methyl group at N-2, while different substituents are introduced at position 4. Starting from compound **24a [38]** (Scheme 4), the amino group at position 4 was acylated using the suitable anhydride in pyridine in a sealed/pressure vessel to obtain the final compounds **29a**–**d**. Moreover, the same amino group was also subjected to a coupling reaction using the appropriate R-phenylboronic acid in presence of copper (II) acetate and triethylamine to furnish the derivatives **30a,b** and **33**. The substituent R on the phenyl at position 4 was further elaborated. The *m*/*o*-CN group of compounds **30a,b** was converted into *m*/*o*-CONH_2_ (compounds **31a,b**, respectively) with 80% sulfuric acid under reflux. The 4-carbethoxy function in product **33** was firstly hydrolyzed to acid **34,** converted into the corresponding acid chloride with thionyl chloride and then acylated with 1-acetylpiperazine (compound **35**). Lastly, the carbonyl group of intermediate **24a** was converted in thiocarbonyl (**32**) using the same procedure discussed in Scheme 4. In Scheme 6 are depicted pyridazinone-based derivatives with a methyl group or a hydrogen at N-2, an amino group or urea functionality at position 4, but bearing different groups e/o functions (e.g., R-phenyl, alkyl, cycloalkyl) at position 6. Starting from commercially available intermediates **36a**–**f**, the introduction of an amino group at position 4 with hydrazine hydrate at high temperature led to compounds **37a**–**e** (**37e**, [38]) and the subsequent alkylation with methyl iodide provided products **38a**–**d**. The derivatives **39a,b** and **40** were obtained from reaction with triphosgene and ammonia in the same conditions reported in Scheme 4, starting from **38b,c** and **37e**, respectively. The direct alkylation of the intermediates **36e** and **36f** afforded the corresponding *N*-methyl derivatives **41a,b** (**41a,** [39]), which were subsequently converted into compounds **42a,b** through the same reaction used to obtain **37a**–**e**. In particular, the reaction conditions used to introduce an amino group at position 4 led also to the reduction in the nitro group in compound **42b**. The latter was subjected to acylation reaction with acetyl chloride to obtain product **44**. Instead, intermediate **42a** was subjected to triphosgene treatment to obtain the urea derivative **43**. Scheme 7 reports the synthesis of final compounds **48** and **49**. Intermediate **47** was obtained starting from isoxazole **45,** previously synthesized by us [34] by cyclization reaction with methyl hydrazine (**46**) and subsequent opening of the isoxazole ring with ammonium formate and palladium on carbon. The deacetylation (on **47**) with 48% bromic acid at high temperature led to compound **48**, which was subsequently treated with triphosgene and ammonia to obtain the urea derivative **49**. Compound **51** was obtained through alkylation reaction using standard conditions [40], but starting from product **50** [41] (Scheme 8). Lastly, the final compound **55** (Scheme 9) was obtained starting from intermediate **52** [42], through the same reactions of isoxazole nucleus opening (**53 [42]**), deacetylation (**54 [43]**) and formation of the urea function. In Scheme 9 is also illustrated the treatment of **52** with dimethylformamide dimethyl acetal to generate intermediate **56**, which was subsequently converted into compound **57** using hydrazine hydrate. 

Based on the analytical and spectral data (proton and carbon NMR) and mass spectrometry (MS), all the new compounds confirmed the predicted chemical structures, as well as satisfactory results in terms of formulation and purity (See Section 3; in Supporting Information are reported representative examples of analytical characterization data of the compounds processed to FABP4 inhibition assay in vitro). Reversed phase liquid chromatography was used to perform a qualitative analysis of the dataset’s purity. The formation of the products was monitored by UV absorbance at wavelengths of 281 nm and 254 nm. The retention times range was from 6 to 17 min (See Section 3 and Supporting Information). The overall feature of mass spectra (LC-MS) of this series of pyridazinone-derivatives is the presence of a predominant peak corresponding to the molecular ion [M + H]^+^ (See Section 3 and Supporting Information).

### 2.3. FABP4 Inhibition Evaluation

**Figure 2:**
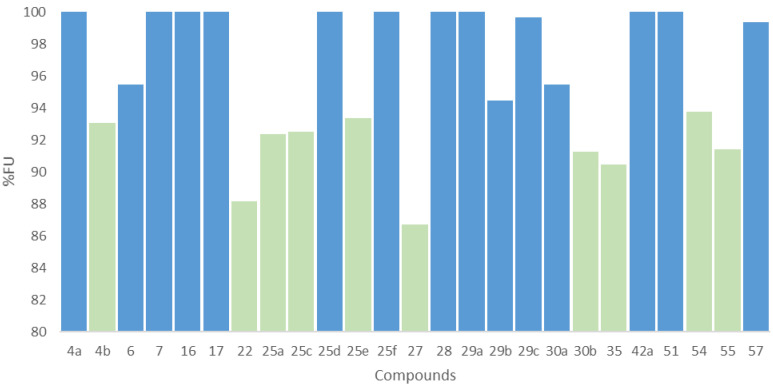
Single point displacement experiment for selected compounds.

FABP4 inhibitory activity was assessed by measuring the decrease in fluorescent signal of a detection reagent (DR) when displaced by a strong FABP4 ligand. Specifically, the DR exhibits an increased fluorescence intensity when bound to FABP4. Therefore, any effective ligand of the protein, which binds to the same binding pocket and can displace the DR, determines a reduction in the fluorescence read-out. The new molecular series was screened in a two-step procedure. Firstly, a single concentration of 5 µM was used to gain an estimation of the overall inhibitory effect of all the molecules. Subsequently, only the compounds that were able to reduce the fluorescence reading of at least 95% were further evaluated by measuring the IC_50_ values (µM), which were lastly compared with the activity of the arachidonic acid (i.e., FABP4 established ligand). The single point displacement results are reported in Figure 2. Based on the data of the first screening, 10 molecules were selected as most effective compounds—i.e., able to reduce the fluorescence of the DR to at least 95%, for which the IC_50_ (µM) was calculated. Arachidonic acid was used as a positive control, resulting with an IC_50_ of 3.42 µM. The IC_50_ values of our set of compounds are reported in Table 3. Compound **25a** demonstrated a potent inhibitory activity, with an IC_50_ value (i.e., 2.97 µM) lower than the reference arachidonic acid.

### 2.4. Molecular Modelling Studies

**Figure 3:**
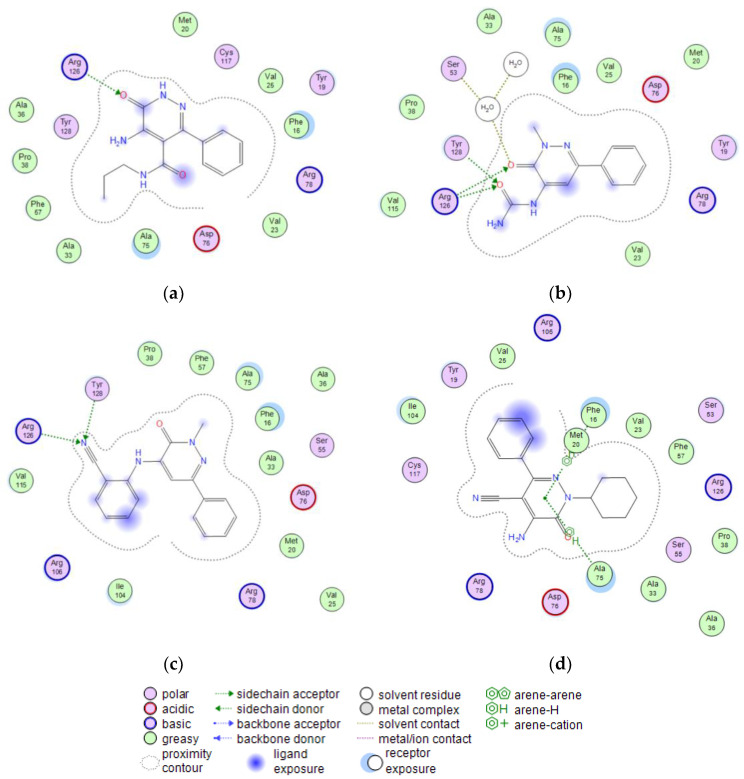
(**a**) 2D interaction between **4b** and FABP4. (**b**) 2D interaction between **25a** and FABP4. (**c**) 2D interaction between **30b** and FABP4. (**d**) 2D interaction between **22** and FABP4.

**Figure 4:**
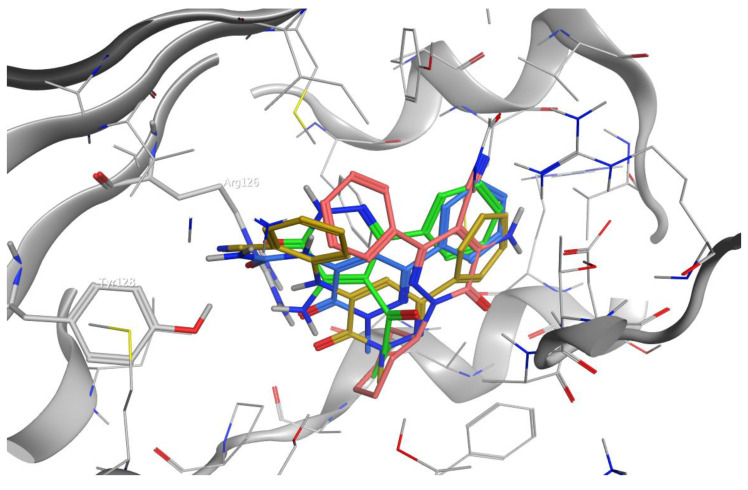
Docked poses inside FABP4 of molecules **4b** (green), **25a** (blue), **30b** (dark yellow) and **22** (light red).

Since the first apo-FABP crystal structure was published in 1992, many other holo-FABP structures with a variety of ligands have been solved. The hydrophobic pocket side chains engage a hydrogen bond to the carboxylate of FAs toward several amino acids. Moreover, a network of water molecules may be involved in mediating these interactions. The docking experiments of the molecular series compounds were conducted on the most active compounds **4b**, **25a**, **30b**, and **22**. Figure 3 shows the 2D binding interactions for the molecules, while Figure 4 displays the predicted poses inside the binding pocket of FABP4. All the compounds are able to engage several interactions with relevant residues in the binding pocket, such as R126 and Y128, as well as R106. R126 can interact with both the carbonyls of the most potent compound **25a**, that also interacts directly with Y128 and, through the network of water molecules, with S53. The **4b** is well allocated inside the binding pocket and is engaging a strong H-bond interaction with R126. Differently, compound **22** is not suitably allocated inside the pocket to generate appropriate binding with R126 and Y128 and most of the stabilizing interactions are due to pi–pi stacking with A75, F16 and M20. Lastly, the -CN group of **30b** results responsible of the stabilizing interaction with R126 and Y128, that are likely to account for the lower activity of the compound, as determined by the lower binding interaction for this group with the residues.

## 3. Experimental Section

### 3.1. General Remarks

All the chemical reagents were purchased from Merk and Sigma Aldrich of reagent grade and were used without any further purification. Extracts were dried over Na_2_SO_4_ and the solvents were removed under reduced pressure. All reactions were monitored by thin-layer chromatography (TLC) using commercial plates (Merck) pre-coated with silica gel 60 F-254. Visualization was performed by UV fluorescence (λmax = 254 nm) or by staining with iodine or potassium permanganate. Chromatographic separations were performed on silica gel columns by gravity (Kieselgel 40, 0.063–0.200 mm; Merck) or flash chromatography (Kieselgel 40, 0.040–0.063 mm; Merck). Yields refer to chromatographically and spectroscopically pure compounds, unless otherwise stated. When reactions were performed in anhydrous conditions, the mixtures were maintained under nitrogen atmosphere. Compounds were named following IUPAC rules as applied by Beilstein-Institut AutoNom 2000 (4.01.305) or CA Index Name. All melting points were determined on a microscope hot stage Büchi apparatus and are uncorrected. ^1^H-NMR and ^13^C-NMR spectra were obtained on a Bruker AVANCE 400 spectrometer at 400 MHz and 100 MHz, respectively, using 5 mm i.d. glass tubes. Chemical shifts (δ) values are expressed as parts per million (ppm) using DMSO (d_6_) (2.50 for proton and 39.52 for carbon), methanol (d_4_) (3.31 for proton and 49.00 for carbon) or CDCl_3_ (7.26 for proton and 77.16 for carbon) as solvents. The coupling constants (J) are reported in Hz. The following splitting patterns are identified: s, singlet; d, doublet; t, triplet; m, multiplet; or any combination of these e.g., dd, dt, etc. Analytical reversed-phase high performance liquid chromatography (reversed-phase HPLC) was conducted out on HP 1050 instrument (Agilent Technologies, Waldbronn, Germany) to ascertain the chromatographic purity of compounds. The system includes a quaternary pump, an autosampler, and a Kontron DEG 104 degasser (Kontron, Tokyo, Japan). A C_18_ column, Zorbax,80 Å, 3.5 μm, 2.1 × 100 mm was used with a total run time of 30 min. The mobile phase is composed of 0.1% Trifluoro acetic acid (TFA) in Milli-Q H_2_O and Acetonitrile (can) at a flow rate of 0.3 mL/min with an injection volume of 10–30 μL [44]. The compounds were detected at 281 nm and 254 nm UV wavelengths. The values of the retention times (t_R_) are given in minutes. Mass spectrometry (LC-MS) experiments were performed on all the samples. The stock solutions (1 mg/mL in MeOH) where diluted with 0.1% HCOOH in MeOH/H_2_O (50:50) to a final concentration of 50 µg/mL prior to analysis. The instrument used consisted of a Thermo Accela LC system interfaced to a Thermo TSQ Access triple quadrupole mass spectrometer with a HESI source. The data were processed with Xcalibur software (version 2.0). An amount of 10 µL of sample was analyzed in flow injection, with a flow rate of 0.2 mL/min of mobile phase 0.1% HCOOC in MeOH/H_2_O (50:50). Parameters used for the analysis in positive ion mode were: spray voltage 3500 V; vaporizer temperature 300 °C; sheath gas pressure 50 au; capillary temperature 350 °C; capillary offset 35.

### 3.2. Chemistry

#### 3.2.1. General Procedure for Compounds **4a,b**

A mixture of **3** (0.35 mmol) [35], a catalytic amount of Et_3_N (0.1 mL) and SOCl_2_ (9.35 mmol) was stirred at room temperature for 30 min. Then the excess of SOCl_2_ was removed in vacuo and the residue oil was dissolved in cold anhydrous THF (1 mL). To this suspension, the appropriate amine (0.75 mmol) was added and the mixture was stirred at room temperature for 2 h. After cooling, cold water was added (2–5 mL) and the suspension was extracted with CH_2_Cl_2_ (3 × 15 mL); the solvent was evaporated under vacuum to afford the desired final compounds, which were purified by flash column chromatography using cyclohexane/ethyl acetate 1:2 as eluent (4a), or by crystallization from ethanol (4b).

##### 5-Amino-6-oxo-3-phenyl-1,6-dihydropyridazine-4-carboxylic acid phenylamide (**4a**)

Yield = 40%; mp = 228–229 °C (EtOH). Light brown solid, ^1^H NMR (400 MHz, DMSO-d_6_) δ 6.57 (s, 2H, NH_2_), 7.02 (t, 1H, J = 7.4 Hz, ArCONH), 7.23 (t, 2H, J = 7.8 Hz, Ar), 7.32 (d, 2H, J = 7.3 Hz, Ar), 7.39 (d, 2H, J = 8.0 Hz, Ar), 7.47–7.49 (m, 2H, Ar), 10.04 (s, 1H, CONH_2_), 12.88 (s, 1H, ArNH). ^13^C NMR (100 MHz, DMSO-d_6_) δ163.75, 156.35, 155.43, 145.54, 141.87, 138.61, 128.43, 128.24, 127.93, 123.86, 119.95, 109.88. MS-ESI for C_17_H_14_N_4_O_2_ (Calcd, 306.11), [M + H]^+^ at *m/z* 306.96, t_R_ = 11.825. Anal. Calcd for C_17_H_14_N_4_O_2_: C, 66.66; H, 4.61; N, 18.29. Found C, 66.92; H, 4.63; N, 18.36.

##### 5-Amino-6-oxo-3-phenyl-1,6-dihydropyridazine-4-carboxylic acid propylamide (**4b**)

Yield = 35%; mp = 228–230 °C (EtOH). Yellow coloured solid, ^1^H NMR (400 MHz, DMSO-d_6_) δ 0.57–0.61 (m, 2H, CH_3_), 1.18 (dp, 2H, J = 14.2, 7.2 Hz, CH_2_), 2.94 (q, 2H, J = 6.8 Hz, NHCH_2_), 6.31 (s, 2H, NH_2_), 7.36 (dt, 2H, J = 4.5, 1.6 Hz, Ar), 7.44 (dq, 2H, J = 6.4, 1.7 Hz, Ar), 7.98 (t, 1H, J = 5.8 Hz, Ar), 12.79 (s, 1H, ArNH). ^13^C NMR (100 MHz, DMSO-d_6_) δ 164.94, 156.30, 145.52, 141.61, 137.07, 128.17, 127.90, 127.80, 110.26, 40.55, 21.51, 11.24. MS-ESI for C_14_H_16_N_4_O_2_ (Calcd, 272.13), [M + H]^+^ at *m/z* 273.02, t_R_ = 9.970. Anal. Calcd for C_14_H_16_N_4_O_2_: C, 61.75; H, 5.92; N, 20.58. Found C, 61.99; H, 5.94; N, 20.66.

#### 3.2.2. General Procedure for Compounds **5a,b**

A mixture of **4a,b** (0.43 mmol), K_2_CO_3_ (0.86 mmol) and 0.50 mmol of ethyl bromide in anhydrous DMF (2 mL) was refluxed for 30–90 min. After cooling, the mixture was diluted with cold water (15 mL) and compound **5a** was recovered by filtration under vacuum. For compound **5b** the suspension was extracted with CH_2_Cl_2_ (3 × 15 mL) and the solvent was evaporated in vacuo. The crude products were purified by crystallization from ethanol.

##### 5-Amino-1-ethyl-6-oxo-3-phenyl-1,6-dihydropyridazine-4-carboxylic acid phenylamide (**5a**)

Yield = 90%; mp = 172–173 °C (EtOH). ^1^H NMR (400 MHz, CDCl_3_) δ 1.42 (t, 3H, CH_3_CH_2_, J = 7.2 Hz), 4.26 (q, 2H, CH_3_CH_2_, J = 7.2 Hz), 6.70 (exch br s, 1H, CONH), 6.93 (d, 2H, Ar, J = 8.0 Hz), 7.04 (t, 1H, Ar, J = 8.0 Hz), 7.20 (t, 2H, Ar, J = 8.0 Hz), 7.49–7.54 (m, 3H, Ar), 7.55–7.60 (m, 2H, Ar). Anal. Calcd for C_19_H_18_N_4_O_2_: C, 68.25; H, 5.43; N, 16.76. Found C, 68.41; H, 5.44; N, 16.72. 

##### 5-Amino-1-ethyl-6-oxo-3-phenyl-1,6-dihydro-pyridazine-4-carboxylic acid propylamide (**5b**)

Yield = 80%; mp = 141–143 °C (EtOH). ^1^H NMR (400 MHz, CDCl_3_) δ 0.64 (t, 3H, NH-CH_2_CH_2_CH_3_, J = 7.2 Hz), 1.15 (sex, 2H, NH-CH_3_CH_2_CH_2_, J = 7.6 Hz), 1.43 (t, 3H, N-CH_2_CH_3_, J = 7.2 Hz), 3.05 (q, 2H, NH-CH_2_CH_2_CH_3_, J = 7.2 Hz), 4.25 (q, 2H, N-CH_2_CH_3_, J = 7.2 Hz), 5.02 (exch br s, 1H, CONHCH_2_), 6.95 (exch br s, 2H, NH_2_), 7.45–7.51 (m, 5H, Ar). Anal. Calcd for C_16_H_20_N_4_O_2_: C, 63.98; H, 6.71; N, 18.65. Found C, 63.83; H, 6.70; N, 18.70.

#### 3.2.3. 5-Amino-6-oxo-3-phenyl-1,6-dihydropyridazine-4-carboxylic acid amide (**6**)

A mixture of isoxazolopyridazinone **1** (0.94 mmol) [29], 2 mL of 33% NH_3_ and a catalytic amount of piperidine was stirred at 60 °C for 90 min in a sealed/pressure vessel. After cooling the precipitate was recovered by suction and recrystallized with diethyl ether. Yield = 46%; mp > 300 °C (Et_2_O). Light brown solid, ^1^H NMR (400 MHz, DMSO-d_6_) δ 6.38 (s, 2H, NH_2_), 7.38 (dd, 3H, Ar, J = 5.0, 2.1 Hz), 7.47–7.49 (m, 2H, Ar), 12.79 (s, 1H, ArNH). ^13^C NMR (100 MHz, DMSO-d_6_) δ 167.25, 156.28, 145.36, 141.66, 137.17, 128.17, 127.97, 127.83, 109.75. MS-ESI for C_11_H_10_N_4_O_2_ (Calcd, 230.08), [M + H]^+^ at *m/z* 230.95, t_R_ = 6.091. Anal. Calcd for C_11_H_10_N_4_O_2_: C, 57.39; H, 4.38; N, 24.34. Found C, 57.16; H, 4.36; N, 24.24. 

#### 3.2.4. 5-Amino-6-oxo-3-phenyl-1,6-dihydropyridazine-4-carbonitrile (**7**)

A suspension of **6** (0.40 mmol) in POCl_3_ (8 mmol) was stirred at 60 °C for 1–2 h. After cooling, the reaction mixture was treated with cold water (15 mL) and the suspension was extracted with CH_2_Cl_2_ (3 × 15 mL). The organic solvent was evaporated to afford the desired final compound which was purified by crystallized from diethyl ether. Yield = 48%; mp = 287–289 °C (Et_2_O). Yellow coloured solid, ^1^H NMR (400 MHz, DMSO-d_6_) δ 6.78 (s, 1H, NH_2_), 7.48 (tt, 3H, J = 3.9, 2.4 Hz, Ar), 7.57–7.61 (m, 2H, Ar), 12.98 (s, 1H, ArNH). ^13^C NMR (100 MHz, DMSO-d_6_) δ154.47, 154.06, 149.95, 145.72, 135.21, 129.28, 128.29, 128.13, 115.68, 113.42. MS-ESI for C_11_H_8_N_4_O (Calcd, 212.07), [M + H]^+^ at *m/z* 212.89, t_R_ = 10.234. Anal. Calcd for C_11_H_8_N_4_O: C, 62.26; H, 3.80; N, 26.40. Found C, 62.01; H, 3.78; N, 26.29. 

#### 3.2.5. 4-Acetyl-isoxazole-3-carboxylic acid ethyl ester (**10**)

To a cooled (−5 °C) and stirred suspension of **8** (9.9 mmol) in anhydrous ethanol, a solution of ethyl chloro(hydroximino)acetate **9** (6.6 mmol) in the same solvent (11 mL) was added dropwise. The solvent was evaporated in vacuo, cold water was added (10 mL) and the suspension was extracted with CH_2_Cl_2_ (3 × 15 mL). A mixture of isoxazoles **10** and **11** [36] was obtained and they were separated by flash column chromatography using cyclohexane/ethyl acetate 2:1 as eluent. Yield = 15%; oil. ^1^H NMR (400 MHz, CDCl_3_) δ 1.45 (t, 3H, CH_2_CH_3_, J = 7.2 Hz), 2.55 (s, 3H, COCH_3_), 4.51 (q, 2H, CH_2_CH_3_, J = 7.2 Hz), 8.95 (s, 1H, Ar). Anal. Calcd for C_8_H_9_NO_4_: C, 52.46; H, 4.95; N, 7.65. Found C, 52.33; H, 4.94; N, 7.67.

#### 3.2.6. General Procedure for Compounds **12** and **13**

To a cooled and stirred mixture of isoxazoles **10** or **11** (6.56 mmol) and 2.5 g of PPA (25 mmol) in 2 mL of anhydrous EtOH, 7.87 mmol of phenylhydrazine were added. The reaction was carried out at 70 °C for 30 min. After cooling the solvent was evaporated under vacuum, cold water was added (10 mL) and the suspension was extracted with CH_2_Cl_2_ (3 × 15 mL). Evaporation of the solvent afforded the desired compounds. 

##### 4-Methyl-6-phenyl-6H-isoxazolo [3,4-d]pyridazin-7-one (**12**)

Yield = 90%; mp = 200–201 °C (Cyclohexane). ^1^H NMR (400 MHz, CDCl_3_) δ 2.54 (s, 3H, CH_3_), 7.38 (t, 1H, Ar, J = 7.6 Hz), 7.45–7.50 (m, 2H, Ar), 7.55–7.60 (m, 2H, Ar), 9.22 (s, 1H, C=CH). Anal. Calcd for C_12_H_9_N_3_O_2_: C, 63.43; H, 3.99; N, 18.49. Found C, 63.58; H, 4.00; N, 18.44.

##### 3-Methyl-6-phenyl-6H-isoxazolo [3,4-d]pyridazin-7-one (**13**)

Yield = 80%; mp = 188–190 °C (Cyclohexane). ^1^H NMR (400 MHz, CDCl_3_) δ 2.88 (s, 3H, CH_3_), 7.42 (t, 1H, Ar, J = 7.4 Hz), 7.51 (t, 2H, Ar, J = 7.8 Hz), 7.60 (d, 2H, Ar, J = 7.6 Hz), 8.16 (s, 1H, N=CH). Anal. Calcd for C_12_H_9_N_3_O_2_: C, 63.43; H, 3.99; N, 18.49. Found C, 63.55; H, 3.99; N, 18.46.

##### 5-Amino-3-methyl-6-oxo-1-phenyl-1,6-dihydropyridazine-4-carboxylic acid methyl ester (**14**)

A mixture of **12** (6.21 mmol) and Et_3_N (0.8 mL) in 2 mL of CH_3_OH was heated at 60 °C for 2 h. After cooling, ice water (20 mL) was added and the suspension was extracted with CH_2_Cl_2_ (3 × 15 mL). Then the solvent was evaporated in vacuo to afford compound **14** which was purified by flash column chromatography using cyclohexane/ethyl acetate 1:1 as eluent. Yield = 80%; mp = 91–93 °C (Cyclohexane). ^1^H NMR (400 MHz, CDCl_3_) δ 2.53 (s, 3H, CH_3_), 3.95 (s, 3H, COOCH_3_), 7.39 (t, 1H, Ar, J = 7.4 Hz), 7.49 (t, 2H, Ar, J = 8.4 Hz), 7.65 (d, 2H, Ar, J = 8.4 Hz), 8.16 (exch br s, 2H, NH_2_). Anal. Calcd for C_13_H_13_N_3_O_3_: C, 60.22; H, 5.05; N, 16.21. Found C, 60.08; H, 5.06; N, 16.26.

#### 3.2.7. 5-Amino-3-methyl-6-oxo-1-phenyl-1,6-dihydropyridazine-4-carboxylic acid (**15**)

A mixture of **14** (2.12 mmol), ethanol (3 mL) and 6N NaOH (2 mL) was stirred at reflux for 30 min. After cooling, the solvent was evaporated under vacuum, cold water was added (2–3 mL) and the mixture was acidified with 6N HCl. The precipitate was recovered by vacuum filtration and crystallized from cyclohexane. Yield = 90%; mp = 214–216 °C (Cyclohexane). ^1^H NMR (400 MHz, DMSO-d_6_) δ 2.37 (s, 3H, CH_3_), 7.39 (t, 1H, Ar, J = 7.2 Hz), 7.46 (t, 2H, Ar, J = 8.0 Hz), 7.54 (d, 2H, Ar, J = 8.0 Hz), 8.25 (exch br s, 2H, NH_2_). Anal. Calcd for C_12_H_11_N_3_O_3_: C, 58.77; H, 4.52; N, 17.13. Found C, 58.61; H, 4.51; N, 17.16.

#### 3.2.8. 5-Amino-3-methyl-6-oxo-1-phenyl-1,6-dihydropyridazine-4-carboxylic acid amide (**16**)

A mixture of **15** (1.88 mmol), a catalytic amount of Et_3_N (0.1 mL) and SOCl_2_ (51 mmol) was refluxed for 30 min. After cooling, the excess of SOCl_2_ was removed in vacuo and the residue oil was dissolved in cold dry THF (1 mL). To this suspension a solution of 33% NH_3_ (2 mL) in 1.5 mL of dry THF was added and the mixture was stirred at room temperature for 15 min. After evaporation of the solvent, the mixture was diluted with cold water (20 mL) and the precipitate obtained was filtered and crystallized from ethanol. Yield = 80%; mp = 247–249 °C (EtOH). White coloured solid, ^1^H NMR (400 MHz, DMSO-d_6_) δ 2.21 (s, 3H, CH_3_), 7.35–7.40 (m, 1H, Ar), 7.45–7.51 (m, 5H, Ar), 7.65 (s, 1H, NH_2_), 7.88 (s, 1H, CONH_2_). ^13^C NMR (100 MHz, DMSO-d_6_) δ 167.39, 155.53, 143.12, 141.99, 141.63, 128.82, 127.99, 125.85, 110.42, 20.24. MS-ESI for C_12_H_12_N_4_O_2_ (Calcd, 244.10), [M + H]^+^ at *m/z* 244.95, t_R_ = 7.921. Anal. Calcd for C_12_H_12_N_4_O_2_: C, 59.01; H, 4.95; N, 22.94. Found C, 59.24; H, 4.97; N, 23.03.

#### 3.2.9. 5-Amino-3-methyl-6-oxo-1-phenyl-1,6-dihydropyridazine-4-carbonitrile (**17**)

Compound **17** was obtained starting from compound **16**, through the same procedure described for **7**. After dilution with cold water, the precipitate was recovered by filtration under *vacuum* and the solid obtained was purified by flash column chromatography using cyclohexane/ethyl acetate 1:1 as eluent. Yield = 20%; mp = 201–203 °C (EtOH). ^1^H NMR (400 MHz, DMSO-d_6_) δ 2.26 (s, 3H, CH_3_), 7.38 (ddd, 1H, J = 7.7, 5.5, 3.6 Hz, Ar), 7.42–7.51 (m, 4H, Ar). ^13^C NMR (100 MHz, DMSO-d_6_) δ 153.37, 149.50, 144.17, 141.60, 130.03, 127.68, 125.90, 115.14, 100.83, 20.47. MS-ESI for C_12_H_10_N_4_O (Calcd, 226.08), 226.96 *m/z* [M + H]^+^, 435.11 *m/z* [2M+H-H_2_O]^+^, 451.07 *m/z* [2M-H_2_+H]^+^. t_R_ =11.385. Anal. Calcd for C_12_H_10_N_4_O: C, 63.71; H, 4.46; N, 24.76. Found C, 63.96; H, 4.48; N, 24.85.

#### 3.2.10. 5-Acetyl-4-amino-2-phenylpyridazin-3(2H)-one (**18**)

Intermediate **13** (1.01 mmol) was suspended in 3.5 mL of EtOH, then 6.08 mmol of HCOONH_4_ and 40 mg of 10% Pd/C were added. The mixture was refluxed for 2 h and after cooling, CH_2_Cl_2_ (5 mL) was added. The solution was stirred for 5 min, then the catalyst was filtered off and the solvent was evaporated in vacuo to furnish desiderd compound **18**. Yield = 98%; mp = 181–183 °C (Cyclohexane). ^1^H NMR (400 MHz, CDCl_3_) δ 2.60 (s, 3H, COCH_3_), 6.95 (exch br s, 1H, NH_2_), 7.42 (t, 1H, Ar, J = 7.6 Hz), 7.51 (t, 2H, Ar, J = 7.6 Hz), 7.64 (d, 2H, Ar, J = 7.6 Hz), 8.13 (s, 1H, C6-H), 9.15 (exch br s, 1H, NH_2_). Anal. Calcd for C_12_H_11_N_3_O_2_: C, 62.87; H, 4.84; N, 18.33. Found C, 62.69; H, 4.83; N, 18.28.

#### 3.2.11. 6-Cyclohexyl-4-phenyl-6H-isoxazolo [3,4-d]pyridazin-7-one (**20**)

Compound **20** was obtained starting from **19** [34] adopting the general procedure described for compounds **12** and **13**, but using cyclohexyl hydrazine as reagent. The mixture was heated at 70 °C for 5 h. After dilution with ice-water, the precipitate was recovered by filtration under vacuum and crystallized from ethanol. Yield = 45%; mp = 211–213 °C (EtOH). ^1^H NMR (400 MHz, CDCl_3_) δ 1.27–1.33 (m, 1H, C_6_H_11_), 1.45–1.51 (m, 3H, C_6_H_11_), 1.75–1.80 (m, 1H, C_6_H_11_), 1.85–1.95 (m, 5H, C_6_H_11_), 5.05–5.10 (m, 1H, C_6_H_11_), 7.50–7.60 (m, 3H, Ar), 7.85 (d, 2H, Ar, J = 7.2 Hz), 9.30 (s, 1H, isoxazole). Anal. Calcd for C_17_H_17_N_3_O_2_: C, 69.14; H, 5.80; N, 14.23. Found C, 69.33; H, 4.82; N, 18.28.

#### 3.2.12. 5-Amino-1-cyclohexyl-6-oxo-3-phenyl-1,6-dihydropyridazine-4-carboxylic acid amide (**21**)

A mixture of **20** (0.64 mmol) and 33% NH_3_ was stirred at 120 °C for 3 h in a sealed/pressure vessel. After cooling, ice-water was added and the suspension was extracted with CH_2_Cl_2_ (3 × 15 mL). Evaporation of the solvent afforded the desired final compound. Yield = 20%; mp = 125–128 °C (Cyclohexane). ^1^H NMR (400 MHz, CDCl_3_) δ 1.20–1.25 (m, 1H, C_6_H_11_), 1.35–1.48 (m, 2H, C_6_H_11_), 1.60–1.65 (m, 2H, C_6_H_11_), 1.70–1.88 (m, 5H, C_6_H_11_), 4.77–4.82 (m, 1H, C_6_H_11_), 6.55 (exch br s, 2H, NH_2_), 7.25–7.31 (m, 3H, Ar), 7.44 (d, 2H, Ar, J = 7.6 Hz), 8.50 (exch br s, 2H, CONH_2_). Anal. Calcd for C_17_H_20_N_4_O_2_: C, 65.37; H, 6.45; N, 17.94. Found C, 65.52; H, 6.46; N, 17.99.

#### 3.2.13. 5-Amino-1-cyclohexyl-6-oxo-3-phenyl-1,6-dihydropyridazine-4-carbonitrile (**22**)

Compound **22** was obtained starting from compound **21**, through the same procedure described for **7** and **17**. After dilution with cold water, the precipitate was recovered by suction and the solid was purified by crystallization from etanol. Yield = 90%; mp = 170–172 °C (EtOH). Yellow coloured solid, ^1^H NMR (400 MHz, CDCl_3_) δ 1.23 (qt, 1H, J = 13.2, 3.3 Hz, CH_2_-cyclohexane), 1.46 (ttt, 2H, J = 13.8, 7.8, 3.0 Hz, CH_2_-cyclohexane), 1.71 (dt, 1H, J = 13.3, 3.4 Hz, CH_2_-cyclohexane), 1.84–1.91 (m, 6H, J = 4.4, 3.5 Hz, CH_2_-cyclohexane), 4.84–4.93 (m, 1H, CH_2_-cyclohexane), 7.45–7.50 (m, 3H, Ar), 7.72–7.74 (m, 2H, Ar). ^13^C NMR (100 MHz, CDCl_3_) δ 152.65, 148.93, 144.34, 135.02, 129.91, 128.75, 128.20, 114.83, 85.25, 58.19, 30.99, 25.60. MS-ESI for C_17_H_18_N_4_O (Calcd, 294.15), [M + H]^+^ at *m/z* 294.99, [M + ACN + H]^+^ at *m/z* 336.01, t_R_ = 17.509. Anal. Calcd for C_17_H_18_N_4_O: C, 69.37; H, 6.16; N, 19.03. Found C, 69.09; H, 6.13; N, 18.95.

#### 3.2.14. General Procedure for **24b, 24d-f**

A mixture of **23** [37] (0.80 mmol), K_2_CO_3_ (1.60 mmol) and 0.96–1.44 mmol of the appropriate alkyl or cycloalkyl bromide in anhydrous DMF (1 mL) was refluxed for 2–4 h. After cooling, the mixture was diluted with cold water (20 mL) and extracted with CH_2_Cl_2_ (3 × 15 mL). Evaporation of the solvent afforded the desired final compounds which were purified by flash column chromatography using cyclohexane/ethyl acetate 1:1 (for **24b,e,f**) or 1:2 (for **24d**) as eluent.

##### 4-Amino-2-cyclohexyl-6-phenylpyridazin-3(2H)-one (**24b**)

Yield = 21%; mp = 120–124 °C (EtOH). ^1^H NMR (400 MHz, CDCl_3_) δ 1.20–1.35 (m, 1H, C_6_H_11_), 1.40–1.50 (m, 2H, C_6_H_11_), 1.70–1.80 (m, 1H, C_6_H_11_), 1.90–2.05 (m, 6H, C_6_H_11_), 4.85–5.10 (m, 3H, 1H C_6_H_11_ + 2H NH_2_), 6.75 (s, 1H, -CH pyridaz.), 7.35–7.50 (m, 3H, Ar), 7.70 (d, 2H, Ar, J = 7.6 Hz). Anal. Calcd for C_16_H_19_N_3_O: C, 71.35; H, 7.11; N, 15.60. Found C, 71.52; H, 7.10; N, 15.56.

##### 4-Amino-2-isopropyl-6-phenylpyridazin-3(2H)-one (**24d**)

Yield = 85%; mp = 122–124 °C (EtOH). ^1^H NMR (400 MHz, CDCl_3_) δ 1.45 (d, 6H, CH(CH_3_)_2_, J = 6.8 Hz), 4.97 (exch br s, 2H, NH_2_), 5.41 (quin, 1H, CH(CH_3_)_2_, J = 6.8 Hz), 6.75 (s, 1H, -CH pyridaz.), 7.40–7.50 (m, 3H, Ar), 7.81 (d, 2H, Ar, J = 7.6 Hz). Anal. Calcd for C_13_H_15_N_3_O: C, 68.10; H, 6.59; N, 18.33 Found C, 68.31; H, 6.60; N, 18.29.

##### 4-Amino-6-phenyl-2-propylpyridazin-3(2H)-one (**24e**)

Yield = 83%; mp = 79–81 °C (EtOH). ^1^H NMR (400 MHz, CDCl_3_) δ 1.02 (t, 3H, CH_2_CH_2_CH_3_, J = 7.2 Hz), 1.93 (sex, 2H, CH_2_CH_2_CH_3_, J = 7.2 Hz), 4.23 (t, 2H, CH_2_CH_2_CH_3_, J = 7.2 Hz), 4.99 (exch br s, 2H, NH_2_), 6.73 (s, 1H, -CH pyridaz.), 7.38–7.50 (m, 3H, Ar), 7.78 (d, 2H, Ar, J = 8.0 Hz). Anal. Calcd for C_13_H_15_N_3_O: C, 68.10; H, 6.59; N, 18.33 Found C, 68.28; H, 6.60; N, 18.31.

##### 4-Amino-2-butyl-6-phenylpyridazin-3(2H)-one (**24f)**

Yield = 94%; mp = 67–69 °C (EtOH). ^1^H NMR (400 MHz, CDCl_3_) δ 1.00 (t, 3H, CH_2_CH_2_CH_2_CH_3_, J = 7.2 Hz), 1.45 (m, 2H, CH_2_CH_2_CH_2_CH_3_), 1.87 (m, 2H, CH_2_CH_2_CH_2_CH_3_), 4.26 (t, 2H, CH_2_CH_2_CH_2_CH_3_, J = 7.2 Hz), 4.99 (exch br s, 2H, NH_2_), 6.75 (s, 1H, -CH pyridaz.), 7.38–7.50 (m, 3H, Ar), 7.76 (d, 2H, Ar, J = 8.0 Hz). Anal. Calcd for C_14_H_17_N_3_O: C, 69.11; H, 7.04; N, 17.27 Found C, 69.29; H, 7.03; N, 17.32.

#### 3.2.15. General Procedure for Compounds **25a–f**

To a cooled (0 °C) and stirred suspension of the appropriate pyridazinone **24a–f** (0.65 mmol) in anhydrous THF (1–3 mL), anhydrous sodium acetate (1.55 mmol) and triphosgene (2.26 mmol) were added. The mixture was stirred for 10 min at room temperature and refluxed for 2 h. Then, the suspension was cooled to 0 °C and 1 mL of 33% NH_3_ was added and the mixture was stirred for 30–90 min at room temperature. After evaporation of the solvent, ice/cold water was added (15 mL) and the precipitate obtained was recovered by filtration under vacuum and purified by crystallization from ethanol to obtain the pure samples of **25a–f.**

##### (2-Methyl-3-oxo-6-phenyl-2,3-dihydro-pyridazin-4-yl)urea (**25a**)

Yield = 65%; mp > 300 °C (EtOH). ^1^H NMR (400 MHz, DMSO-d_6_) δ 3.77 (s, 3H, CH_3_), 7.42–7.51 (m, 3H, Ar), 7.73–7.76 (m, 2H, Ar), 8.35 (s, 1H, Ar), 8.98 (s, 1H, NHCONH_2_). ^13^C NMR (100 MHz, DMSO-d_6_) δ 155.69, 155.11, 145.12, 137.92, 135.75, 129.39, 129.09, 126.02, 106.62, 20.93. MS-ESI for C_12_H_12_N_4_O_2_ (Calcd, 244.10), [M + H]^+^ at *m/z* 244.95, t_R_ = 11.531. Anal. Calcd for C_12_H_12_N_4_O_2_: C, 59.01; H, 4.95; N, 22.94 Found C, 59.24; H, 4.97; N, 23.03.

##### (2-Cyclohexyl-3-oxo-6-phenyl-2,3-dihydro-pyridazin-4-yl)urea (**25b**)

Yield = 35%; mp = 261–263 °C (EtOH). ^1^H NMR (400 MHz, CDCl_3_) δ 1.18–1.31 (m, 1H, C_6_H_11_), 1.40–1.51 (m, 2H, C_6_H_11_), 1.64–1.72 (m, 1H, C_6_H_11_), 1.70–1.90 (m, 6H, C_6_H_11_), 4.87 (m, 1H, C_6_H_11_), 6.80 (exch br s, 2H, NH_2_), 7.45–7.55 (m, 3H, Ar), 7.79 (d, 2H, Ar, J = 7.6 Hz), 8.37 (s, 1H, -CH pyridaz.), 8.96 (exch br s, 1H, NHCONH_2_). Anal. Calcd for C_17_H_20_N_4_O_2_: C, 65.37; H, 6.45; N, 17.94. Found C, 65.18; H, 6.46; N, 17.91.

##### (2-Ethyl-3-oxo-6-phenyl-2,3-dihydro-pyridazin-4-yl)urea (**25c**)

Yield = 25%; mp = 270–271 °C (EtOH). White coloured solid, ^1^H NMR (400 MHz, DMSO-d_6_) δ 1.33 (t, 3H, J = 7.1 Hz, NCH_2_CH_3_), 4.20 (q, 2H, J = 7.2 Hz, NCH_2_), 6.70 (exch br s, 2H, NHCONH_2_) 7.47 (dt, 3H, J = 13.1, 7.1 Hz, Ar), 7.73 (dd, 2H, J = 23.5, 7.6 Hz, Ar), 8.34 (s, 1H, Ar), 8.98 (s, 1H, NHCONH_2_). ^13^C NMR (100 MHz, DMSO-d_6_) δ 155.36, 154.51, 150.89, 145.06, 137.97, 135.75, 128.88, 125.84, 106.29, 47.00, 13.39. MS-ESI for C_13_H_14_N_4_O_2_ (Calcd, 258.11), [M + H]^+^ at *m/z* 259.02, 215.90 *m/z* [M-CONH_2_ + H]^+^. t_R_ = 12.090. Anal. Calcd for C_13_H_14_N_4_O_2_: C, 60.45; H, 5.46; N, 21.69. Found C, 60.21; H, 5.44; N, 21.60.

##### (2-Isopropyl-3-oxo-6-phenyl-2,3-dihydro-pyridazin-4-yl)urea (**25d**)

Yield = 68%; mp = 260–263 °C (EtOH). White coloured solid, ^1^H NMR (400 MHz, DMSO-d_6_) δ 1.36 (d, 6H, J = 6.7 Hz, NCH(CH_3_)_2_), 5.24 (q, 1H, J = 6.6 Hz, NCH), 7.47 (dt, 2H, J = 15.9, 7.2 Hz, Ar), 7.67–7.79 (m, 3H, Ar), 8.34 (s, 1H, Ar), 8.95 (s, 1H, NHCONH_2_). ^13^C NMR (100 MHz, DMSO-d_6_) δ 155.69, 154.31, 144.75, 137.69, 136.13, 132.04, 129.30, 129.08, 127.97, 125.91, 105.93, 49.71, 20.96. MS-ESI for C_14_H_16_N_4_O_2_ (Calcd, 272.13), [M + H]^+^ at *m/z* 272.95, t_R_ = 14.248. Anal. Calcd for C_14_H_16_N_4_O_2_: C, 61.75; H, 5.92; N, 20.58. Found C, 61.99; H, 5.94; N, 20.66.

##### (3-Oxo-6-phenyl-2-propyl-2,3-dihydro-pyridazin-4-yl)urea (**25e**)

Yield = 60%; mp = 273–275 °C (EtOH). White coloured solid, ^1^H NMR (400 MHz, DMSO-d_6_) δ 0.89 (td, 3H, J = 7.4, 2.7 Hz, NCH_2_CH_2_CH_3_), 1.79 (q, 2H, J = 7.3 Hz, NCH_2_CH_2_), 4.13 (t, 2H, J = 7.1 Hz, NCH_2_), 7.42–7.51 (m, 2H, Ar), 7.69 (d, 1H, J = 5.6 Hz, Ar), 7.73–7.76 (m, 2H, Ar), 8.33 (s, 1H, Ar), 8.96 (s, 1H, NHCONH_2_). ^13^C NMR (100 MHz, DMSO-d_6_) δ 155.68, 154.88, 145.06, 137.94, 135.87, 132.03, 129.36, 129.07, 128.06, 126.03, 106.37, 53.19, 21.39, 11.13. MS-ESI for C_14_H_16_N_4_O_2_ (Calcd, 272.13), [M + H]^+^ at *m/z* 272.95, 229.90 *m/z* [M-CONH_2_ + H]^+^. t_R_ = 14.037. Anal. Calcd for C_14_H_16_N_4_O_2_: C, 61.75; H, 5.92; N, 20.58. Found C, 61.99; H, 5.94; N, 20.66.

##### (2-Butyl-3-oxo-6-phenyl-2,3-dihydro-pyridazin-4-yl)urea (**25f**)

Yield = 85%; mp = 265–267 °C (EtOH). White solid, ^1^H NMR (400 MHz, DMSO-d_6_ + D_2_O) δ 0.88 (t, 3H, J = 7.4 Hz, CH_3_), 1.27 (q, 2H, J = 7.3 Hz, CH_2_CH_3_), 1.73 (q, 2H, J = 7.2 Hz, CH_2_CH_2_CH_3_), 4.13–4.16 (m, 2H, CH_2_ArN), 7.41–7.51 (m, 3H, Ar), 7.73–7.75 (m, 2H, Ar), 8.08 (s, 1H, NHCONH_2_), 8.36 (s, 1H, Ar), 9.10 (s, 2H, CONH_2_). ^13^C NMR (100 MHz, DMSO-d_6_) δ 154.80, 154.60, 137.56, 135.69, 129.20, 128.94, 127.78, 125.79, 118.03, 106.44, 51.11, 29.92, 19.24, 13.53. MS-ESI for C_15_H_18_N_4_O_2_ (Calcd, 286.14), [M + H]^+^ at *m/z* 286.94, t_R_ = 31.162. Anal. Calcd for C_15_H_18_N_4_O_2_: C, 62.92; H, 6.34; N, 19.57. Found C, 62.66; H, 6.31; N, 19.49.

#### 3.2.16. 4-Amino-6-phenylpyridazine-3(2H)-thione (**26**)

A mixture of **23** [37] (0.86 mmol) and Lawesson’s reagent (1.71 mmol) in anhydrous toluene (2–3 mL) was heated at 90 °C for 5 h. After cooling the solvent was evaporated under vacuum, cold water was added (10 mL) and the mixture was extracted with CH_2_Cl_2_ (3 × 15 mL). Evaporation of the solvent afforded **26** which was purified by flash column chromatography using CH_2_Cl_2_/CH_3_OH 10:1 as eluent. Yield = 63%; mp = 175–178 °C (Cyclohexane). ^1^H NMR (400 MHz, CDCl_3_) δ 5.77 (exch br s, 2H, NH_2_), 6.80 (s, 1H, -CH pyridaz.), 7.45–7.55 (m, 3H, Ar), 7.75–7.81 (m, 2H, Ar). Anal. Calcd for C_10_H_9_N_3_S: C, 59.09; H, 4.46; N, 20.67. Found C, 59.23; H, 4.45; N, 20.62.

#### 3.2.17. 3-Methylsulfanyl-6-phenyl-pyridazin-4-ylamine (**27**)

Compound **27** was obtained, starting from compound **26**, through the general procedure described for **24b** and **24d–f**. After dilution with cold water, the precipitate was recovered by suction and purified by crystallization. Yield = 40%; mp = 168–170 °C (Cyclohexane). Greenish colour solid, ^1^H NMR (400 MHz, DMSO-d_6_) δ 2.64 (s, 3H, SCH_3_), 6.27 (exch br s, 2H, NH_2_), 7.00 (s, 1H, Ar), 7.46 (dt, 3H, ArH, J = 12.6, 6.9 Hz), 7.90–7.93 (m, 2H, Ar). ^13^C NMR (100 MHz, DMSO-d_6_) δ155.31, 147.71, 144.50, 137.20, 129.38, 129.02, 126.51, 102.80, 12.72. MS-ESI for C_11_H_11_N_3_S (Calcd, 217.07), [M + H]^+^ at *m/z* 217.86, t_R_ = 9.922. Anal. Calcd for C_11_H_11_N_3_S: C, 60.80; H, 5.10; N, 19.34. Found C, 60.56; H, 5.08; N, 19.26.

#### 3.2.18. (3-Oxo-6-phenyl-2,3-dihydro-pyridazin-4-yl)urea (**28**)

Compound **28** was obtained, starting from **23** [37], through the same procedure described for **25a–f**. Yield = 85%; mp >300 °C (EtOH). ^1^H NMR (400 MHz, DMSO-d_6_) δ 7.41–7.50 (m, 3H, Ar), 7.72–7.75 (m, 2H, Ar), 8.33 (s, 1H, Ar), 8.94 (s, 1H, NHCONH_2_), 13.21 (s, 1H, ArNH). ^13^C NMR (100 MHz, DMSO-d_6_) δ156.00, 155.33, 145.54, 139.57, 138.27, 135.77, 134.50, 128.87, 125.70, 106.97. MS-ESI for C_11_H_10_N_4_O_2_ (Calcd, 230.08), [M + H]^+^ at *m/z* 230.88, t = 10.042. Anal. Calcd for C_11_H_10_N_4_O_2_: C, 57.39; H, 4.38; N, 24.34. Found C, 57.62; H, 4.39; N, 24.44.

#### 3.2.19. General Procedure for Compounds **29a–d**

A mixture of **24a** [38] (0.39 mmol) and the appropriate R-anhydride (13.1 mmol) in 1 mL of pyridine was heated at 140 °C for 5 h in a sealed/pressure vessel. After cooling, ice/cold water was added (50 mL), the precipitate was recovered by filtration under vacuum and purified by crystallization from ethanol to obtain the desired compounds.

##### N-(2-Methyl-3-oxo-6-phenyl-2,3-dihydro-pyridazin-4-yl)acetamide (**29a**)

Yield = 90%; mp = 211–212 °C (EtOH). Brownish black coloured solid, ^1^H NMR (400 MHz, CDCl_3_) δ 2.28 (s, 3H, CH_3_CONH), 3.91 (s, 3H, CH_3_ArN), 7.42–7.48 (m, 3H, Ar), 7.80–7.83 (m, 2H, Ar), 8.61 (s, 1H, ArH). ^13^C NMR (100 MHz, CDCl_3_) δ 196.96, 155.66, 146.61, 135.61, 135.59, 129.59, 128.97, 126.46, 110.82, 40.91, 24.98. MS-ESI for C_13_H_13_N_3_O_2_ (Calcd, 243.10), [M + H]^+^ at *m/z* 243.90, t_R_ = 13.311. Anal. Calcd for C_13_H_13_N_3_O_2_: C, 64.19; H, 5.39; N, 17.27. Found C, 64.45; H, 5.41; N, 17.34.

##### N-(2-Methyl-3-oxo-6-phenyl-2,3-dihydro-pyridazin-4-yl)propionamide **(29b**)

Yield = 93%; mp = 210–211 °C (EtOH). Ash coloured solid, ^1^H NMR (400 MHz, CDCl_3_) δ 1.26 (td, 3H, J = 7.5, 1.1 Hz, CH_3_CH_2_CONH), 2.49–2.55 (m, 2H, CH_2_CONH), 3.92 (s, 3H, CH_3_ArN), 7.41–7.47 (m, 3H, Ar), 7.81–7.84 (m, 2H, Ar), 8.65 (s, 1H, Ar). ^13^C NMR (100 MHz, CDCl_3_) δ 173.77, 155.73, 146.62, 135.63, 135.60, 129.58, 128.95, 126.43, 110.76, 40.90, 31.00, 9.24. MS-ESI for C_14_H_15_N_3_O_2_ (Calcd, 257.12), [M + H]^+^ at *m/z* 257.90, t_R_ = 14.604. Anal. Calcd for C_14_H_15_N_3_O_2_: C, 65.36; H, 5.88; N, 16.33. Found C, 65.10; H, 5.90; N, 16.39.

##### N-(2-Methyl-3-oxo-6-phenyl-2,3-dihydro-pyridazin-4-yl)isobutyramide (**29c**)

Yield = 95%; mp = 146–148 °C (EtOH). Brown coloured solid, ^1^H NMR (400 MHz, CDCl_3_) δ 1.28 (dd, 6H, J = 6.9, 1.2 Hz, (CH_3_)_2_CHCONH), 2.64–2.71 (m, 1H, CHCONH), 3.92 (s, 3H, CH_3_ArN), 7.41–7.46 (m, 3H, Ar), 7.81–7.85 (m, 2H, Ar), 8.66 (s, 1H, Ar). ^13^C NMR (100 MHz, CDCl_3_) δ 177.10, 155.82, 146.61, 135.71, 135.59, 129.57, 128.94, 126.42, 110.83, 40.87, 36.95, 19.45. MS-ESI for C_15_H_17_N_3_O_2_ (Calcd, 271.13), [M + H]^+^ at *m/z* 272.04, t_R_ = 16.829. Anal. Calcd for C_15_H_17_N_3_O_2_: C, 66.40; H, 6.32; N, 15.49. Found C, 66.66; H, 6.34; N, 15.55.

##### N-(2-Methyl-3-oxo-6-phenyl-2,3-dihydro-pyridazin-4-yl)butyramide (**29d**)

Yield = 92%; mp = 187–189 °C (EtOH). ^1^H NMR (400 MHz, CDCl_3_) δ 1.05 (t, 3H, CH_2_CH_2_CH_3_, J = 7.2 Hz), 1.80 (sex, 2H, CH_2_CH_2_CH_3_, J = 7.2 Hz), 2.49 (q, 2H, CH_2_CH_2_CH_3_, J = 7.2 Hz), 3.94 (s, 3H, N-CH_3_), 7.45–7.50 (m, 3H, Ar), 7.85 (d, 2H, Ar, J = 7.6 Hz), 8.63 (exch br s, 1H, NH), 8.68 (s, 1H, -CH pyridaz.). Anal. Calcd for C_15_H_17_N_3_O_2_: C, 66.40; H, 6.32; N, 15.49. Found C, 66.25; H, 6.31; N, 15.44.

#### 3.2.20. General procedure for compounds **30a,b** and **33**

A mixture of compound **24a** [38] (0.79 mmol), the appropriate R-phenylboronic acid (0.79 mmol), copper acetate (1.19 mmol) and triethylamine (1.59 mmol) in CH_2_Cl_2_ (5 mL) was stirred at room temperature for 3–12 h. After evaporation of the solvent, ethyl acetate was added (15–20 mL) and the solution was extracted first with 33% NH_3_ (3 × 5 mL) and then with water (2 × 5 mL). The organic layer was evaporated under vacuum and the residue was purified by crystallization from ethanol. 

#### 3.2.21. 3-(2-Methyl-3-oxo-6-phenyl-2,3-dihydro-pyridazin-4-ylamino)benzonitrile (**30a**)

Yield = 60%; mp = 234–235 °C (EtOH). White coloured solid, ^1^H NMR (400 MHz, DMSO-d_6_) δ 3.80 (s, 3H, CH_3_), 7.23 (s, 1H, Ar), 7.42–7.48 (m, 3H, Ar), 7.55–7.59 (m, 2H, Ar), 7.80 (dd, 3H, J = 8.0, 1.8 Hz, ArCN), 7.86 (d, 1H, J = 1.8 Hz, ArCN), 9.03 (exch br s, 1H, NH). ^13^C NMR (100 MHz, DMSO-d_6_) δ 193.78, 157.05, 151.81, 140.85, 136.62, 129.18, 126.83, 111.09, 100.21, 23.94. MS-ESI for C_18_H_14_N_4_O (Calcd, 302.12), [M + H]^+^ at *m/z* 302.90, [M + ACN + H]^+^ at *m/z* 343.92, t_R_ = 16.247. Anal. Calcd for C_18_H_14_N_4_O: C, 71.51; H, 4.67; N, 18.53. Found C, 71.22; H, 4.65; N, 18.45.

#### 3.2.22. 2-(2-Methyl-3-oxo-6-phenyl-2,3-dihydro-pyridazin-4-ylamino)benzonitrile (**30b**)

Yield = 32%; mp = 178–180 °C (EtOH). White coloured solid, ^1^H NMR (400 MHz, CDCl_3_) δ 3.95 (s, 3H, CH_3_), 7.12 (s, 1H, Ar), 7.41–7.47 (m, 3H, ArCN), 7.55 (d, 1H, J = 8.3 Hz, ArCN), 7.65 (td, 1H, J = 7.8, 1.6 Hz, Ar), 7.72 (ddd, 3H, J = 7.6, 3.6, 1.7 Hz, Ar), 7.96 (exch br s, 1H, NH). ^13^C NMR (100 MHz, CDCl_3_) δ 147.91, 138.56, 13.22, 129.42, 128.97, 126.44, 124.63, 121.19, 114.68, 100.94, 40.59. MS-ESI for C_18_H_14_N_4_O (Calcd, 302.12), [M + H]^+^ at *m/z* 302.97, [M + ACN + H]^+^ at *m/z* 344.06, t_R_ = 16.180. Anal. Calcd for C_18_H_14_N_4_O: C, 71.51; H, 4.67; N, 18.53. Found C, 71.22; H, 4.65; N, 18.45.

#### 3.2.23. 4-(2-Methyl-3-oxo-6-phenyl-2,3-dihydro-pyridazin-4-ylamino)benzoic acid ethyl ester (**33**)

Yield = 80%; mp = 171–172 °C (Cyclohexane). ^1^H NMR (400 MHz, CDCl_3_) 1.42 (t, 3H, CH_2_CH_3_, J = 7.2 Hz), 3.96 (s, 3H, CH_3_), 4.41 (q, 2H, CH_2_CH_3_, J = 7.2 Hz), 7.30–7.40 (m, 3H, 2H Ar + CH pyridaz.), 7.45–7.50 (m, 3H, Ar), 7.77 (d, 2H, Ar, J = 8.8 Hz), 7.90 (exch br s, 1H, NH), 8.12 (d, 2H, Ar, J = 8.8 Hz). Anal. Calcd for C_20_H_19_N_3_O_3_: C, 68.75; H, 5.48; N, 12.03. Found C, 68.58; H, 5.47; N, 12.06.

#### 3.2.24. General Procedure for Compounds **31a,b**

A mixture of appropriate pyridazin-benzonitrile **30a** or **30b** (0.165 mmol) and 80% H2SO4 (2 mL) was stirred at 80 °C for 4 h. After cooling, ice/cold water (2–3 mL) was slowly added, the precipitate obtained was recovered by filtration under vacuum and purified by crystallization.

#### 3.2.25. 3-(2-Methyl-3-oxo-6-phenyl-2,3-dihydro-pyridazin-4-ylamino)benzamide (**31a**)

Yield = 93%; mp = 214–216 °C (Cyclohexane). ^1^H NMR (400 MHz, DMSO-d_6_) δ 3.81 (s, 3H, CH_3_), 7.13 (s, 1H, -CH pyridaz.), 7.40–7.50 (m, 5H, 4H Ar + 1H CONH_2_), 7.60 (d, 1H, Ar, J = 9.2 Hz), 7.64 (d, 1H, Ar, J = 7.2 Hz), 7.76 (d, 2H, Ar, J = 8.0 Hz), 7.91 (s, 1H, Ar), 8.02 (exch br s, 1H, CONH_2_), 8.93 (exch br s, 1H, NH). Anal. Calcd for C_18_H_16_N_4_O_2_: C, 67.49; H, 5.03; N, 17.49. Found C, 67.36; H, 5.04; N, 17.53.

#### 3.2.26. 2-(2-Methyl-3-oxo-6-phenyl-2,3-dihydro-pyridazin-4-ylamino)benzamide (**31b**)

Yield = 95%; mp = 140–142 °C (Cyclohexane). ^1^H NMR (400 MHz, DMSO-d_6_) δ 3.80 (s, 3H, CH_3_), 7.15 (t, 1H, Ar, J = 7.6 Hz), 7.38 (s, 1H, -CH pyridaz.), 7.43–7.50 (m, 3H, Ar), 7.57 (t, 1H, Ar, J = 7.6 Hz), 7.66 (exch br s, 1H, CONH_2_), 7.77 (t, 2H, Ar, J = 9.2 Hz), 7.84 (d, 2H, Ar, J = 6.8 Hz), 8.17 (exch br s, 1H, CONH_2_), 10.68 (exch br s, 1H, NH). Anal. Calcd for C_18_H_16_N_4_O_2_: C, 67.49; H, 5.03; N, 17.49. Found C, 67.36; H, 5.04; N, 17.53.

#### 3.2.27. 4-Amino-2-methyl-6-phenylpyridazine-3(2H)-thione (**32**)

Compound **32** was obtained, starting from compound **24a** [38], through the same procedure described for 26. In this case, the mixture was refluxed for 10 h. After cooling, ice/cold water was added. The precipitate was recovered by suction and purified by flash column chromatography using cyclohexane/ethyl acetate 1:1 as eluent. Yield = 85%; mp = 134–135 °C (EtOH). ^1^H NMR (400 MHz, CDCl_3_) δ 4.38 (s, 3H, CH_3_), 5.90 (exch br s, 2H, NH_2_), 6.78 (s, 1H, -CH pyridaz.), 7.45–7.50 (m, 3H, Ar), 7.80–7.85 (m, 2H, Ar). Anal. Calcd for C_11_H_11_N_3_S: C, 60.80; H, 5.10; N, 19.34. Found C, 60.97; H, 5.11; N, 19.30.

#### 3.2.28. 4-(2-Methyl-3-oxo-6-phenyl-2,3-dihydro-pyridazin-4-ylamino)benzoic acid (**34**)

Compound **34** was obtained through the general procedure described for 15. After cooling, the mixture was acidified with 6N HCl and the final product was filtered off to obtain the desired compound. Yield = 90%; mp = 280–281 °C (Diethyl ether). ^1^H NMR (400 MHz, DMSO-d_6_) δ 3.82 (s, 3H, CH_3_), 7.37 (s, 1H, -CH pyridaz.), 7.40–7.50 (m, 3H, Ar), 7.58 (d, 2H, Ar, J = 8.8 Hz), 7.84 (d, 2H, Ar, J = 8.4 Hz), 7.95 (d, 2H, Ar, J = 8.4 Hz), 9.16 (exch br s, 1H, NH), 12.78 (exch br s, 1H, OH). Anal. Calcd for C_18_H_15_N_3_O_2_: C, 67.28; H, 4.71; N, 13.08. Found C, 67.44; H, 4.71; N, 13.05.

#### 3.2.29. 4-[4-(4-Acetyl-piperazine-1-carbonyl)-phenylamino]-2-methyl-6-phenylpyridazin-3(2H)-one (**35**)

Compound **35** was obtained starting from 34 through the same procedure described for **4a,b**. In this case the mixture was stirred at room temperature for 40 min. After cooling, THF was removed in vacuo and cold water was added (10 mL). The crude precipitate was recovered by filtration under vacuum and purified by crystallization. Yield = 94%; mp = 213–215 °C (Cyclohexane). Ligrownown solid, ^1^H NMR (400 MHz, CDCl_3_) δ 2.14 (s, 3H, CH_3_CONH), 3.94 (s, 3H, CH_3_), 3.60 (d, 8H, J = 46.0 Hz, 2 × NCH_2_CH_2_N), 7.22 (s, 1H, Ar), 7.33 (d, 2H, J = 7.9 Hz, Ar), 7.47 (dd, 6H, J = 24.3, 7.9 Hz, Ar), 7.71–7.80 (m, 2H, NH + Ar). ^13^C NMR (100 MHz, CDCl_3_) δ 170.15, 169.37, 156.24, 146.00, 140.96, 139.58, 136.38, 130.72, 129.39, 129.32, 128.96, 126.42, 120.74, 99.58, 40.75, 21.55. MS-ESI for C_24_H_25_N_5_O_3_ (Calcd, 431.20), [M + H]^+^ at *m/z* 432.10, [M + Na]^+^ at *m/z* 454.08, t_R_ = 13.347. Anal. Calcd for C_24_H_25_N_5_O_3_: C, 66.81; H, 5.84; N, 16.23. Found C, 66.54; H, 5.82; N, 16.16.

#### 3.2.30. General procedure for compounds **37a–d** and **42a,b**

A suspension of appropriate pyridazinone **36a–d** (1.29 mmol), commercially available, and hydrazine hydrate (48 mmol) was stirred in a sealed/pressure vessel at 180–200 °C for 6–12 h. After cooling, ice-cold water was added (15 mL) and the precipitate obtained was recovered by filtration under vacuum to obtain the desired compounds **37a–d.** To obtain compounds **42a,b** we adopted the same procedure, using **41a,b** (**41a**, [39]) as starting materials.

#### 3.2.31. 4-Amino-6-thiophen-3-yl-pyridazin-3(2H)-one (**37a**)

Yield = 52%; mp > 300 °C (EtOH). ^1^H NMR (400 MHz, DMSO-d_6_) δ 6.40 (exch br s, 2H, NH_2_), 6.68 (s, 1H, -CH pyridaz.), 7.45 (dd, 1H, thiophene, J_1_ = 1.2 Hz and J_2_ = 4.8 Hz), 7.60 (dd, 1H, thiophene, J_1_ = 2.8 Hz and J_2_ = 4.8 Hz), 7.81 (ds, 1H, thiophene, J = 1.2 Hz), 12.54 (exch br s, 1H, NH). Anal. Calcd for C_8_H_7_N_3_OS: C, 49.73; H, 3.65; N, 21.75. Found C, 49.61; H, 3.65; N, 21.69.

#### 3.2.32. 4-Amino-6-cyclohexylpyridazin-3(2H)-one (**37b**)

Yield = 46%; mp = 284–287 °C (EtOH). ^1^H NMR (400 MHz, DMSO-d_6_) δ 1.20–1.39 (m, 5H, C_6_H_11_), 1.72–1.83 (m, 5H, C_6_H_11_), 2.30–2.35 (m, 1H, C_6_H_11_), 6.14 (s, 1H, CH pyridaz.), 6.18 (exch br s, 2H, NH_2_), 12.24 (exch br s, 1H, NH). Anal. Calcd for C_10_H_15_N_3_O: C, 62.15; H, 7.82; N, 21.74. Found C, 62.29; H, 7.80; N, 21.79.

#### 3.2.33. 4-Amino-6-isopropylpyridazin-3(2H)-one **(37c**)

Yield = 40%; mp = 246–248 °C (EtOH). ^1^H NMR (400 MHz, DMSO-d_6_) δ 1.11 (d, 6H, CH(CH_3_)_2_, J = 7.2 Hz), 2.67 (m, 1H, CH(CH_3_)_2_), 6.16 (s, 1H, -CH pyridaz.), 6.18 (exch br s, 2H, NH_2_), 12.23 (exch br s, 1H, NH). Anal. Calcd for C_7_H_11_N_3_O: C, 54.89; H, 7.24; N, 27.43. Found C, 54.76; H, 7.23; N, 27.51.

#### 3.2.34. 4-Amino-6-benzylpyridazin-3(2H)-one (**37d**)

Yield = 42%; mp = 247–250 °C (EtOH). ^1^H NMR (400 MHz, DMSO-d_6_) δ 3.70 (s, 2H, CH_2_Ph), 6.04 (s, 1H, -CH pyridaz.), 6.22 (exch br s, 2H, NH_2_), 7.20–7.40 (m, 5H, Ar), 12.31 (exch br s, 1H, NH). Anal. Calcd for C_11_H_11_N_3_O: C, 65.66; H, 5.51; N, 20.88. Found C, 65.84; H, 5.50; N, 20.83.

#### 3.2.35. 4-Amino-6-(2-hydroxyphenyl)-2-methylpyridazin-3(2H)-one (**42a**)

Yield = 58%; mp = 212–213 °C (EtOH). White coloured solid, ^1^H NMR (400 MHz, CDCl_3_) δ 3.85 (s, 3H, CH_3_), 6.84 (s, 1H, Ar), 6.92 (td, 1H, J = 7.7, 1.2 Hz, Ar), 7.00–7.05 (m, 1H, Ar), 7.29 (td, 1H, J = 8.3, 7.8, 1.6 Hz, Ar), 7.58–7.50 (m, 1H, Ar). ^13^C NMR (100 MHz, CDCl_3_) δ 157.81, 131.24, 126.35, 119.47, 118.30, 98.93, 29.87. MS-ESI for C_11_H_11_N_3_O_2_ (Calcd, 217.08), [M + H]^+^ at *m/z* 217.93, t_R_ = 11.693. Anal. Calcd for C_11_H_11_N_3_O_2_: C, 60.82; H, 5.10; N, 19.34. Found C, 60.57; H, 5.08; N, 19.26.

#### 3.2.36. 4-Amino-6-(4-aminophenyl)-2-methylpyridazin-3(2H)-one (**42b)**

Yield = 55%; mp = 208–209 °C (Cyclohexane). ^1^H NMR (400 MHz, DMSO-d_6_) δ 3.65 (s, 3H, N-CH_3_), 5.35 (exch br s, 2H, NH_2_), 6.33 (exch br s, 2H, Ph-NH_2_), 6.59 (d, 2H, Ar, J = 8.0 Hz), 6.63 (s, 1H, -CH pyridaz.), 7.42 (d, 2H, Ar, J = 8.0). Anal. Calcd for C_11_H_11_N_4_O: C, 61.10; H, 5.59; N, 25.91. Found C, C, 61.27; H, 5.58; N, 25.85.

#### 3.2.37. General Procedure for Compounds **38a–d**

A mixture of the appropriate pyridazinone **37a–d** (0.67 mmol), K_2_CO_3_ (1.34 mmol) and CH_3_I (1.01 mmol) in anhydrous DMF (1.5 mL) was stirred at 80 °C for 1–4 h. After cooling, the mixture was diluted with cold water (15 mL) and compound **38a** was recovered by suction and crystallized from ethanol. For compounds **38b–d** the suspension was extracted with CH_2_Cl_2_ (3 × 15 mL) and the solvent was evaporated in vacuo. The final compounds were purified by flash column chromatography using cyclohexane/ethyl acetate 1:2 (for **38b,d**) or CH_2_Cl_2_/CH_3_OH 9.5:0.5 (for **38c**) as eluents.

#### 3.2.38. 4-Amino-2-methyl-6-thiophen-3-yl-pyridazin-3(2H)-one (**38a**)

Yield = 62%; mp = 178–179 °C (EtOH). ^1^H NMR (400 MHz, DMSO-d_6_) δ 3.67 (s, 3H, N-CH_3_), 6.50 (exch br s, 2H, NH_2_), 6.68 (s, 1H, -CH pyridaz.), 7.47 (d, 1H, thiophene, J = 4.8 Hz), 7.61 (m, 1H, thiophene), 7.84 (s, 1H, thiophene). Anal. Calcd for C_9_H_9_N_3_OS: C, 52.16; H, 4.38; N, 20.27. Found C, 52.05; H, 4.37; N, 20.22.

#### 3.2.39. 4-Amino-6-cyclohexyl-2-methylpyridazin-3(2H)-one (**38b**)

Yield = 58%; oil. ^1^H NMR (400 MHz, CDCl_3_) δ 1.30–1.43 (m, 5H, C_6_H_11_), 1.68–1.92 (m, 5H, C_6_H_11_), 2.40 (m, 1H, C_6_H_11_), 3.75 (s, 3H, N-CH_3_), 4.91 (exch br s, 2H, NH_2_), 6.21 (s, 1H, -CH pyridaz.). Anal. Calcd for C_11_H_17_N_3_O: C, 63.74; H, 8.27; N, 20.27. Found C, 63.87; H, 8.29; N, 20.23.

#### 3.2.40. 4-Amino-6-isopropyl-2-methyl-2H-pyridazin-3-one (**38c**)

Yield = 49%; oil. ^1^H NMR (400 MHz, CDCl_3_) δ 1.18 (d, 6H, CH(CH_3_)_2_, J = 7.2 Hz), 2.75 (m, 1H, CH(CH_3_)_2_), 3.74 (s, 3H, N-CH_3_), 4.96 (exch br s, 2H, NH_2_), 6.21 (s, 1H, -CH pyridaz.). Anal. Calcd for C_8_H_13_N_3_O: C, 57.46; H, 7.84; N, 25.13. Found C, 57.58; H, 7.82; N, 25.07.

#### 3.2.41. 4-Amino-6-benzyl-2-methylpyridazin-3(2H)-one (**38d**)

Yield = 48%; mp = 104–108 °C (EtOH). ^1^H NMR (400 MHz, CDCl_3_) δ 3.80 (s, 3H, N-CH_3_), 3.82 (s, 2H, CH_2_Ph), 4.81 (exch br s, 2H, NH_2_), 6.07 (s, 1H, -CH pyridaz.), 7.22–7.35 (m, 5H, Ar). Anal. Calcd for C_12_H_13_N_3_O: C, 66.96; H, 6.09; N, 19.52. Found C, 66.83; H, 5.50; N, 20.83.

#### 3.2.42. General Procedure for Compounds **39a,b, 40** and **43**

Compounds **39a,b, 40** and **43** were obtained starting from **38b,c, 37e** and **42a**, respectively, through the same procedure described for compound **25a–f**.

#### 3.2.43. (6-Cyclohexyl-2-methyl-3-oxo-2,3-dihydropyridazin-4-yl)urea (**39a**)

Yield = 66%; mp = 251–254 °C (EtOH). ^1^H NMR (400 MHz, DMSO-d_6_) δ 1.30–1.40 (m, 5H, C_6_H_11_), 1.70–1.85 (m, 5H, C_6_H_11_), 2.45 (m, 1H, C_6_H_11_), 3.64 (s, 3H, N-CH_3_), 6.74 (exch br s, 2H, CONH_2_), 7.79 (s, 1H, -CH pyridaz.), 8.84 (exch br s, 1H, NHCO). Anal. Calcd for C_12_H_18_N_4_O_2_: C, 57.58; H, 7.25; N, 22.38. Found C, 57.41; H, 7.23; N, 22.43.

#### 3.2.44. (6-Isopropyl-2-methyl-3-oxo-2,3-dihydropyridazin-4-yl)urea (**39b**)

Yield = 60%; mp = 248–251 °C (EtOH). ^1^H NMR (400 MHz, DMSO-d_6_) δ 1.15 (d, 6H, CH(CH_3_)_2_, J = 6.8 Hz), 2.80 (m, 1H, CH(CH_3_)_2_), 3.65 (s, 3H, N-CH_3_), 6.74 (exch br s, 2H, CONH_2_), 7.81 (s, 1H, -CH pyridaz.), 8.85 (exch br s, 1H, CONH). Anal. Calcd for C_9_H_14_N_4_O_2_: C, 51.42; H, 6.71; N, 26.65. Found C, 51.31; H, 6,70; N, 26.61.

#### 3.2.45. [6-(2-Hydroxy-phenyl)-3-oxo-2,3-dihydro-pyridazin-4-yl]-urea (**40)**

Yield = 85%; mp > 300 °C (EtOH). ^1^H NMR (400 MHz, DMSO-d_6_) δ 6.75 (exch br s, 2H, CONH_2_), 6.90–6.95 (m, 2H, Ar), 7.27 (t, 1H, Ar, J = 8.4 Hz), 7.45 (dd, 1H, Ar, J_1_ = 1.2 Hz and J_2_ = 8.0 Hz), 8.39 (s, 1H, -CH pyridaz.), 8.92 (exch br s, 1H, NHCO), 10.43 (exch br s, 1H, OH), 13.18 (exch br s, 1H, NH). Anal. Calcd for C_11_H_10_N_4_O_3_: C, 53.66; H, 4.09; N, 22.75. Found C, 53.51; H, 4,08; N, 22.81.

#### 3.2.46. [6-(2-Hydroxyphenyl)-2-methyl-3-oxo-2,3-dihydropyridazin-4-yl]-urea (**43**)

Yield = 95%; mp = 278–280 °C (EtOH). ^1^H-NMR (400 MHz, DMSO-d_6_) δ 3.76 (s, 3H, N-CH_3_), 6.79 (exch br s, 2H, NH_2_), 6.88–6.95 (m, 2H, Ar), 7.27 (t, 1H, Ar, J = 7.2 Hz), 7.44 (d, 1H, Ar, J = 6.8 Hz), 8.37 (s, 1H, -CH pyridaz.), 8.93 (exch br s, 1H, NHCO), 10.17 (exch br s, 1H, OH). Anal. Calcd for C_12_H_12_N_4_O_3_: C, 55.38; H, 4.65; N, 21.53. Found C, 55.49; H, 4,65; N, 21,49.

#### 3.2.47. N-[4-(5-Amino-1-methyl-6-oxo-1,6-dihydropyridazin-3-yl)-phenyl]-acetamide (**44**)

To a cooled (0 °C) and stirred solution of **42b** (0.93 mmol) in anhydrous THF (2–3 mL), 1.02 mmol of acetyl chloride was added and the mixture was stirred at room temperature for 20 min. After dilution with cold water (20–30 mL), the precipitate was recovered by filtration under vacuum and purified by crystallization. Yield = 92%; mp = 270–272 °C (Cyclohexane). ^1^H NMR (400 MHz, DMSO-d_6_) δ 2.06 (s, 3H, COCH_3_), 3.69 (s, 3H, N-CH_3_), 6.48 (exch br s, 2H, NH_2_), 6.71 (s, 1H, -CH pyridaz.), 7.60–7.70 (m, 4H, Ar), 8.80 (exch br s, 1H, NHCO). Anal. Calcd for C_13_H_14_N_4_O_2_: C, 60.45; H, 5.46; N, 21.69. Found C, 60.58; H, 5.45; N, 21.63.

#### 3.2.48. 3,6-Dimethyl-4-pyridin-2-yl-isoxazolo [3,4-d]pyridazin-7(6H)-one (**46**)

To a cooled (0–4 °C) solution of **45** [34] (0.38 mmol) in EtOH (2–3 mL), methylhydrazine (1.30 mmol) was added and the mixture was stirred at room temperature for 90 min. The precipitate was recovered by filtration under vacuum to obtain the desired compound. Yield = 84%; mp = 154–155 °C (EtOH). ^1^H NMR (400 MHz, DMSO-d_6_) δ 2.94 (s, 3H, C3-CH_3_), 3.74 (s, 3H, N-CH_3_), 7.57 (t, 1H, Ar, J = 5.2 Hz), 7.95–8.05 (m, 2H, Ar), 8.76 (d, 1H, Ar, J = 5.2 Hz). Anal. Calcd for C_12_H_10_N_4_O_2_: C, 59.50; H, 4.16; N, 23.13. Found C, 59.66; H, 4.16; N, 23.20.

#### 3.2.49. 5-Acetyl-4-amino-2-methyl-6-pyridin-2-yl-pyridazin-3(2H)-one (**47**)

A mixture of **46** (0.82 mmol), 10% Pd/C (20 mg) and ammonium formate (4.9 mmol) in EtOH (5 mL), was refluxed for 2 h. After addition of CH_2_Cl_2_ (4–5 mL) and filtration of charcoal, evaporation of the solvent afforded the product **47**. Yield = 65%; mp = 201–203 °C (EtOH). ^1^H NMR (400 MHz, DMSO-d_6_) δ 1.89 (s, 3H, COCH_3_), 3.73 (s, 3H, N-CH_3_), 7.23 (exch br s, 2H, NH_2_), 7.44–7.50 (m, 1H, Ar), 7.89 (d, 1H, Ar, J = 7.2 Hz), 7.96 (t, 1H, Ar, J = 7.2 Hz), 8.57 (d, 1H, Ar, J = 4.4 Hz). Anal. Calcd for C_12_H_12_N_4_O_2_: C, 59.01; H, 4.95; N, 22.94. Found C, 59.18; H, 4.96; N, 22.99.

#### 3.2.50. 4-Amino-2-methyl-6-pyridin-2-yl-pyridazin-3(2H)-one (**48**)

A suspension of **47** (0.53 mmol) in 1 mL of 48% HBr was stirred in a sealed/pressure vessel at 130 °C for 3 h. After cooling ice-cold water was added and the precipitate was recovered by filtration under vacuum to obtain the desired product 48. Yield = 65%; mp = 294–295 °C (EtOH). ^1^H NMR (400 MHz, DMSO-d_6_) δ 3.75 (s, 3H, N-CH_3_), 7.24 (s, 1H, -CH pyridaz.), 7.51 (m, 1H, Ar), 8.00 (t, 1H, Ar, J = 7.2 Hz), 8.12 (d, 1H, Ar, J = 8.0 Hz), 8.66 (d, 1H, Ar, J = 4.4 Hz). Anal. Calcd for C_10_H_10_N_4_O: C, 59.40; H, 4.98; N, 27.71. Found C, 59.51; H, 4.99; N, 27.75.

#### 3.2.51. (2-Methyl-3-oxo-6-pyridin-2-yl-2,3-dihydropyridazin-4-yl)-urea (**49**)

Compound **49** was obtained starting from **48**, through the same procedure described for compounds **25a–f**, **39a,b**, **40** and **43.** Yield = 15%; mp > 300 °C (EtOH). ^1^H NMR (400 MHz, DMSO-d_6_) δ 3.82 (s, 3H, N-CH_3_), 6.80 (exch br s, 2H, NH_2_), 7.46 (m, 1H, Ar), 7.92 (t, 1H, Ar, J = 7.6 Hz), 8.10 (d, 1H, Ar, J = 7.6 Hz), 8.67 (d, 1H, Ar, J = 4.8 Hz), 8.83 (s, 1H, -CH pyridaz.), 8.97 (exch br s, 1H, NHCO). Anal. Calcd for C_11_H_11_N_5_O_2_: C, 53.87; H, 4.52; N, 28.56. Found C, 53.78; H, 5.00; N, 27.71.

#### 3.2.52. 2-Methyl-3-oxo-6-phenyl-2,3-dihydropyridazine-4-carboxamide (**51**)

Compound **51** was obtained starting from **50** [41], through the same procedure described for compounds **38a–d**. The compound was purified by crystallization from diethyl ether. Yield = 55%; mp = 215–217 °C (Et_2_O). Light brown solid, ^1^H NMR (400 MHz, CDCl_3_) δ 3.99 (s, 3H, CH_3_), 5.98 (exch br s, 1H, CONH_2_), 7.43–7.51 (m, 3H, Ar), 7.84–7.88 (m, 2H, Ar), 8.72 (s, 1H, Ar), 9.41 (exch br s, 1H, CONH_2_). ^13^C NMR (100 MHz, CDCl_3_) δ 163.58, 160.21, 145.33, 134.16, 132.64, 130.02, 129.23, 129.04, 126.15, 41.53. MS-ESI for C_12_H_11_N_3_O_2_ (Calcd, 229.08), [M + H]^+^ at *m/z* 229.90, t_R_ = 12.205. Anal. Calcd for C_12_H_11_N_3_O_2_: C, 62.87; H, 4.84; N, 18.33. Found C, 62.62; H, 4.82; N, 18.26.

#### 3.2.53. 1-(6-Methyl-3-oxo-2-phenyl-2,3-dihydropyridazin-4-yl)urea (**55**)

Compound **55** was obtained strating from **54** [43], through the same procedure for the formation of urea described for compounds **25a–f, 39a,b, 40** and **43**. Yield = 95%; mp = 288–290 °C (EtOH). White coloured solid, ^1^H NMR (400 MHz, DMSO-d_6_) δ 2.25 (s, 3H, CH_3_), 7.40–7.42 (m, 1H, Ar), 7.47 (d, 2H, J = 8.2 Hz, Ar), 7.50–7.54 (m, 2H, Ar), 7.78 (s, 1H, Ar), 8.91 (s, 1H, NHCONH_2_). ^13^C NMR (100 MHz, DMSO-d_6_) δ 155.56, 154.89, 146.52, 141.98, 138.04, 128.86, 128.12, 125.97, 109.55, 21.48. MS-ESI for C_12_H_12_N_4_O_2_ (Calcd, 244.10), [M + H]^+^ at *m/z* 244.88, t_R_ = 10.100. Anal. Calcd for C_12_H_12_N_4_O_2_: C, 59.01; H, 4.95; N, 22.94. Found C, 59.25; H, 4.97; N, 23.03.

#### 3.2.54. (E)-3-(2-(Dimethylamino)vinyl)-4-methyl-6-phenylisoxazolo [3,4-d]pyridazin-7(6H)-one (**56**)

A mixture of **52** (1.04 mmol) [42] in 2.5 mL of DMF-DMA was hetaed at 90–100 °C for 1 h. After cooling, ice/cold water was added (15 mL) and the precipitate obtained was recovered by filtration under vacuum to obtained the pure desired compound. Yield = 90%; mp = 224–226 °C dec. (Cyclohexane). ^1^H NMR (400 MHz, CDCl_3_) δ 2.50 (s, 3H, CH_3_), 3.00–3.20 (m, 6H, N(CH_3_)_2_), 5.25 (d, 1H, CH=CH-N, J = 10.0 Hz), 7.30–7.35 (m, 1H, Ar), 7.45–7.50 (m, 2H, Ar), 7.59–7.64 (m, 3H, 1H CH=CH-N + 2H Ar). Anal. Calcd for C_16_H_16_N_4_O_2_: C, 64.85; H, 5.44; N, 18.91. Found C, 65.10; H, 5.46; N, 18.98.

#### 3.2.55. 4-Amino-6-methyl-2-phenyl-5-(1H-pyrazol-5-yl)pyridazin-3(2H)-one (**57**)

A mixture of intermediate **56** (0.81 mmol) and 1 mL of hydrazine hydrate (excess) in 2 mL of abs. EtOH was hetaed at 70 °C for 10 h. After cooling, ice/cold water was added (15 mL). The precipitate obtained was recovered by filtration under vacum and purified by crystallization from ethanol. Yield = 65%; mp = 119–121 °C. (Cyclohexane). Yellow coloured solid, ^1^H NMR (400 MHz, Methanol-d_4_) δ 2.31 (s, 3H, CH_3_), 6.58 (exch br s, 2H, NH_2_), 7.43 (t, 1H, J = 7.3 Hz, Ar), 7.52 (t, 3H, J = 7.6 Hz, Ar), 7.58 (d, 2H, J = 7.6 Hz, Ar), 7.83 (s, 1H, NH). ^13^C NMR (100 MHz, Methanol-d_4_) δ 174.64, 147.92, 143.30, 129.88, 129.32, 127.14, 106.95, 24.30. MS-ESI for C_14_H_13_N_5_O (Calcd, 267.11), [M + H]^+^ at *m/z* 267.98. t_R_ = 10.882. Anal. Calcd for C_14_H_13_N_5_O: C, 62.91; H, 4.90; N, 26.20. Found C, 62.66; H, 4.88; N, 26.09.

### 3.3. Molecular Modeling and Biological Data

The 2D chemical structures were built using Marvin Sketch and all the structures were subjected to molecular mechanics energy minimization using the MMFF94 force field present in the same software [45]. The 3D geometry of all compounds was then optimized using the PM3 Hamiltonian [46], as implemented in MOPAC 2016 package assuming a pH of 7.0 [47]. Once built and optimized, all structures were used in the bioisostere replacement tool Spark 10.4.0. Five hundred compounds were generated for the substitution (50 best compounds reported in the Supplementary Materials). The isosteric replacement was performed using the same 178,558 fragments for each part; in particular, the fragments derive from ChEMBL and Zinc databases with a protocol already reported and validated [27,48,49]. Ligand growing experiments were performed in the selected pyridazinone structure using an already reported protocol [50]. Docking calculations were made using AutoDock with the default docking parameters and a validated protocol [51,52]. The setup was done with YASARA [47]. The Lamarckian genetic algorithm implemented in AutoDock was used for the calculations. The ligand-centered maps were generated by AutoGrid with a spacing of 0.375 Å and dimensions that encompass all atoms extending 5 Å from the surface of the ligand. All of the parameters were inserted at their default settings. The X-ray crystal structures of the co-crystal FABP4/(2-[(2-oxo-2-piperidin-1-ylethyl)sulfanyl]-6-(trifluoromethyl)pyrimidin-4-ol) (PDBid: 1TOU) was downloaded from the Protein Data Bank (www.rcsb.org accessed on 15 June 2022).

### 3.4. FABP Inhibitory Activity Assays

To analyze the inhibitory activity of FABP4 ligands, a displacement assay was utilized as described by the Cayman’s instruction, FABP4 Inhibitor/Ligand Screening Assay Kit, Item 10,010,231 (see Supplementary Materials for additional details). The samples of compounds for activity determination were prepared as a stock solution (1 mM) in DMSO. On the day of activity assay, the compounds were all diluted in phosphate buffer solution (PBS, pH 7.4) to different concentrations (100, 50, 10, 5, 2, 1, and 0 µM). Appropriate concentrations of DMSO in PBS were used as control. The detection reagent (FABP Assay Detection Reagent, Item 10010376) was used as provided by the Cayman’s kit. The diluted Detection Reagent probe was mixed with FABP4 protein present in the kit and incubated for 10 min at room temperature. Compounds were then added and equilibrated for another 10 min. Lastly, the fluorescence signal was recorded at 470 nm (i.e., emission, with the excitation fixed at 370 nm) with a CytoFluor^®^ Series 4000 Fluorescence Multi-Well Plate Reader. The IC_50_ was calculated as indicated in the kit booklet of FABP4 Inhibitor/Ligand Screening Assay Kit (Item No. 10010231) Cayman chemicals, as follows: 1) calculate the average fluorescence of each sample; 2) calculate the background corrected fluorescence (BCF) by subtracting the blank; 3) divide the BCF of each sample by the maximum BCF and multiply by 100% (this is the value in percent fluorescence units, i.e., % FU); 4) plot the % FU values against the concentration of inhibitor/ligand used; 5) find the concentration of inhibitor/ligand that corresponds to 50% FU, to determine IC_50_ values.

## 4. Conclusions

We have identified novel 4-amino and 4-ureido pyridazinone-based FABP4 inhibitors whose design was directed by computing assisted molecular design of bioisosteric-replacements/scaffold hopping of the pyrimidine skeleton of the co-crystallyzed ligand 1TOU. Selected compounds have been synthesized and tested for their ability to inhibit FABP4. Among the new series, ten compounds were further evaluated on the basis of their inhibitory activity on FABP4 established via a single point displacement assay. In particular, **4b**, **25a**, **30b** and **22** exhibited high FABP4 inhibitory activity with IC_50_ in the low micromolar range. The results demonstrated that compound **25a** was the most potent analogue in terms of displacement of the arachidonic acid, with an IC_50_ value of 2.97 μM, which is lower than the IC_50_ of the positive control (3.42 µM). Docking experiments, conducted with the most active compounds **4b**, **25a**, **30b**, **22**, confirmed the ability of these molecules to interact with several amino acid residues present inside the FABP4 binding pocket, with the stronger interaction exhibited by compound **25a**. This result is in agreement with the higher activity recorded in vitro for **25a**, in comparison to the other 4-amino and 4-ureido pyridazinone-based analogues developed in this study.

## Figures and Tables

**Table 1:** 4-NH_2_-pyridazinones synthesized and screened against FABP4.

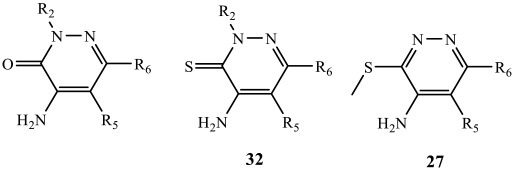			
Comp.	R_2_	R_5_	R_6_
**4a**	H	CONHPh	Ph
**4b**	H	CONH*n*C_3_H_7_	Ph
**5a**	C_2_H_5_	CONHPh	Ph
**5b**	C_2_H_5_	CONH*n*C_3_H_7_	Ph
**6**	H	CONH_2_	Ph
**7**	H	CN	Ph
**16**	Ph	CONH_2_	CH_3_
**17**	Ph	CN	CH_3_
**18**	Ph	COCH_3_	H
**21**	cC_6_H_11_	CONH_2_	Ph
**22**	cC_6_H_11_	CN	Ph
**24d**	*i*C_3_H_7_	H	Ph
**24e**	*n*C_3_H_7_	H	Ph
**24f**	*n*C_4_H_9_	H	Ph
**27**	-	H	Ph
**32**	CH_3_	H	Ph
**37a**	H	H	3-thienyl
**37c**	H	H	*c*C_6_H_11_
**37d**	H	H	*i*C_3_H_7_
**38a**	CH_3_	H	3-thienyl
**38b**	CH_3_	H	*c*C_6_H_11_
**38c**	CH_3_	H	*i*C_3_H_7_
**38d**	CH_3_	H	CH_2_-Ph
**42a**	CH_3_	H	2-(OH)-Ph
**42b**	CH_3_	H	4-(NH_2_)-Ph
**44**	CH_3_	H	4-(NHCOCH_3_)-Ph
**48**	CH_3_	H	2-pyridinyl
**54**	Ph	H	CH_3_
**57**	Ph	pyrazole	CH_3_

**Table 2:** 4-Amino and 4-ureido pyridazinones synthesized and screened against FABP4.

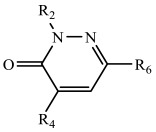			
Comp.	R_2_	R_4_	R_6_
**25a**	CH_3_	NHCONH_2_	Ph
**25b**	*c*C_6_H_11_	NHCONH_2_	Ph
**25c**	C_2_H_5_	NHCONH_2_	Ph
**25d**	*i*C_3_H_7_	NHCONH_2_	Ph
**25e**	*n*C_3_H_7_	NHCONH_2_	Ph
**25f**	*n*C_4_H_9_	NHCONH_2_	Ph
**28**	H	NHCONH_2_	Ph
**29a**	CH_3_	NHCOCH_3_	Ph
**29b**	CH_3_	NHCOC_2_H_5_	Ph
**29c**	CH_3_	NHCO*i*C_3_H_7_	Ph
**29d**	CH_3_	NHCOnC_3_H_7_	Ph
**30a**	CH_3_	NH-(3-CN)-Ph	Ph
**30b**	CH_3_	NH-(2-CN)-Ph	Ph
**31a**	CH_3_	NH-(3-CONH_2_)-Ph	Ph
**31b**	CH_3_	NH-(2-CONH_2_)-Ph	Ph
**35**	CH_3_	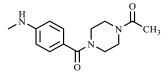	Ph
**39a**	CH_3_	NHCONH_2_	*c*C_6_H_11_
**39b**	CH_3_	NHCONH_2_	*i*C_3_H_7_
**40**	H	NHCONH_2_	2-(OH)-Ph
**43**	CH_3_	NHCONH_2_	2-(OH)-Ph
**49**	CH_3_	NHCONH_2_	2-pyridinyl
**51**	CH_3_	CONH_2_	Ph
**55**	Ph	NHCONH_2_	CH_3_

**Table 3:** Measured IC_50_ values for selected compounds.

Compounds	IC_50_ (µM)
**Arachidonic acid**	3.42 ± 0.54
**4b**	8.27 ± 0.20
**25a**	2.97 ± 0.26
**30b**	23.18 ± 0.52
**22**	15.23 ± 0.76
**25c**	>50
**35**	>50
**25e**	>50
**54**	>50
**55**	>50
**27**	>50

## Data Availability

Data is contained within the article or supplementary material.

## Supplementary Materials

The following supporting information can be downloaded at: https://www.mdpi.com/article/10.3390/ph15111335/s1, ^1^H NMRs of selected compounds; ^13^C NMRs of selected compounds; Mass spectra of selected compounds; HPLC/UV chromatograms of selected compounds; 50 ‘best-fit’ compounds generated with scaffold hopping replacement; Info on the FABP4 inhibitor assay kit; Averaged data as Background corrected fluorescence for IC_50_ measured compounds.
