# Supplementary material for: Ligand Growing Experiments Suggested 4-amino and 4-ureido pyridazin-3(2H)-one as Novel Scaffold for FABP4 Inhibition
Source: Pharmaceuticals (Basel). 2022 Oct 28;15(11):1335. doi: 10.3390/ph15111335 (PMC9697826; doi:10.3390/ph15111335)
Supplement: Supplementary file 1 [file pharmaceuticals-15-01335-s001.zip › pharmaceuticals-1983713-supplementary.pdf]

Supporting Information

For

Ligand growing experiments suggested 4-amino and 4-ureido pyridazin-3(2*H*)-one as novel scaffold for FABP4 inhibition

Letizia Crocetti [1,*], Giuseppe Floresta [2], Chiara Zagni [2], Divya Merugu [3], Francesca Mazzacuva [4], Renan Rodrigues de Oliveira Silva [3], Claudia Vergelli [2], Maria Paola Giovannoni [2], and Agostino Cilibrizzi [3,5,*]

[1]Dipartimento NEUROFARBA – Pharmaceutical and Nutraceutical Section, via Ugo Schiff 6, 50019 Sesto Fiorentino (FI), Italia.
[2]Dipartimento di Scienze del Farmaco e della Salute, Università di Catania, Viale A. Doria 6, 95125 Catania, Italy
[3]Institute of Pharmaceutical Science, King's College London, Stamford Street, London, SE1 9NH, UK
[4]School of Health, Sport and Bioscience, University of East London, London, E15 4LZ, UK
[5]Centre for Therapeutic Innovation, University of Bath, Bath, UK

*****Correspondence: L.C., letizia.crocetti@unifi.it, Tel. +39 0554573683; A.C., agostino.cilibrizzi@kcl.ac.uk, +44 (0) 20 7848 9532.

Table of content

1. ^{1}H NMRs of selected compounds S2
2. ^{13}C NMRs of selected compounds S15
3. Mass spectra of selected compounds S27
4. HPLC/UV chromatograms of selected compounds S40
5. 50 'best-fit' compounds generated with scaffold hopping replacement S50

^{1}H- NMR

4a

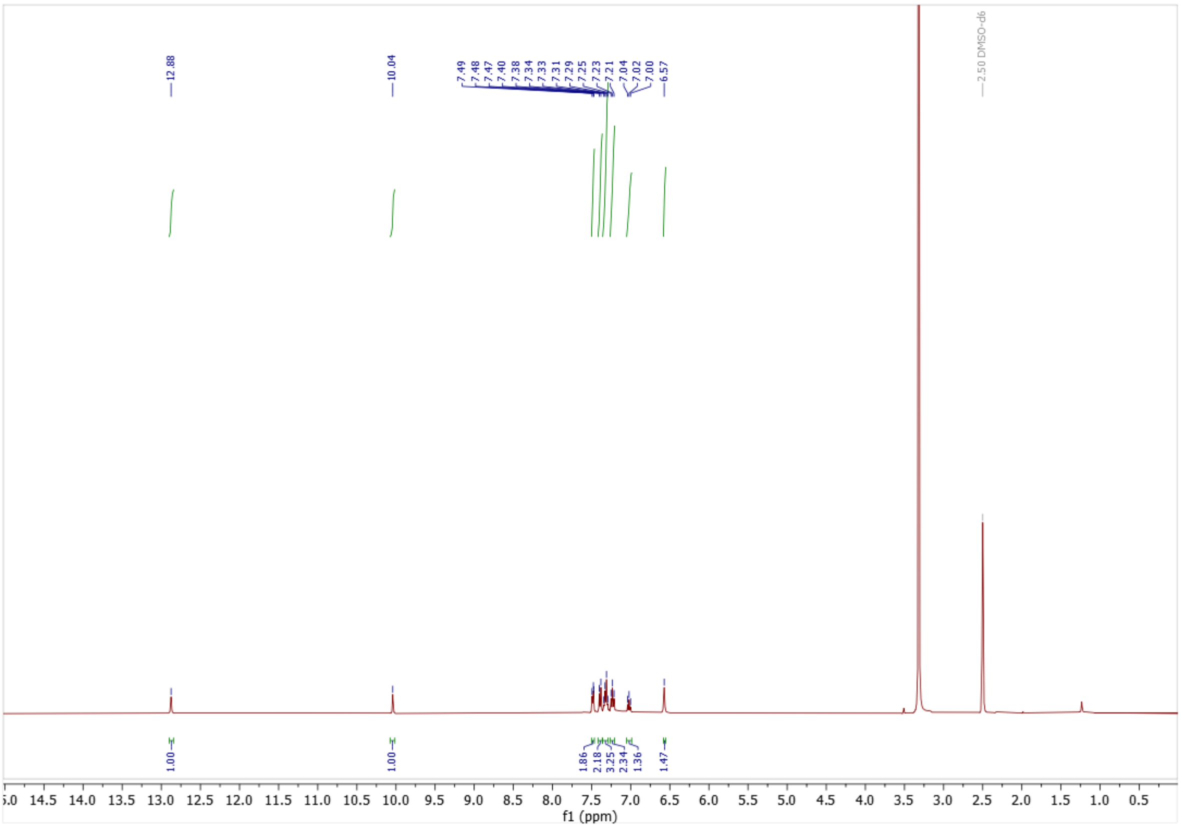
12.88
10.04
7.49
7.48
7.47
7.40
7.38
7.34
7.33
7.31
7.29
7.25
7.23
7.21
7.04
7.02
7.00
6.57
2.50 DMSO-d6
1.00
1.00
1.86
2.18
3.25
2.34
1.36
1.47
f1 (ppm)

4b

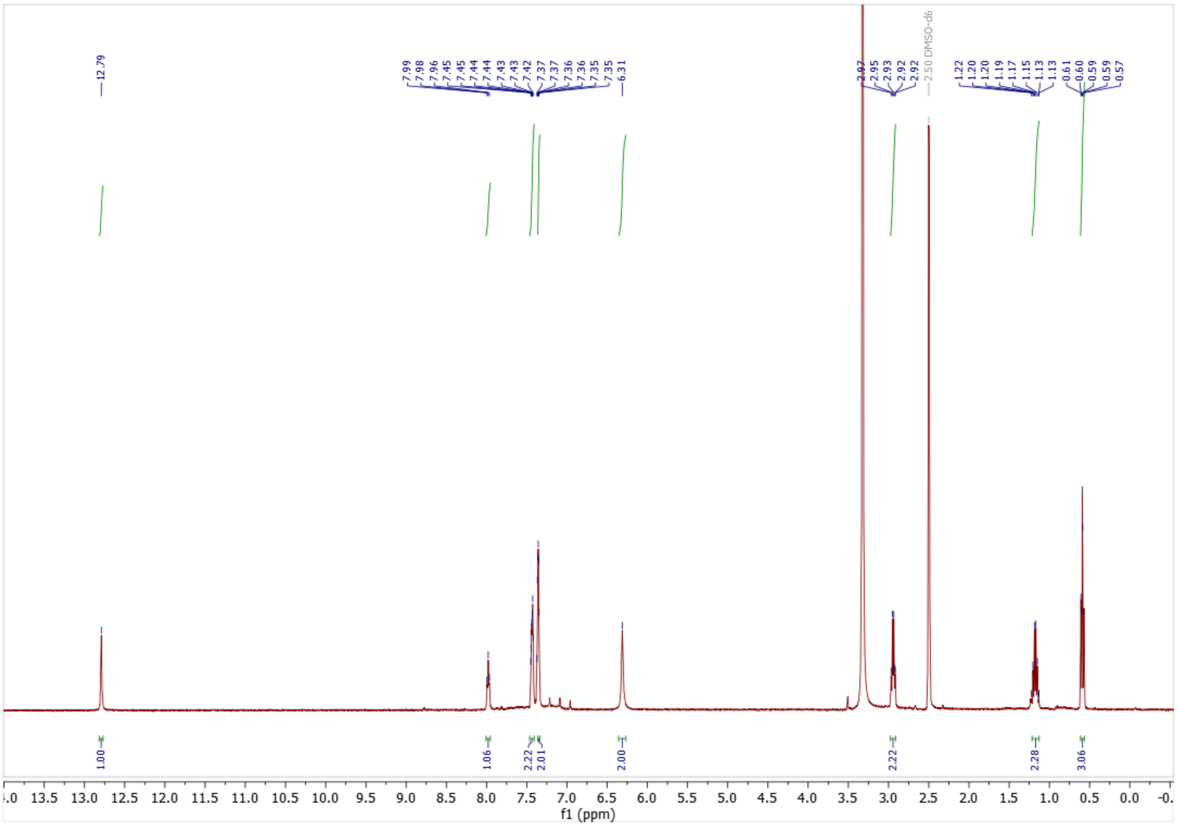
12.79
7.99
7.98
7.96
7.45
7.45
7.44
7.44
7.43
7.43
7.42
7.37
7.37
7.36
7.36
7.35
7.35
6.31
2.97
2.95
2.93
2.92
2.92
2.50 DMSO-d6
1.22
1.20
1.20
1.19
1.17
1.15
1.13
1.13
0.61
0.60
0.59
0.59
0.57
1.00
1.06
2.22
2.01
2.00
2.22
2.28
3.06
f1 (ppm)

6

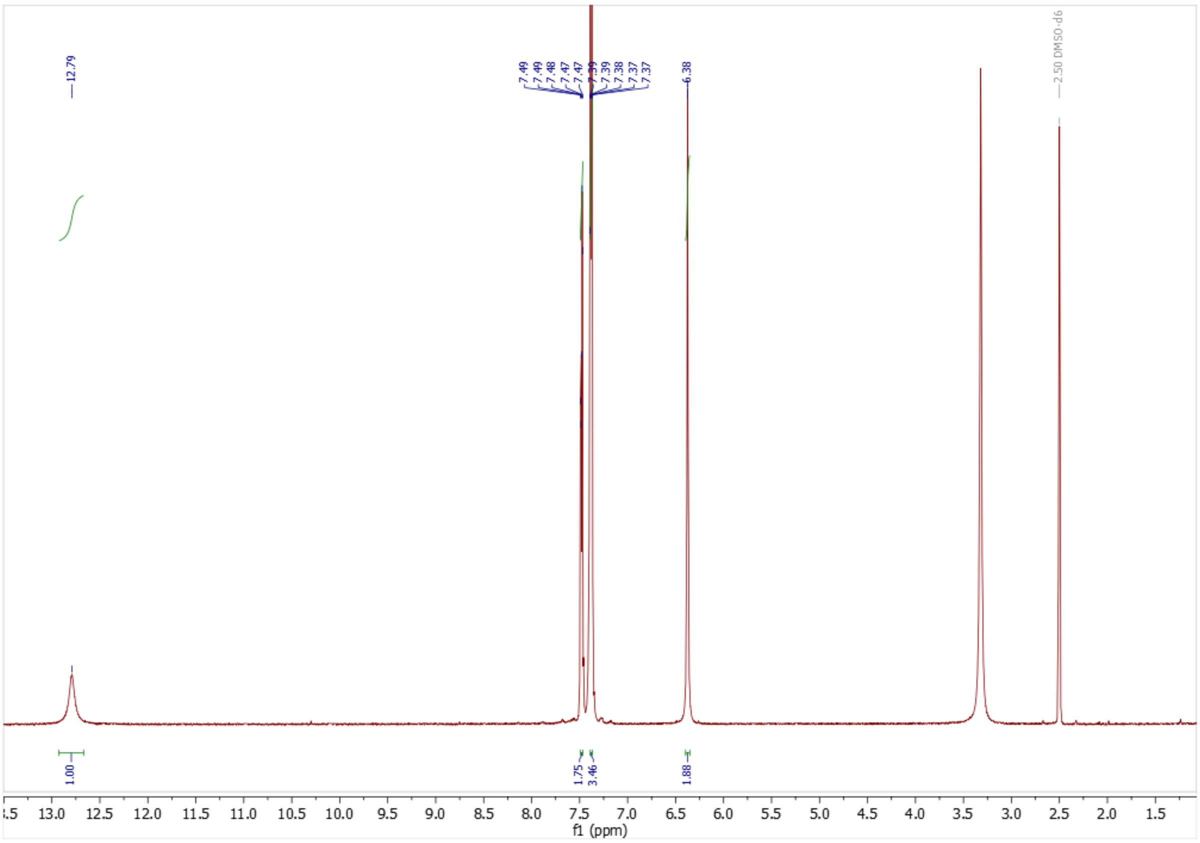
12.79
7.49
7.49
7.48
7.47
7.47
7.39
7.39
7.38
7.37
7.37
6.38
2.50 DMSO-d6
1.00
1.75
3.46
1.88
f1 (ppm)

7

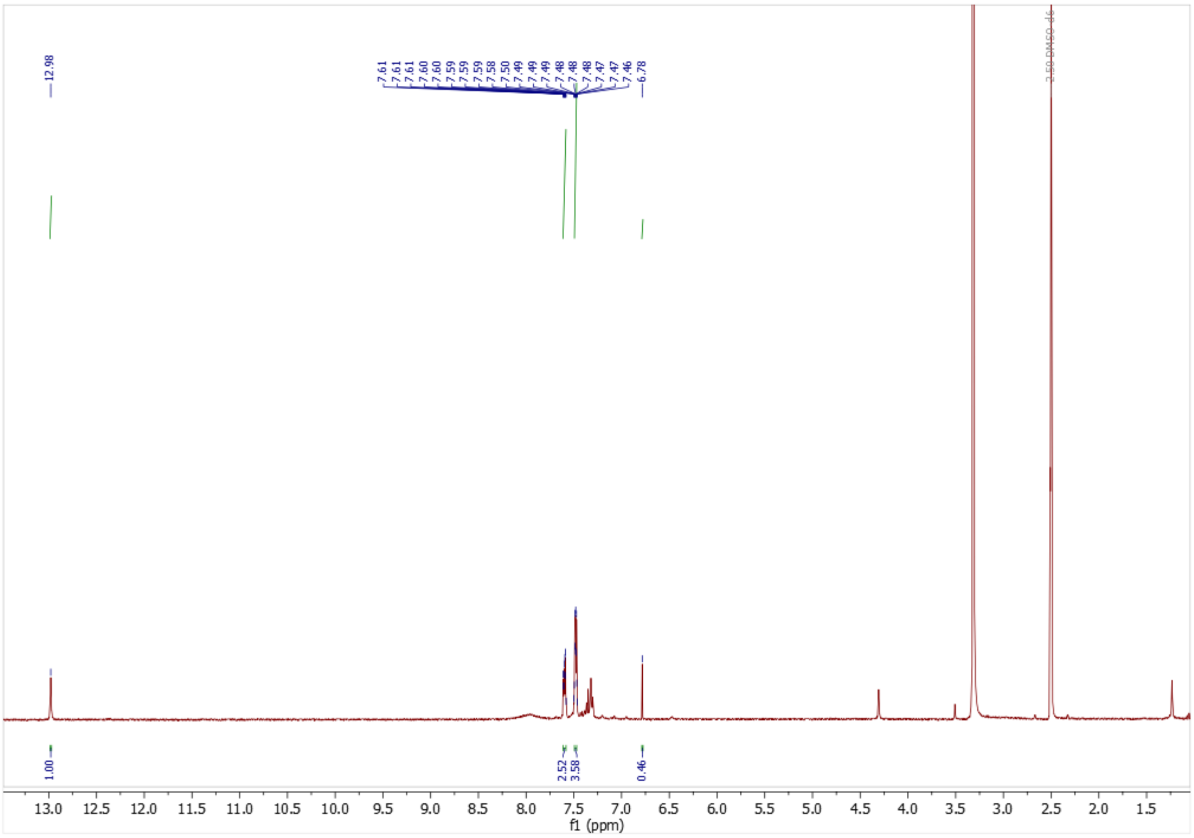
12.98
7.61
7.61
7.61
7.60
7.60
7.59
7.59
7.59
7.58
7.50
7.49
7.49
7.49
7.48
7.48
7.48
7.48
7.47
7.47
7.46
6.78
1.00
2.52
3.58
0.46
f1 (ppm)

16

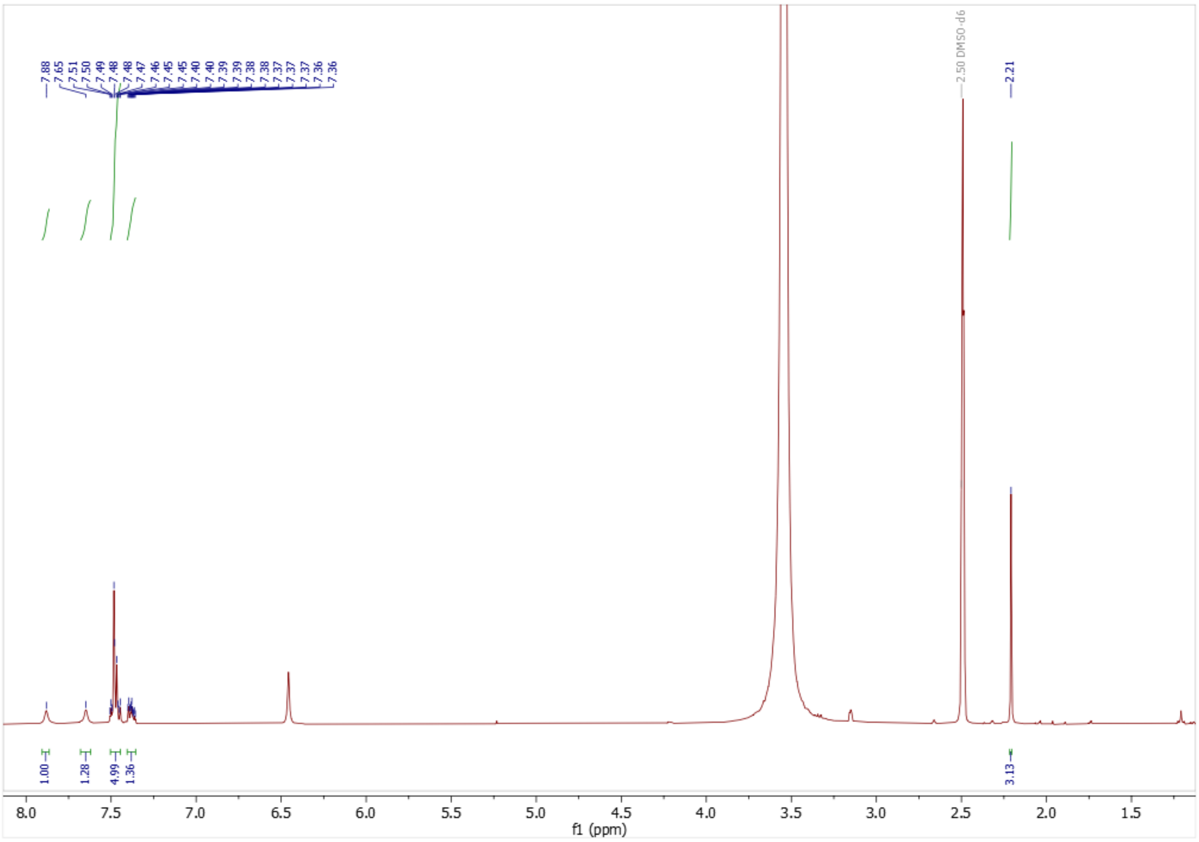

17

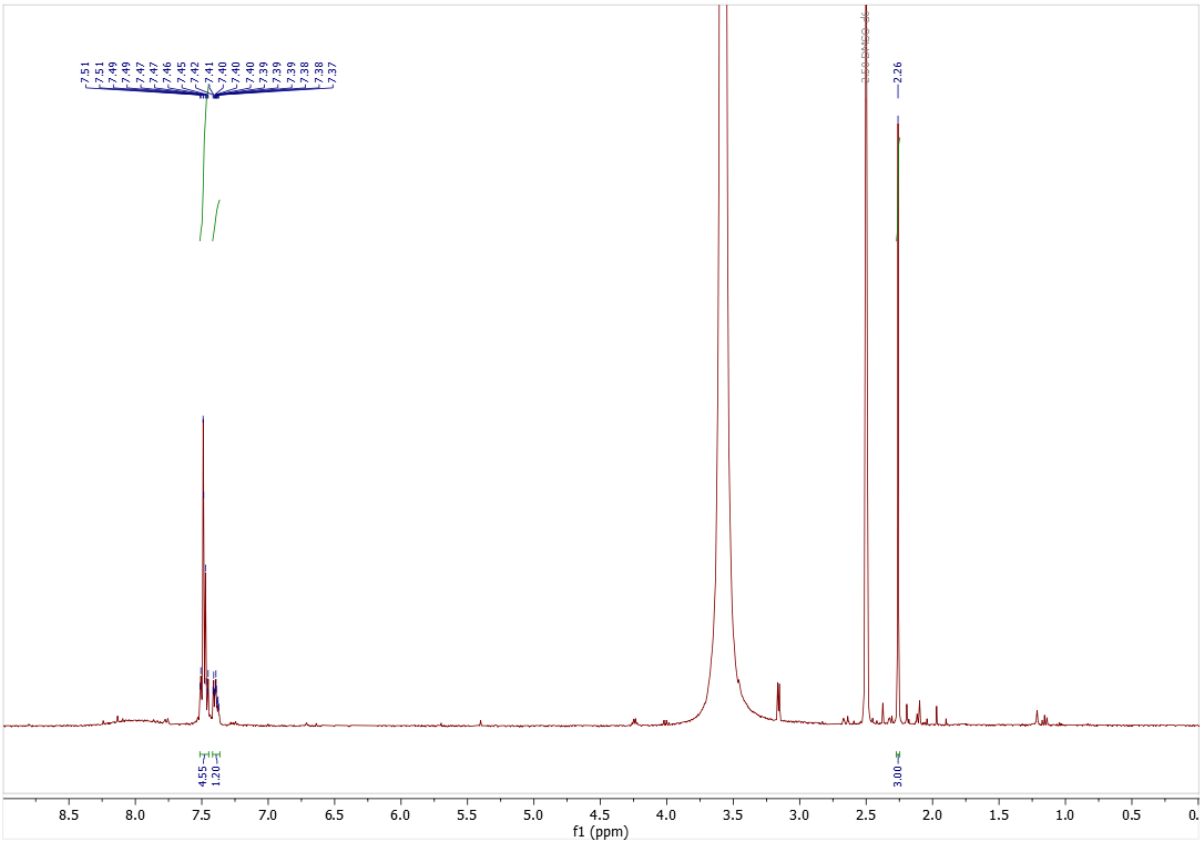

22

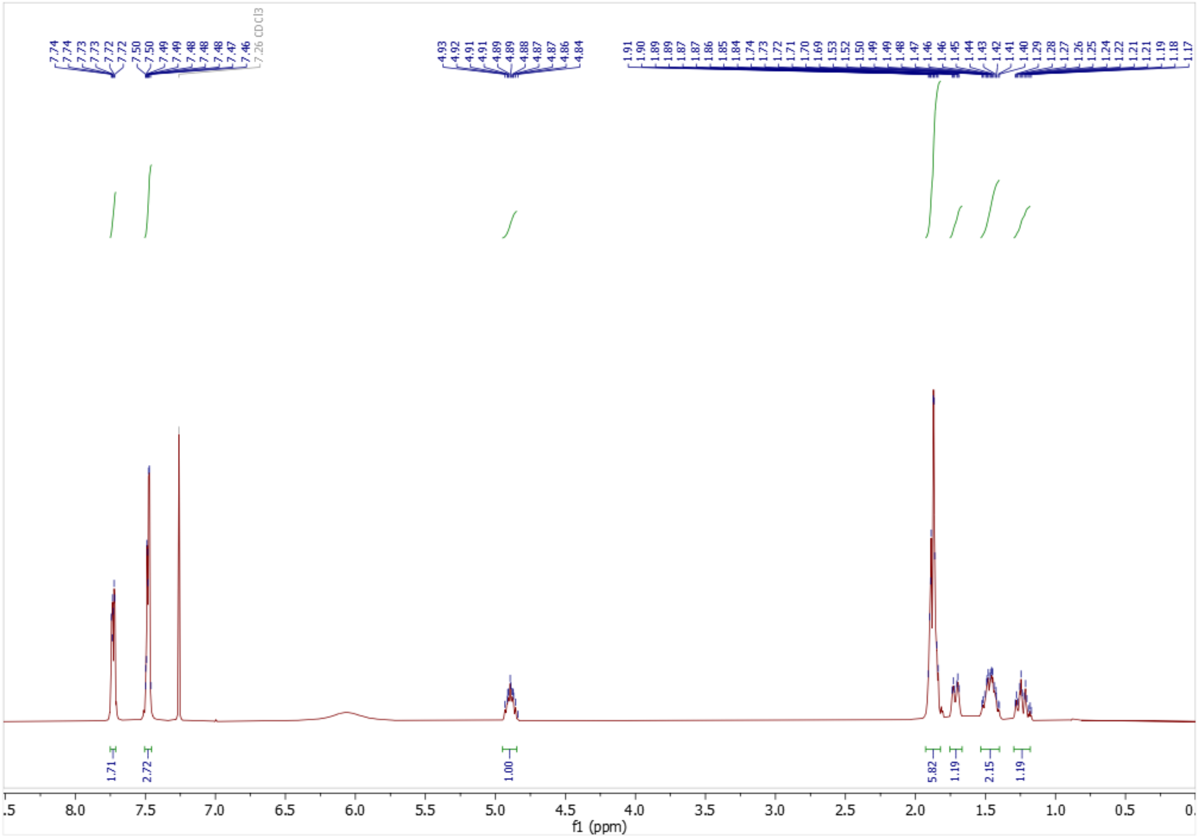

25a (EtOAc crossed)

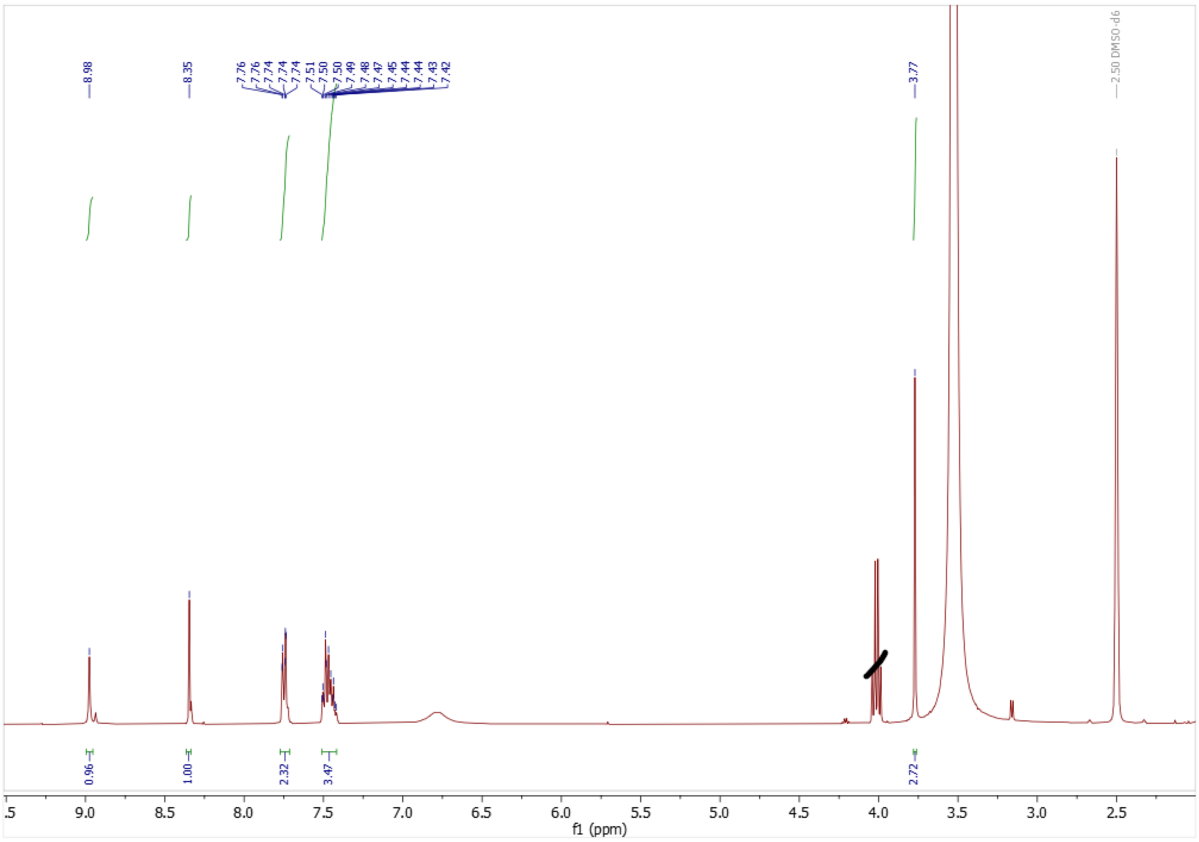

25c

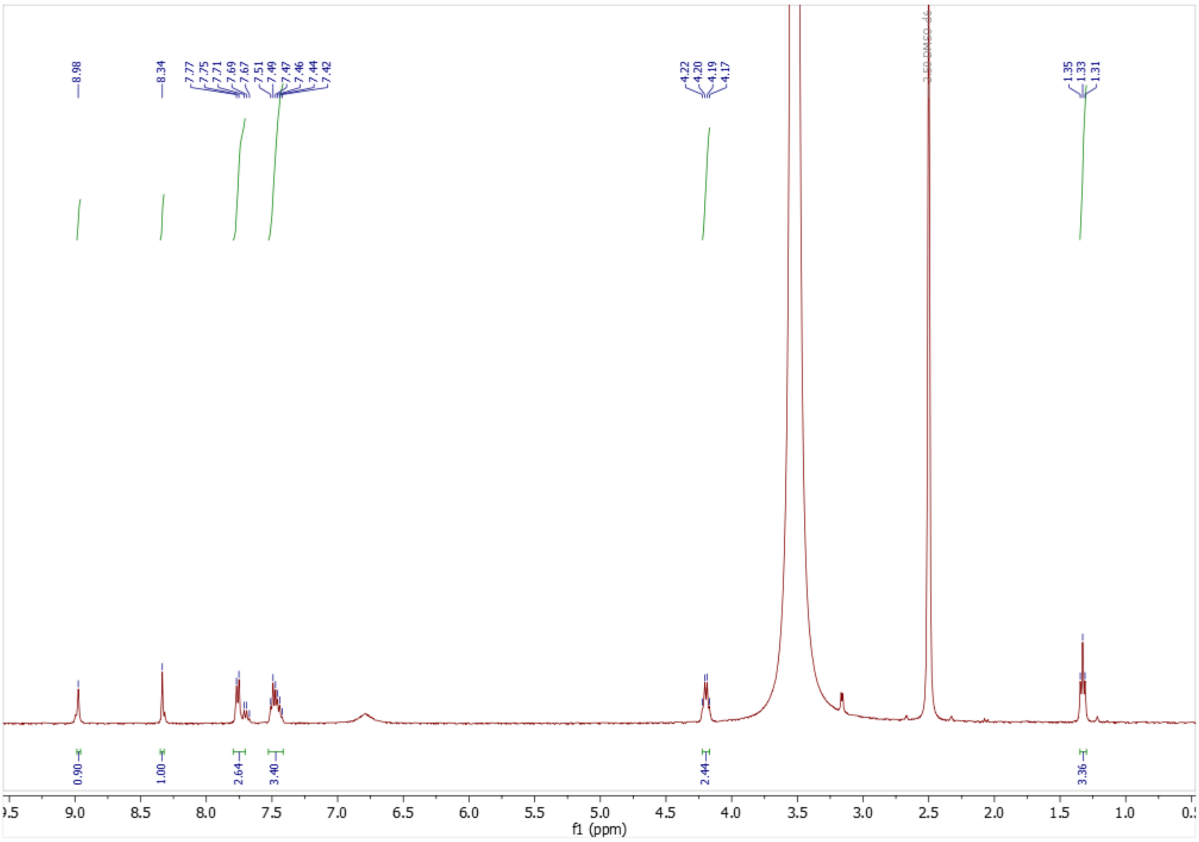

25d

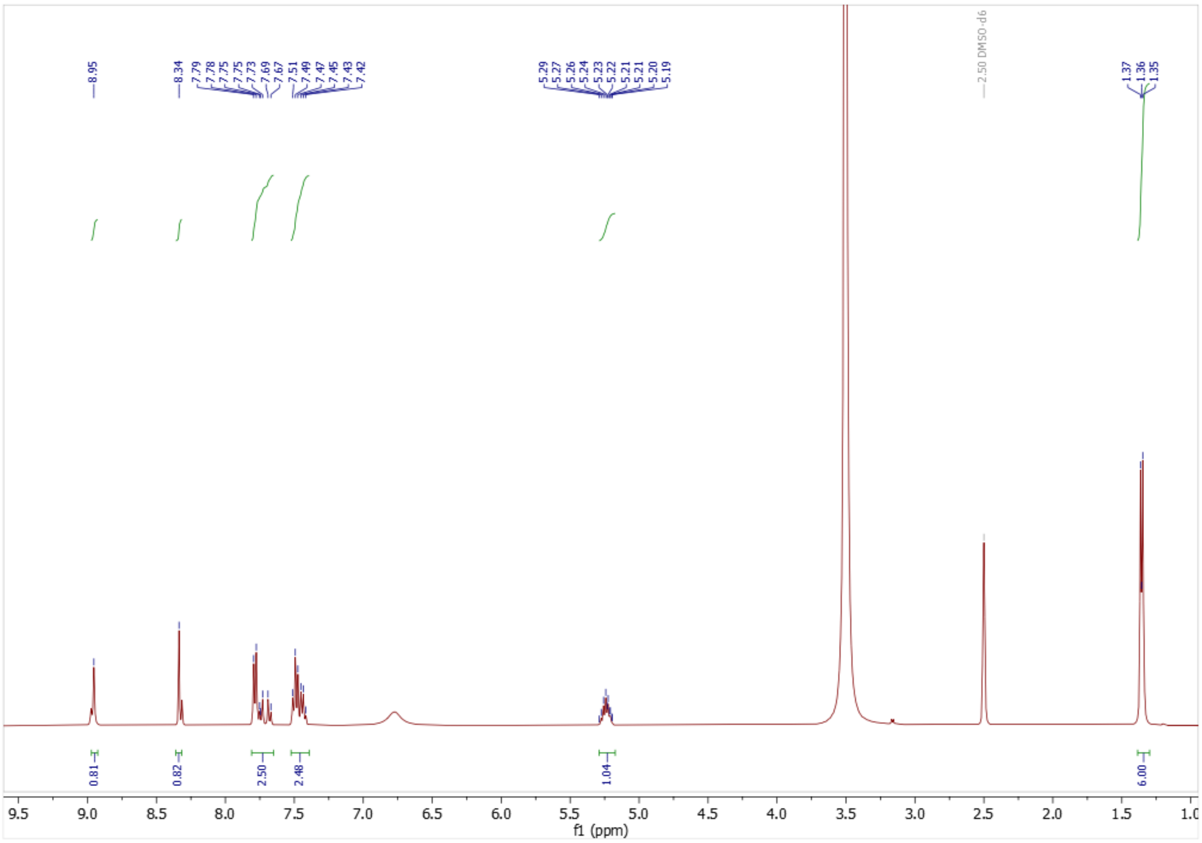

25e

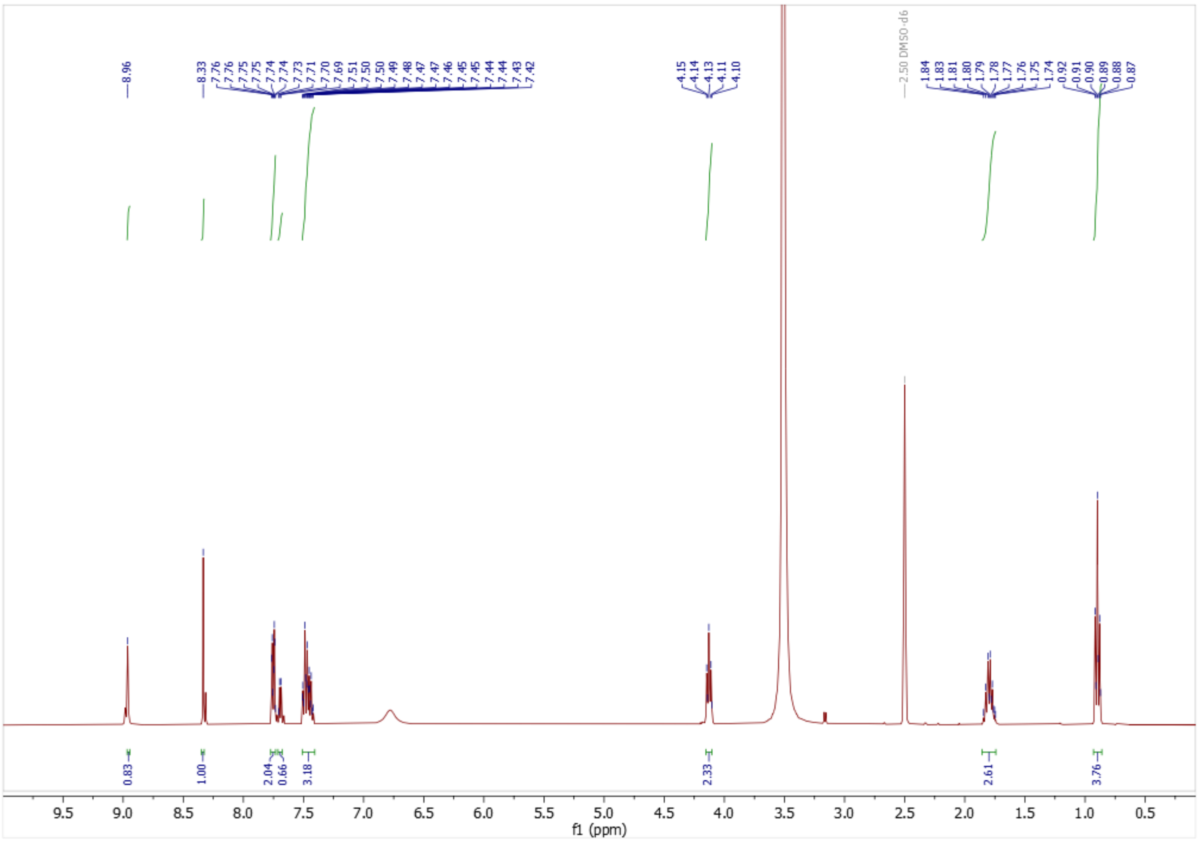

25f

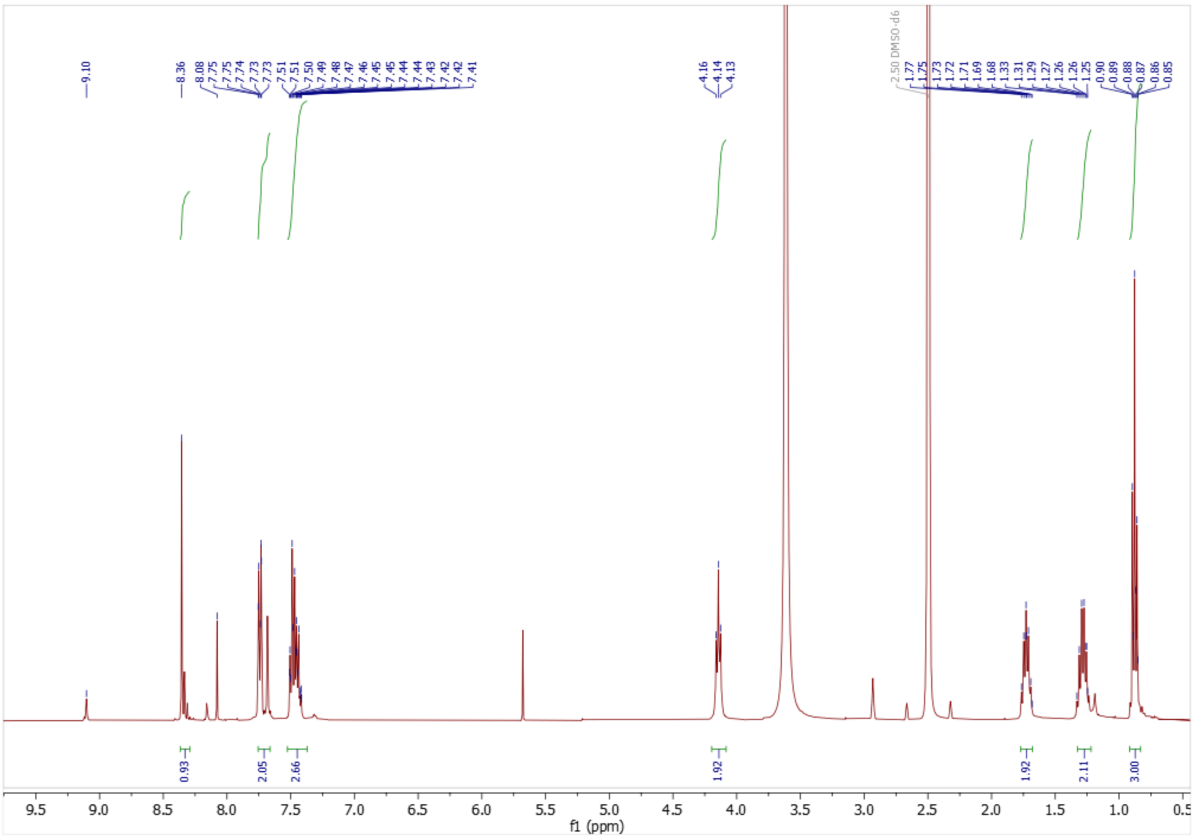

27

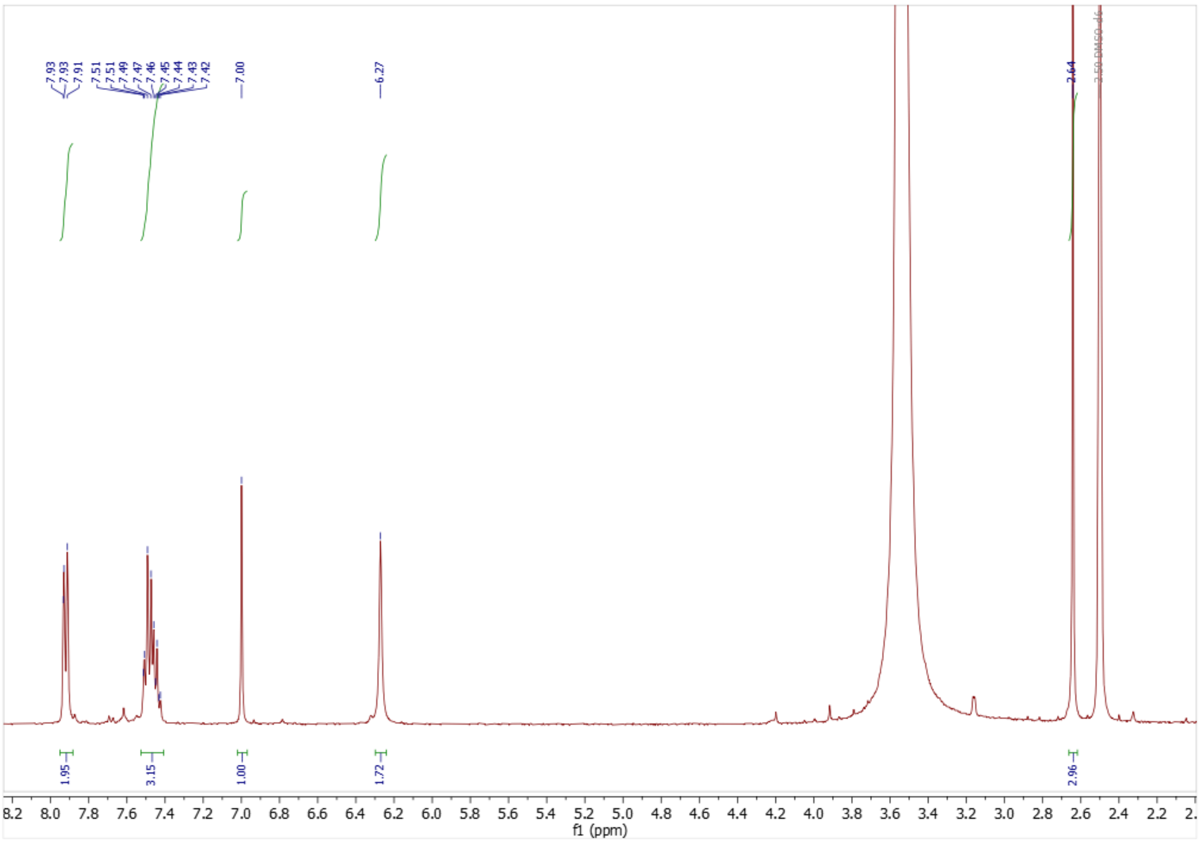
7.93
7.93
7.91
7.51
7.51
7.49
7.47
7.46
7.45
7.44
7.43
7.42
7.00
6.27
2.64
1.95
3.15
1.00
1.72
2.96
f1 (ppm)

28

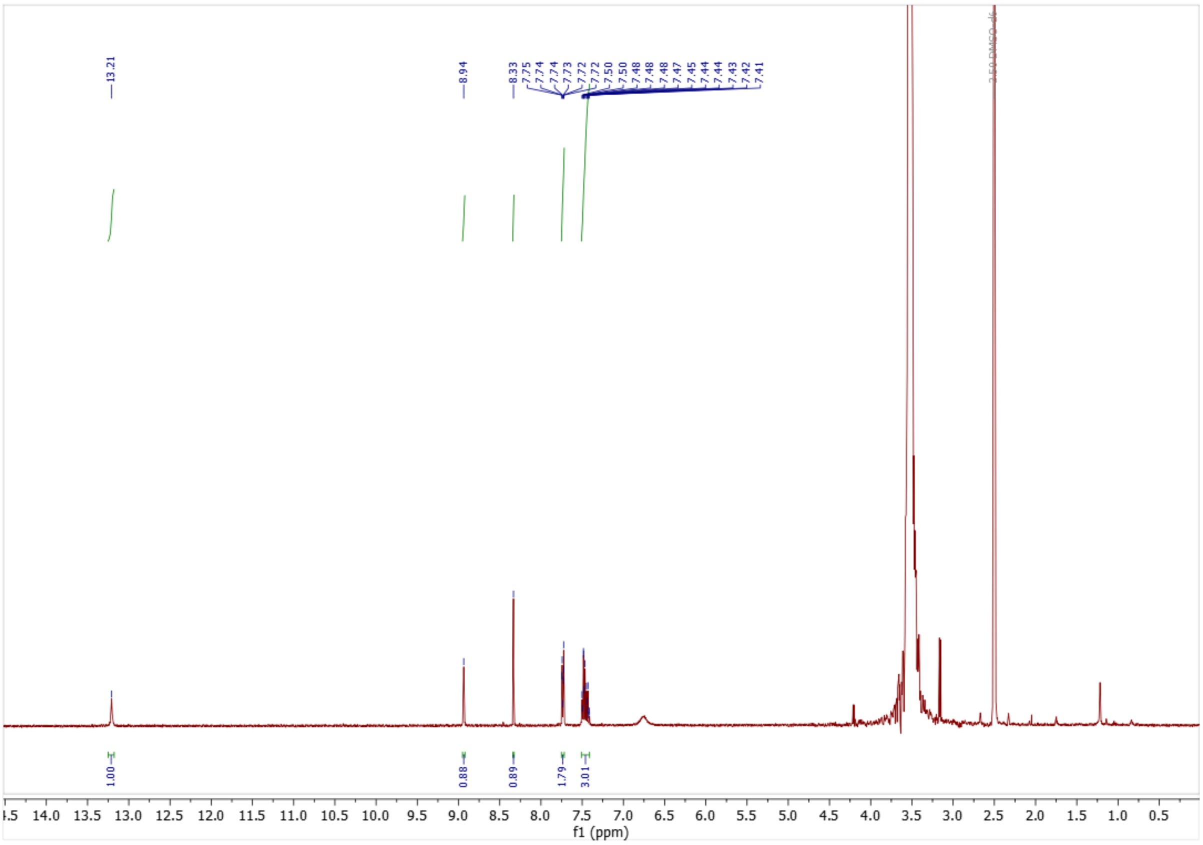
13.21
8.94
8.33
1.00
0.88
0.89
1.79
3.01
f1 (ppm)

29a

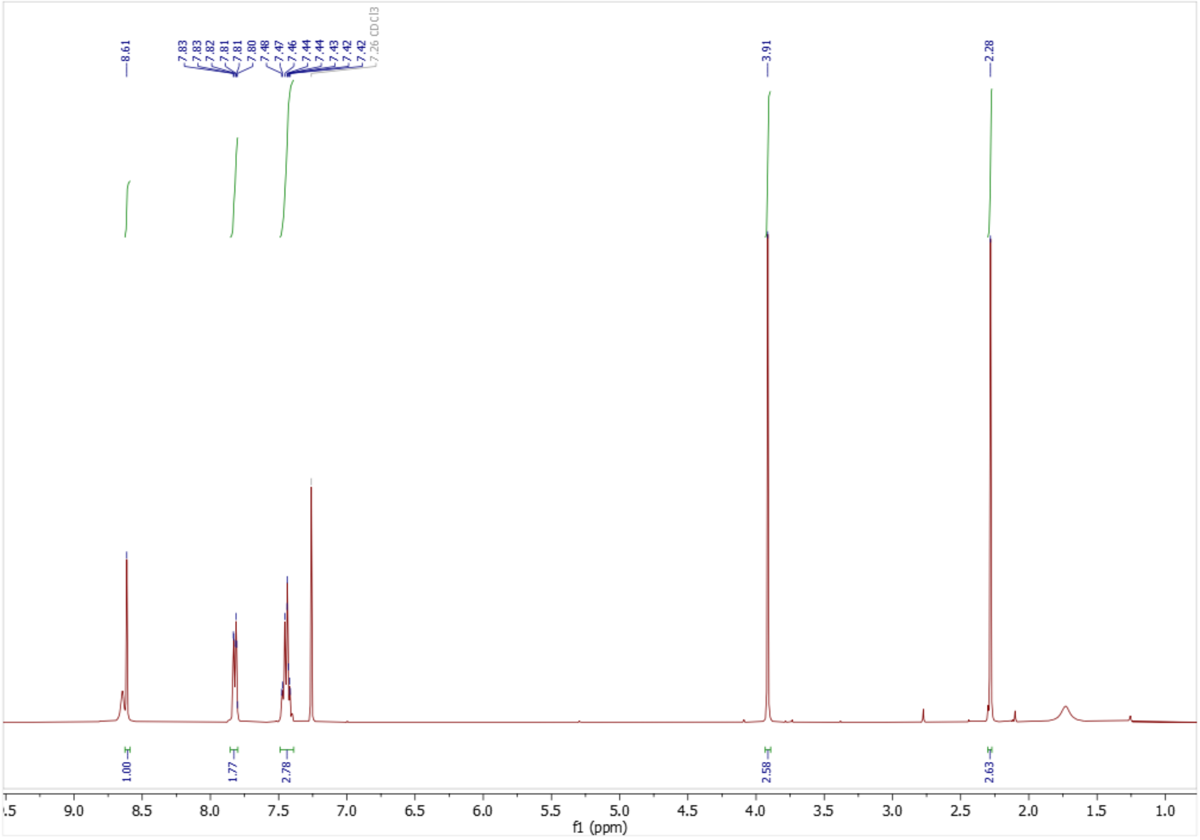
8.61
7.83
7.83
7.82
7.81
7.81
7.80
7.48
7.47
7.46
7.44
7.44
7.43
7.42
7.42
7.26 CDCl3
3.91
2.28
1.00
1.77
2.78
2.58
2.63
f1 (ppm)

29b

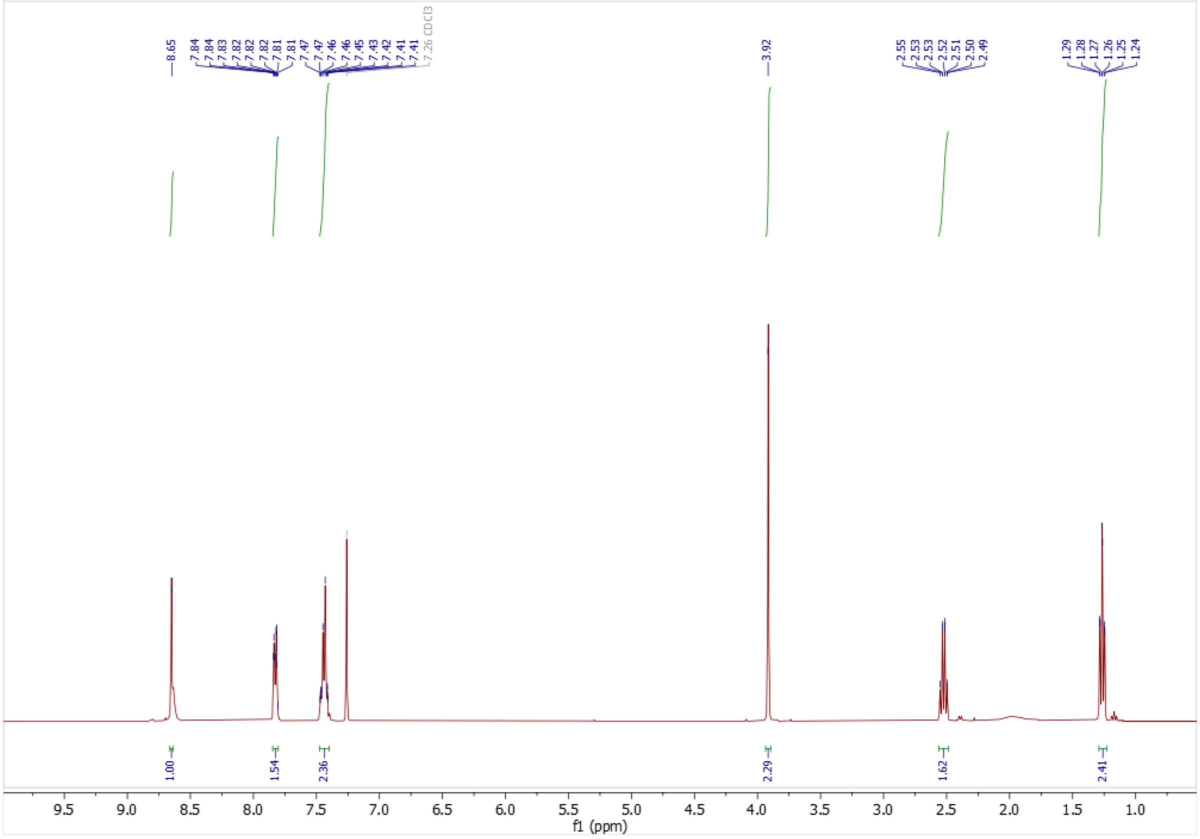
8.65
7.84
7.84
7.83
7.82
7.82
7.82
7.81
7.81
7.47
7.47
7.46
7.45
7.43
7.42
7.41
7.41
7.26 CDCl3
3.92
2.55
2.53
2.53
2.52
2.51
2.50
2.49
1.29
1.28
1.27
1.26
1.25
1.24
1.00
1.54
2.36
2.29
1.62
2.41
f1 (ppm)

29c

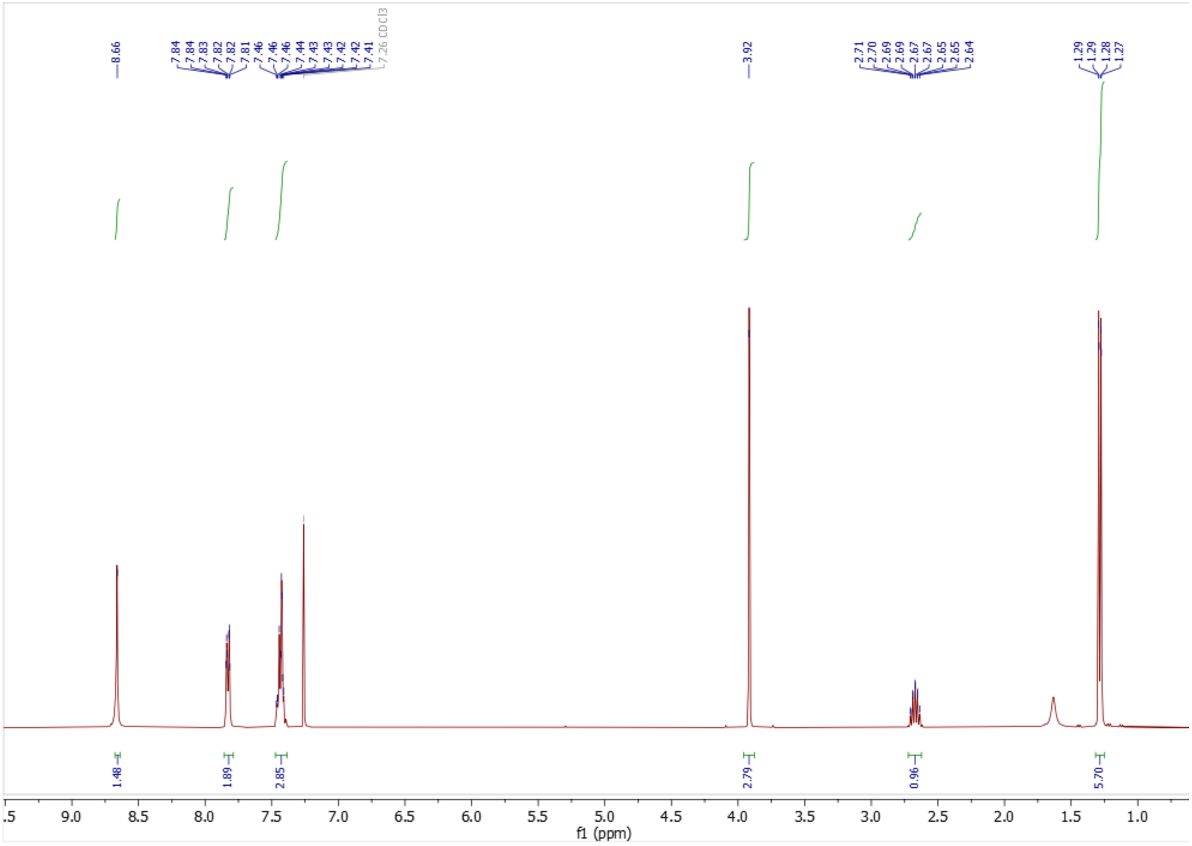

8.66
7.84
7.84
7.83
7.82
7.82
7.81
7.46
7.46
7.46
7.44
7.43
7.43
7.42
7.42
7.41
7.26 CDCl3
3.92
2.71
2.70
2.69
2.69
2.67
2.67
2.65
2.65
2.64
1.29
1.29
1.28
1.27
1.48
1.89
2.85
2.79
0.96
5.70
f1 (ppm)

30a

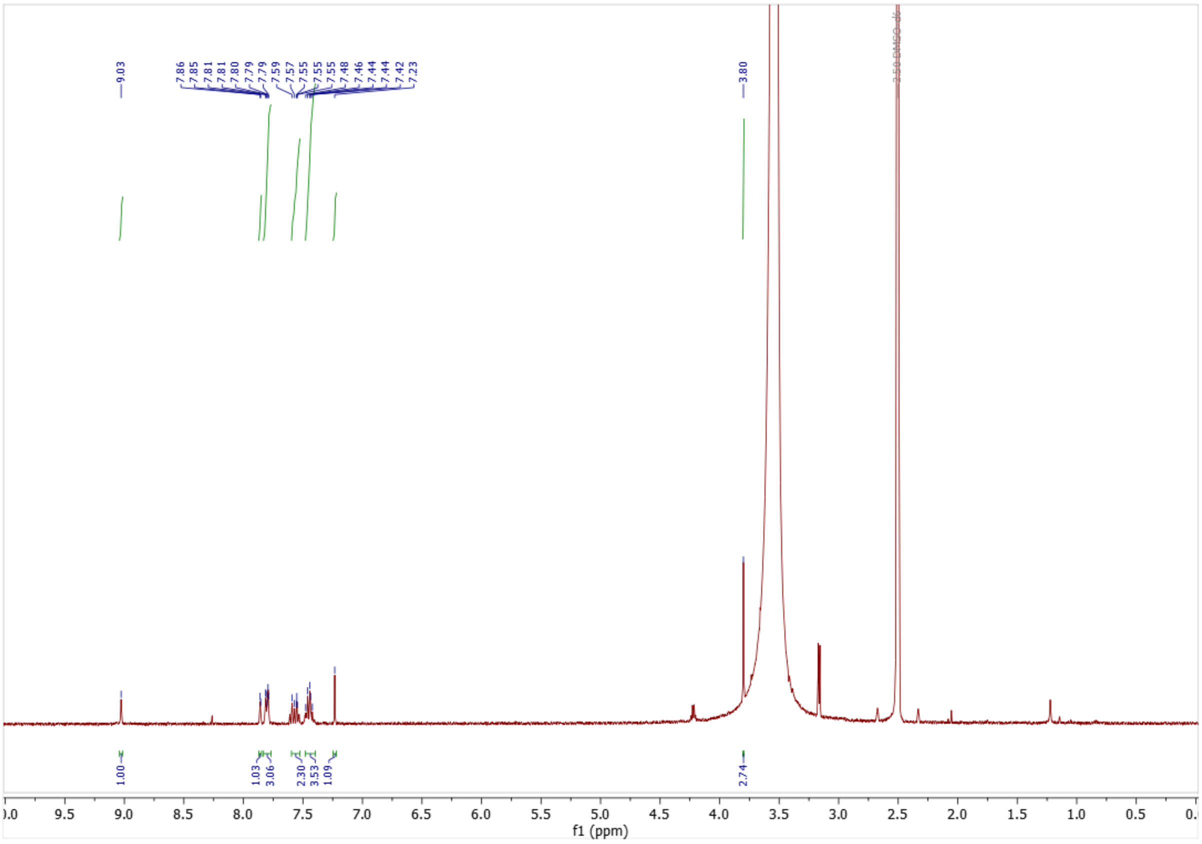

9.03
7.86
7.85
7.81
7.81
7.80
7.79
7.79
7.59
7.57
7.55
7.55
7.55
7.48
7.46
7.44
7.44
7.42
7.23
3.80
1.00
1.03
3.06
2.30
3.53
1.09
2.74
f1 (ppm)

30b

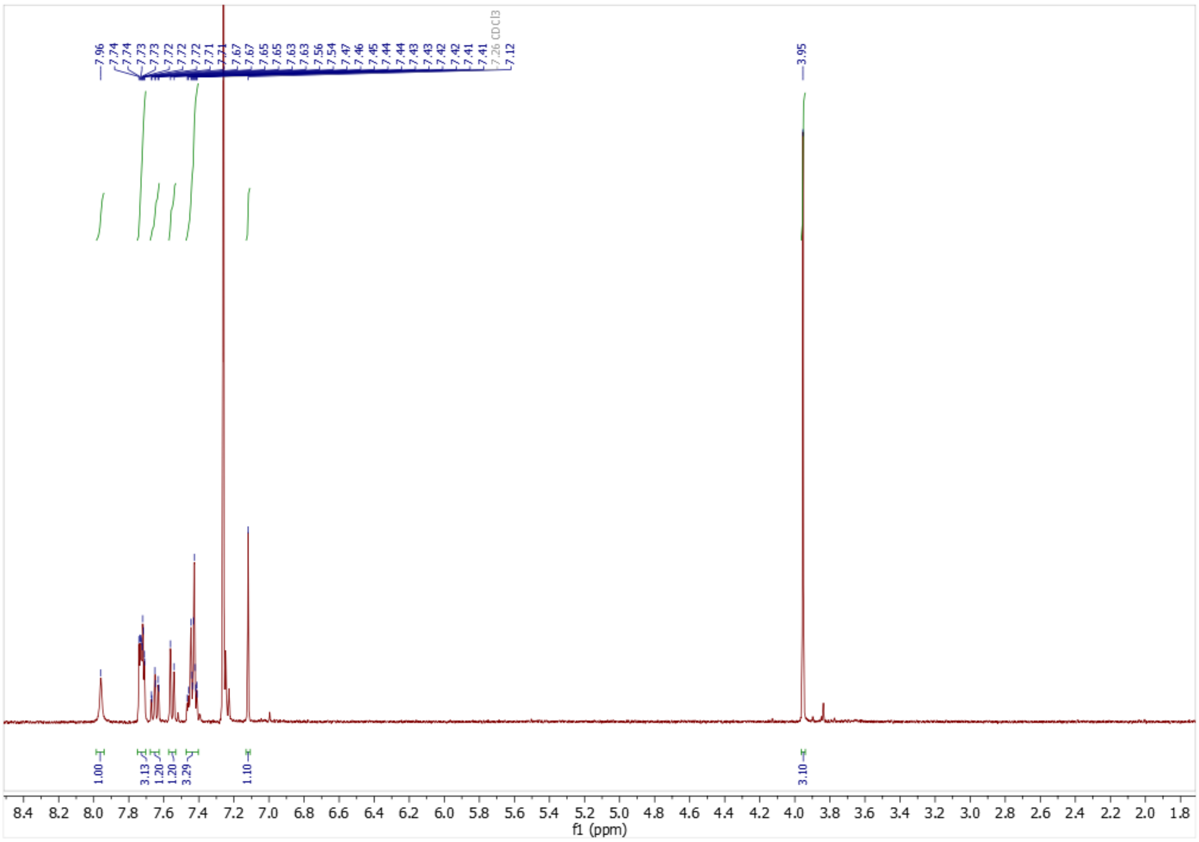

35

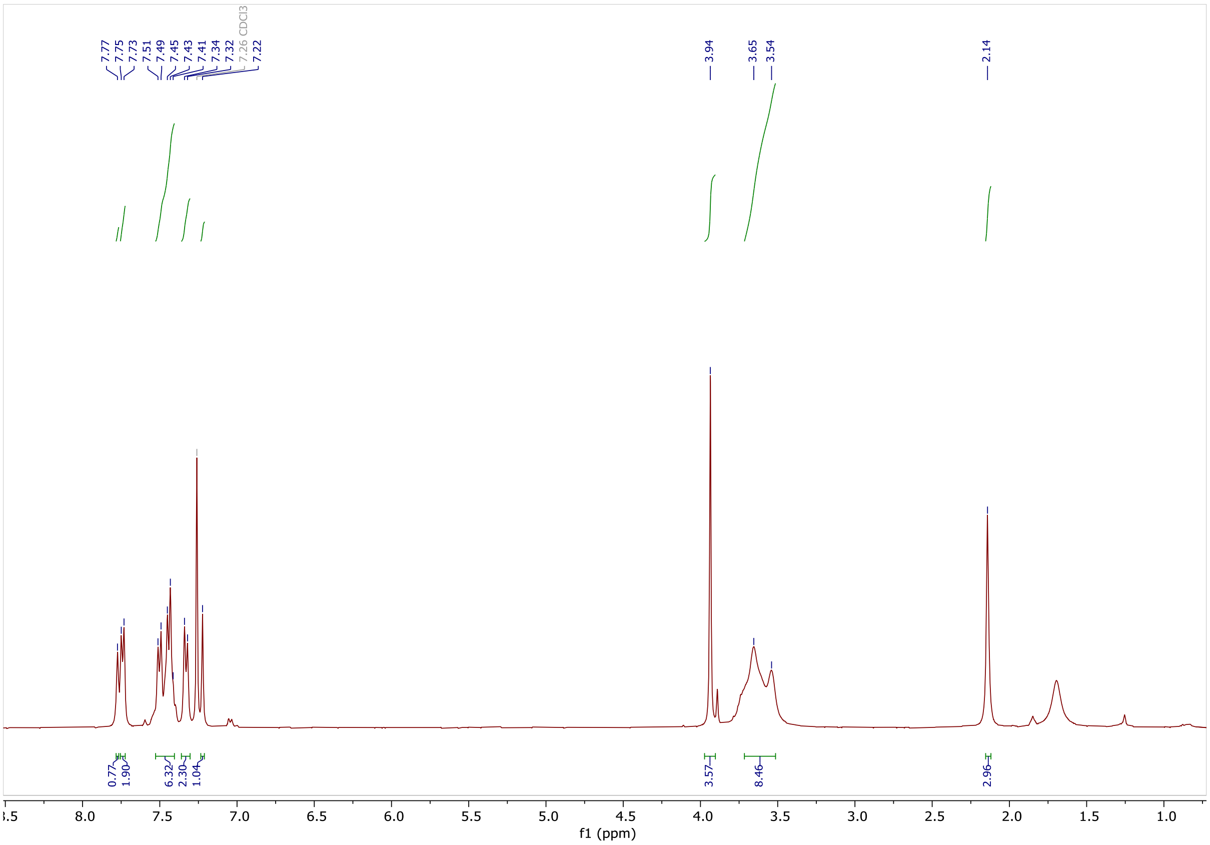

42a

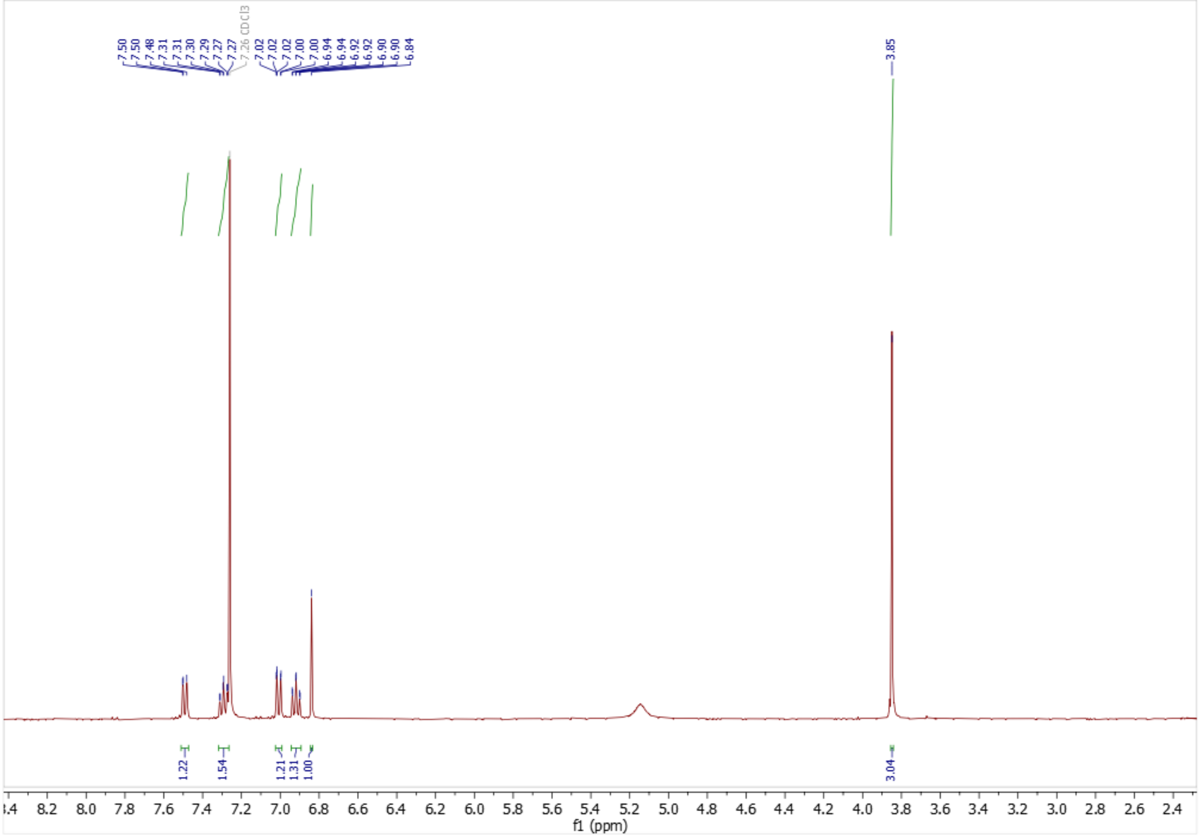
7.50
7.50
7.48
7.31
7.31
7.30
7.29
7.27
7.27
7.26 CDCl3
7.02
7.02
7.02
7.00
7.00
6.94
6.94
6.92
6.92
6.90
6.90
6.84
3.85
1.22
1.54
1.21
1.31
1.00
3.04
f1 (ppm)

51

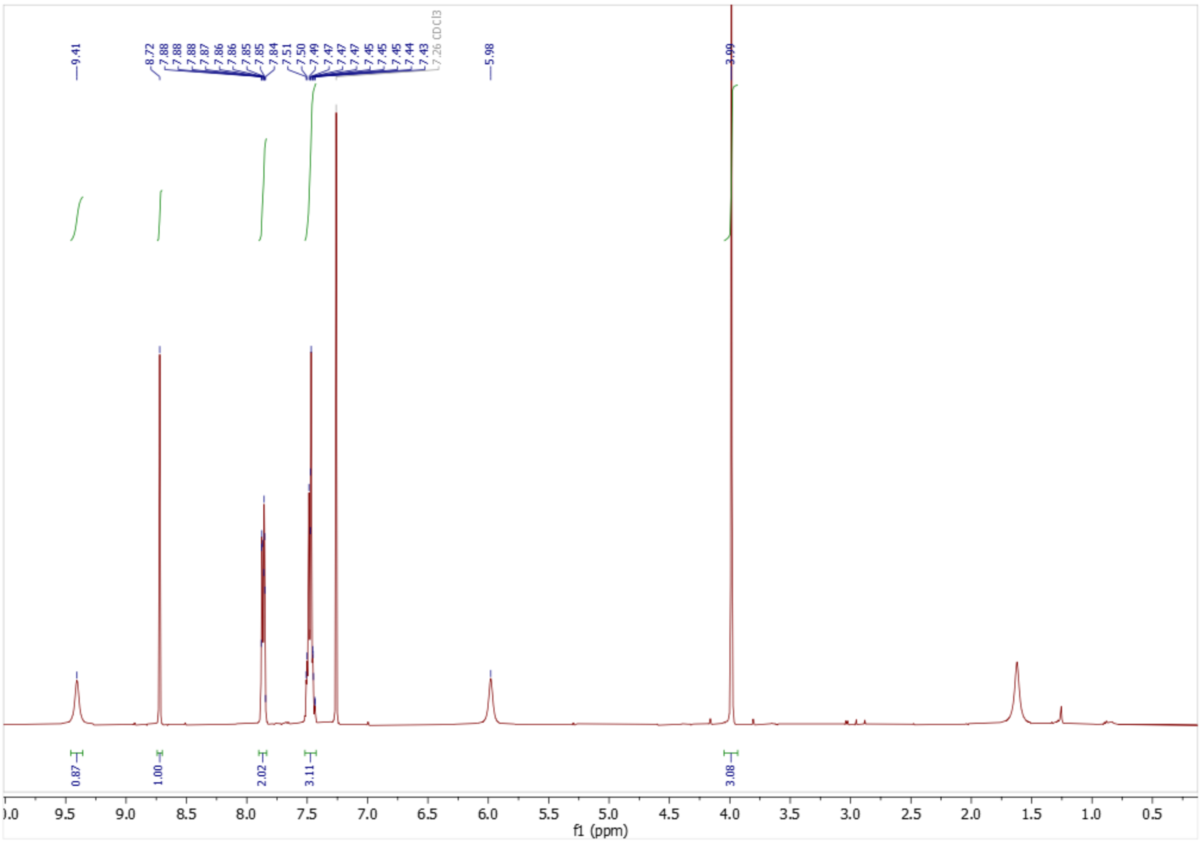
9.41
8.72
7.88
7.88
7.87
7.86
7.86
7.85
7.84
7.51
7.50
7.49
7.47
7.47
7.47
7.45
7.45
7.45
7.44
7.43
7.26 CDCl3
5.98
3.99
0.87
1.00
2.02
3.11
3.08
f1 (ppm)

54

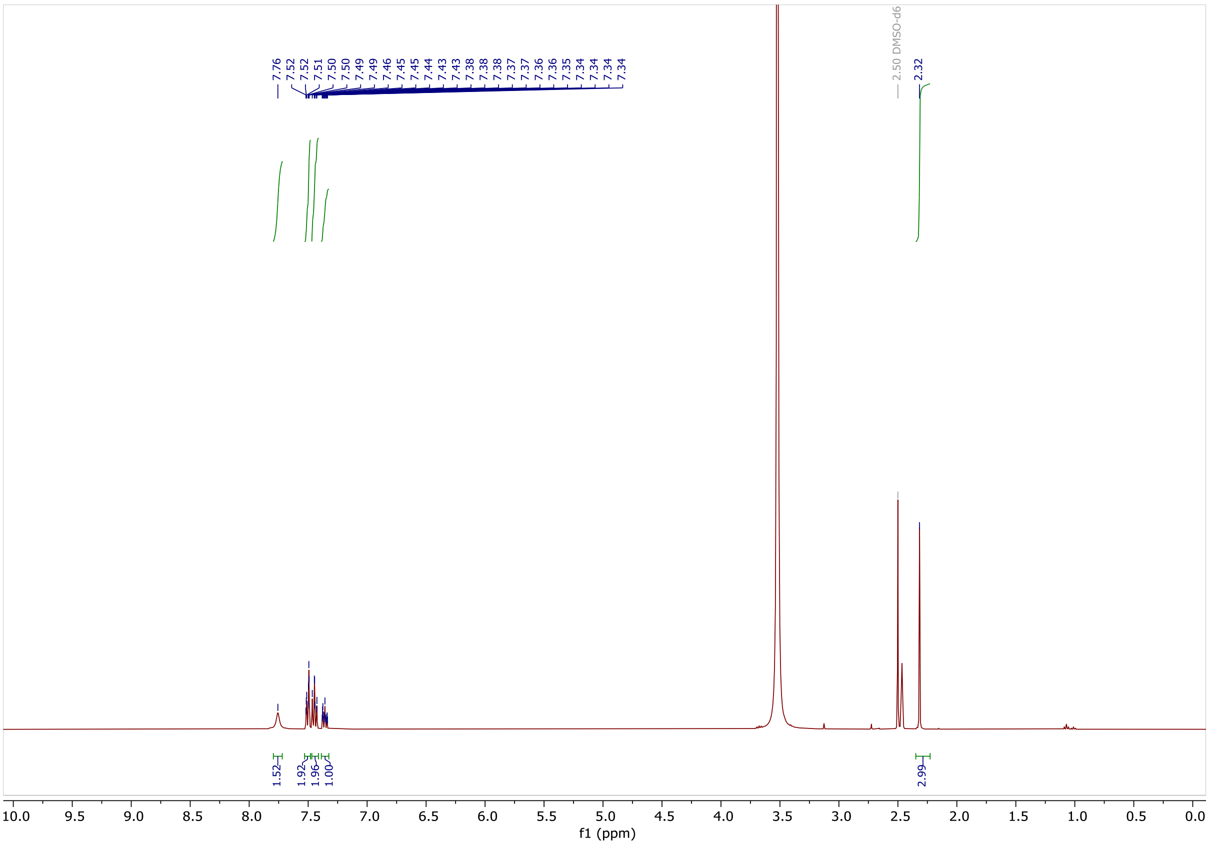

55

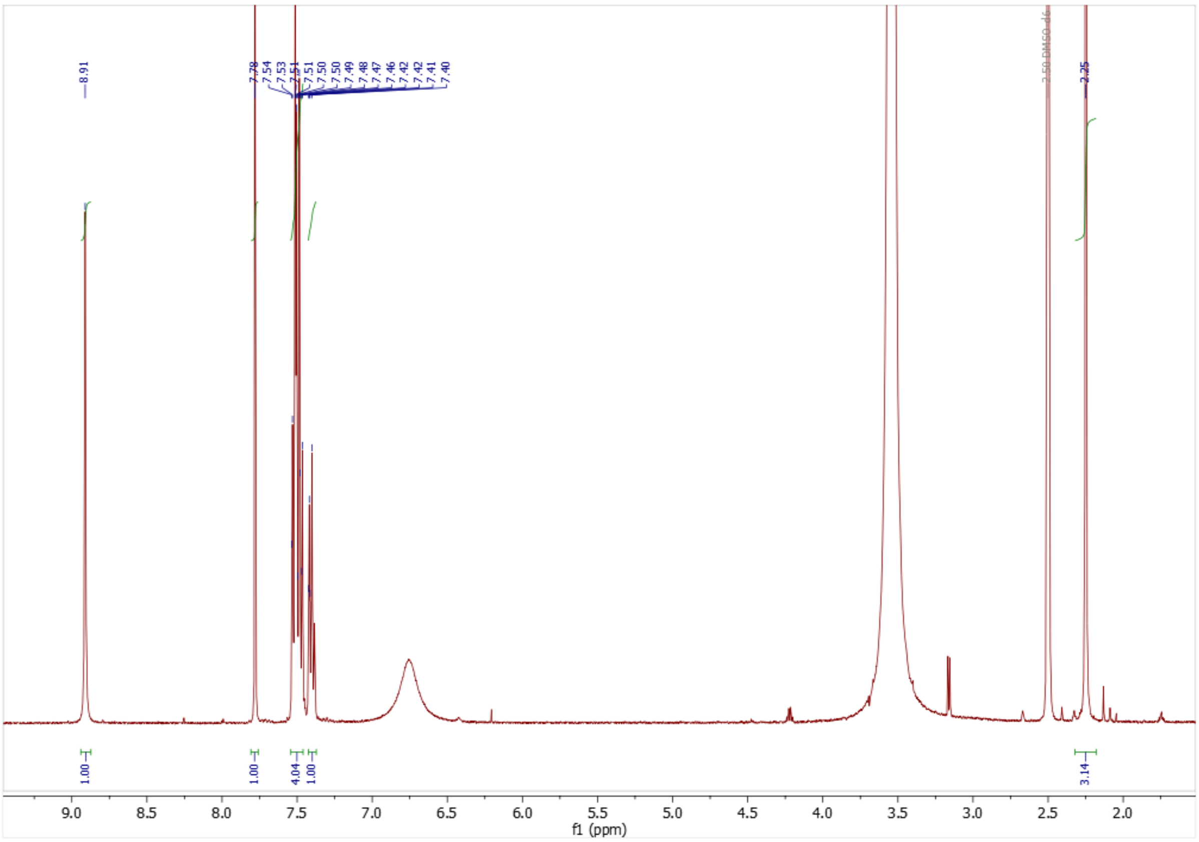

57

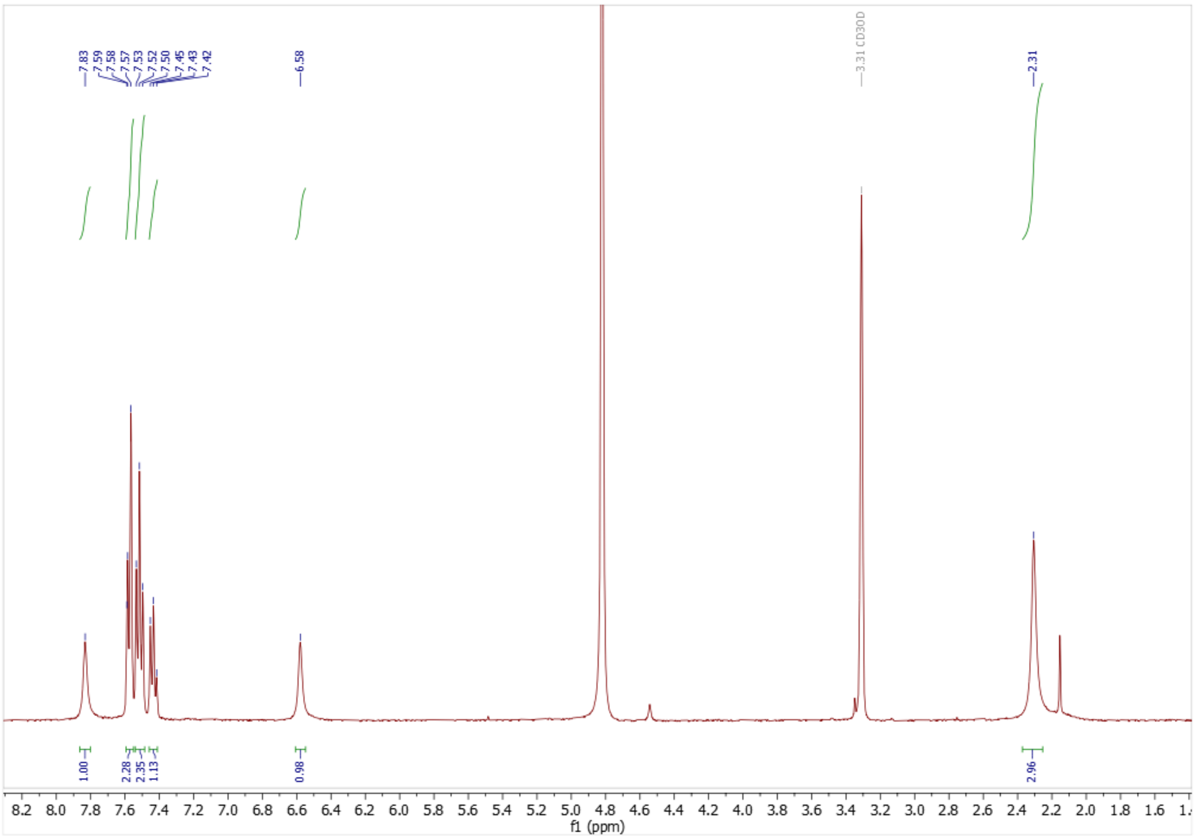
7.83
7.59
7.58
7.57
7.53
7.52
7.50
7.45
7.43
7.42
6.58
3.31 CD3OD
2.31
1.00
2.28
2.35
1.13
0.98
2.96
8.2 8.0 7.8 7.6 7.4 7.2 7.0 6.8 6.6 6.4 6.2 6.0 5.8 5.6 5.4 5.2 5.0 4.8 4.6 4.4 4.2 4.0 3.8 3.6 3.4 3.2 3.0 2.8 2.6 2.4 2.2 2.0 1.8 1.6
f1 (ppm)

^{13}C-NMRs

4a

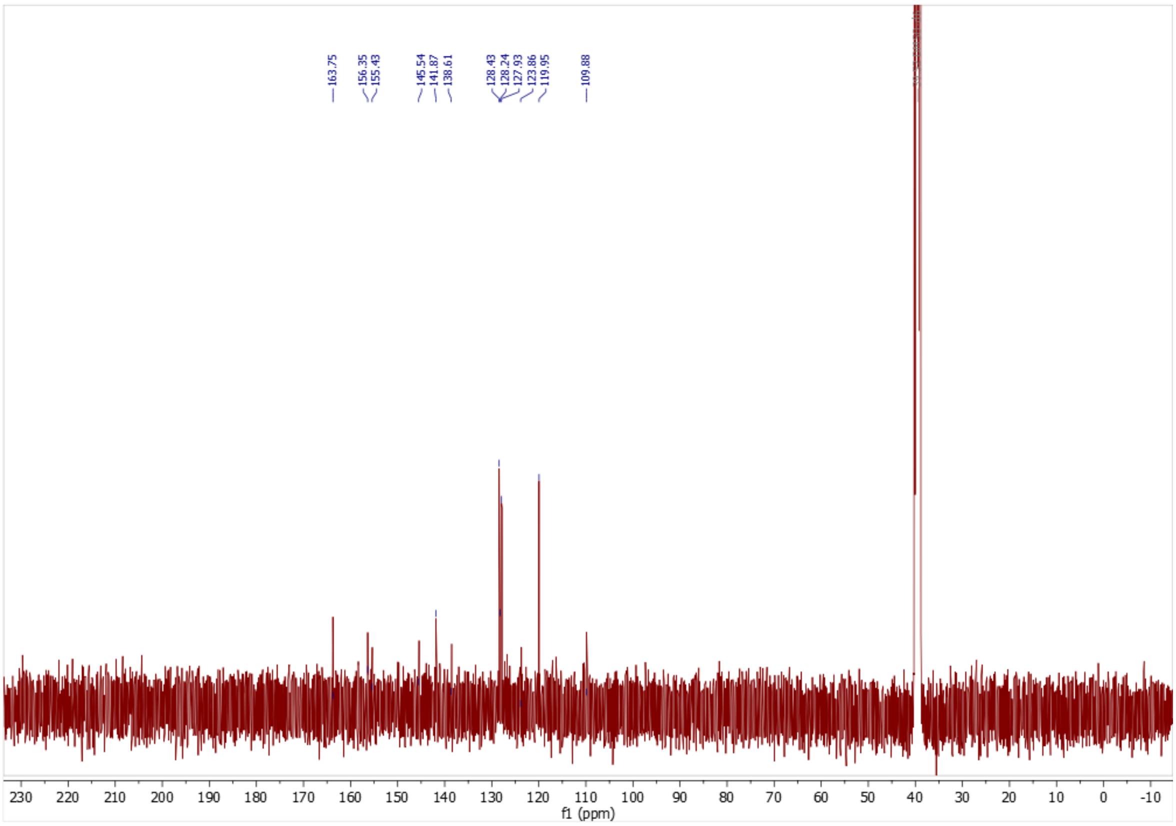

4b

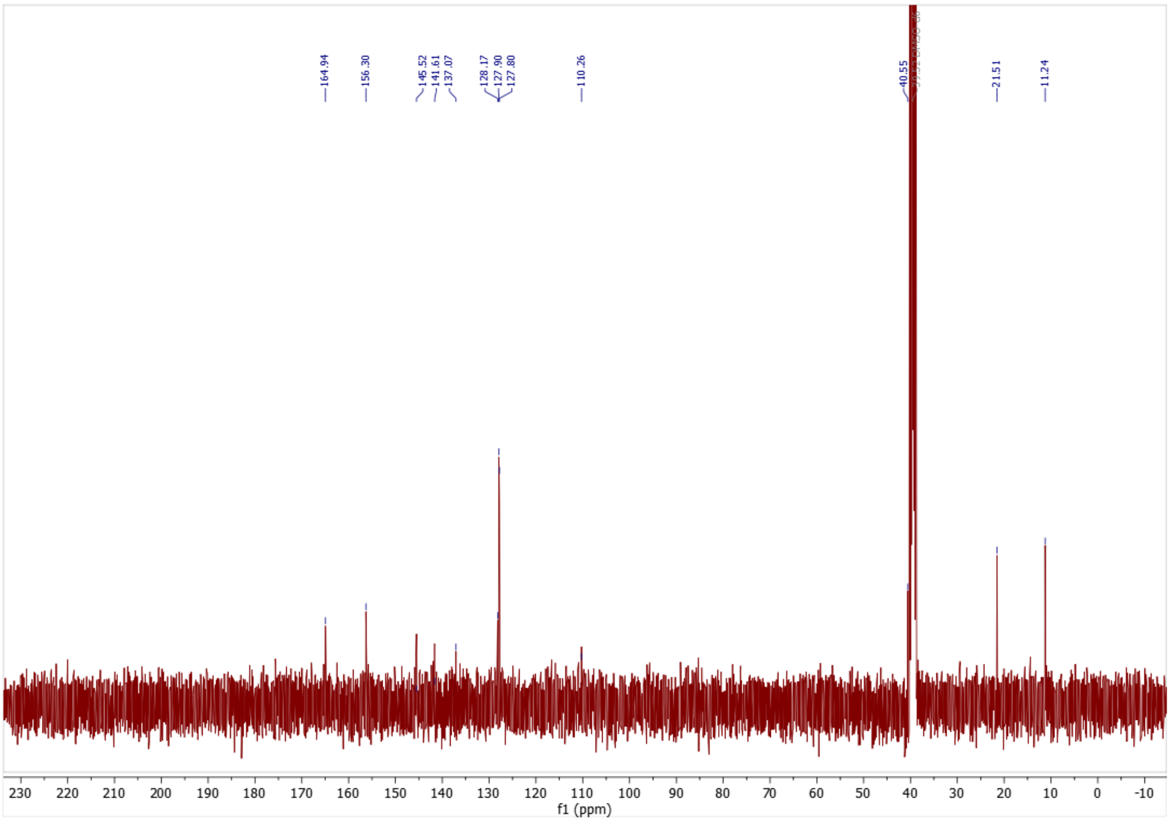

6

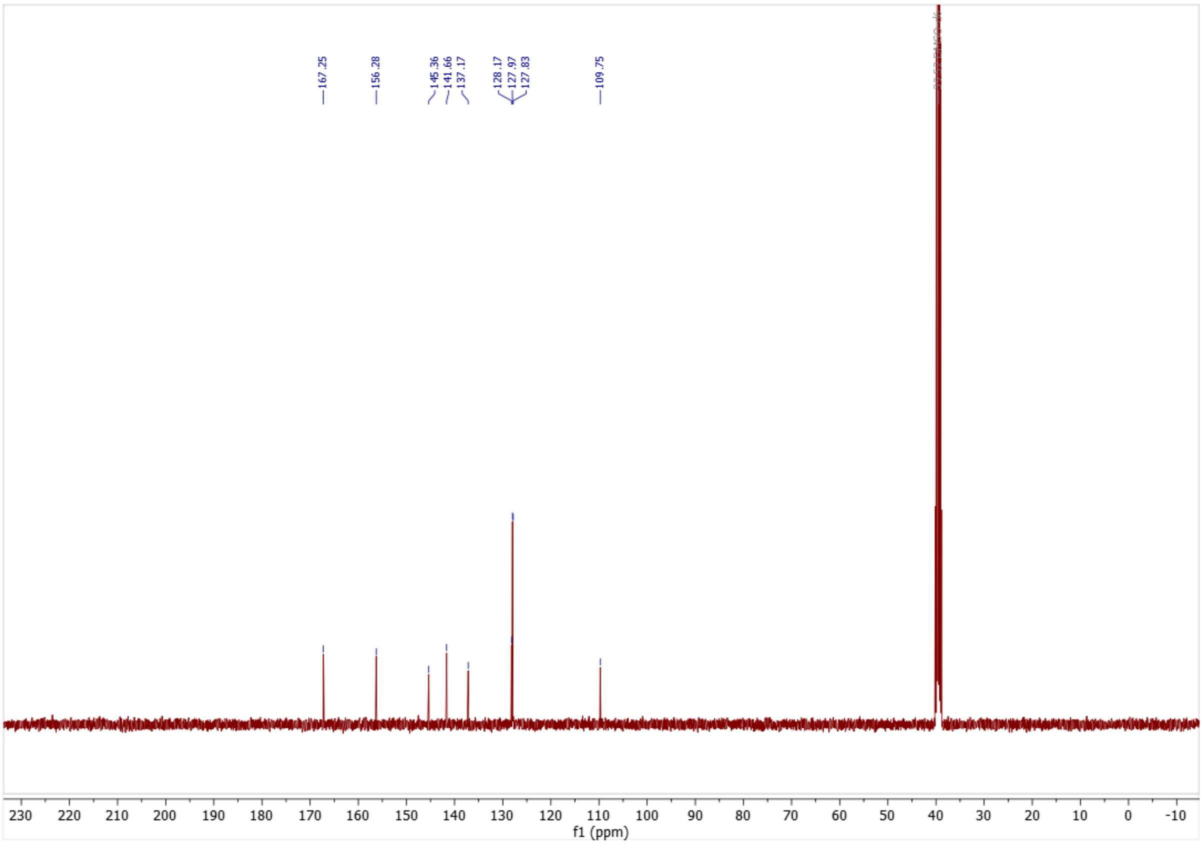
167.25
156.28
145.36
141.66
137.17
128.17
127.97
127.83
109.75
f1 (ppm)

7

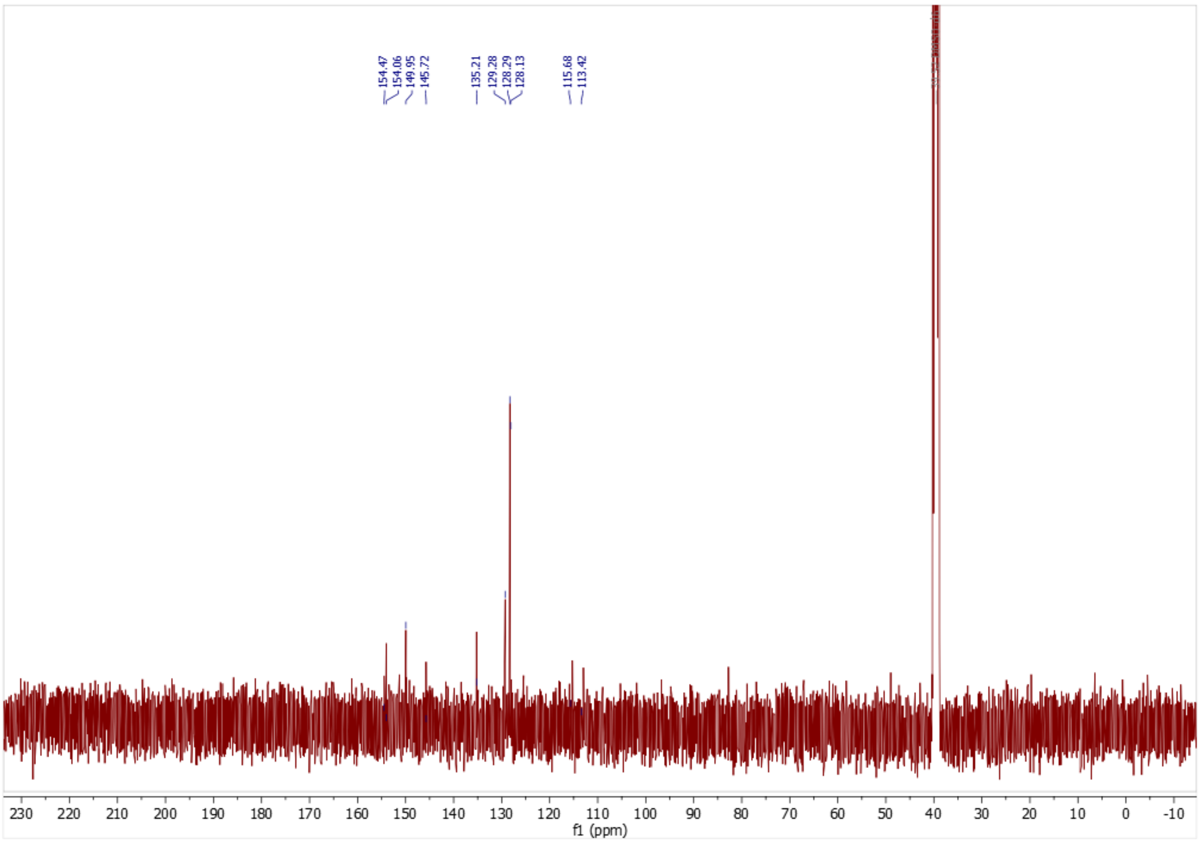
154.47
154.06
149.95
145.72
135.21
129.28
128.29
128.13
115.68
113.42
f1 (ppm)

16

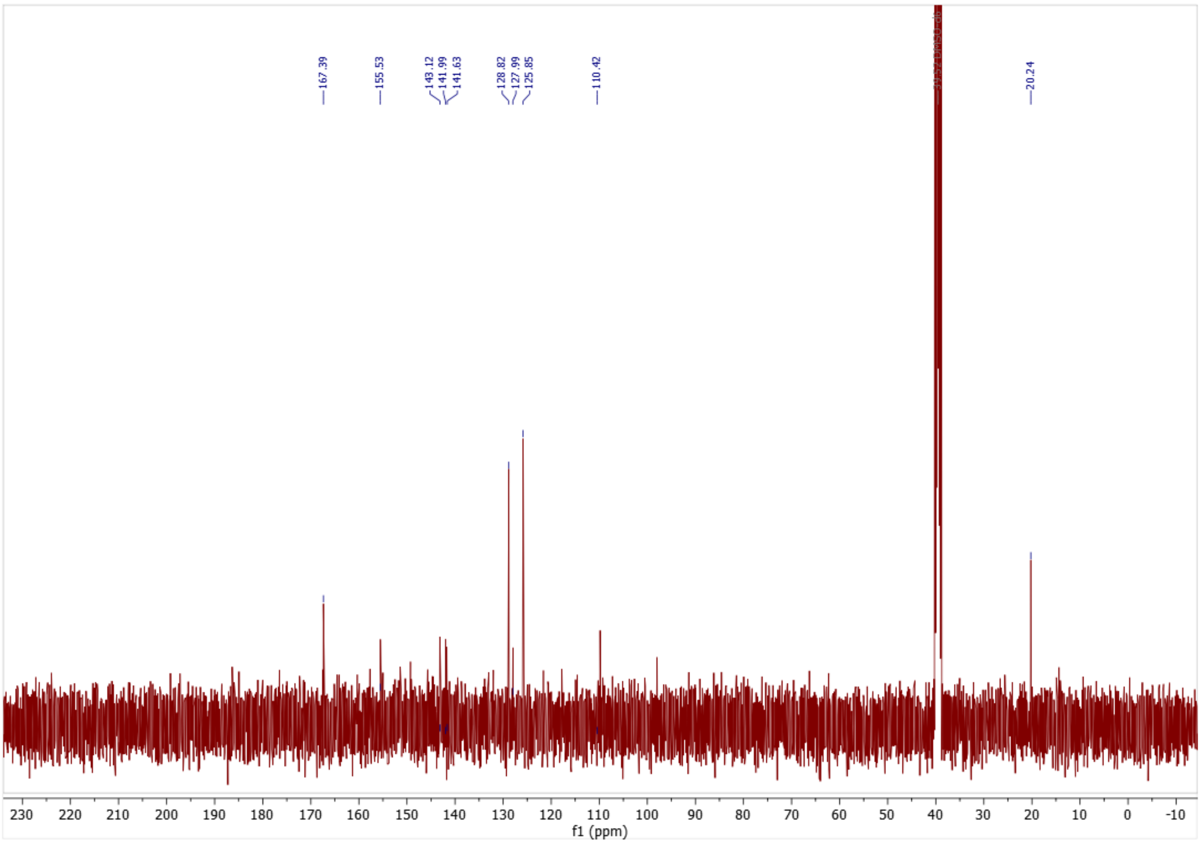
167.39
155.53
143.12
141.99
141.63
128.82
127.99
125.85
110.42
20.24
230 220 210 200 190 180 170 160 150 140 130 120 110 100 90 80 70 60 50 40 30 20 10 0 -10
f1 (ppm)

17

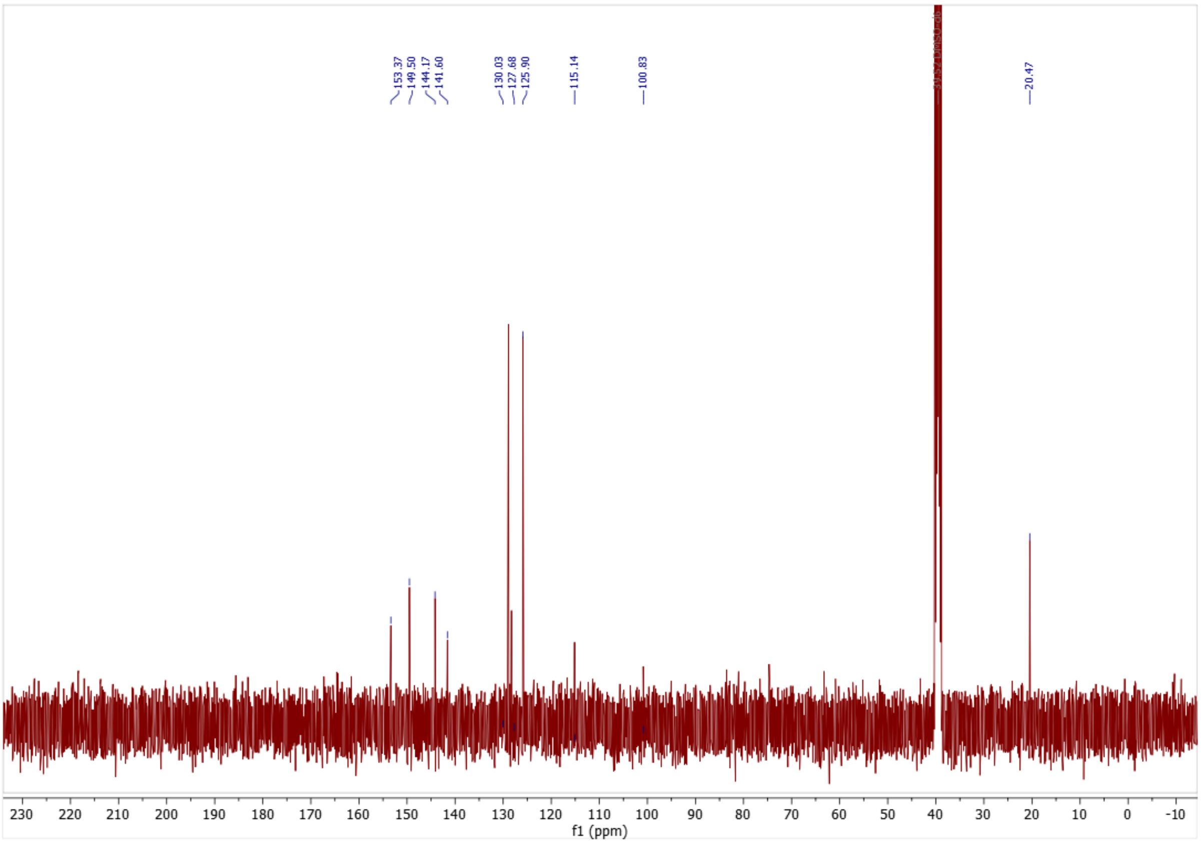
153.37
149.50
144.17
141.60
130.03
127.68
125.90
115.14
100.83
20.47
230 220 210 200 190 180 170 160 150 140 130 120 110 100 90 80 70 60 50 40 30 20 10 0 -10
f1 (ppm)

22

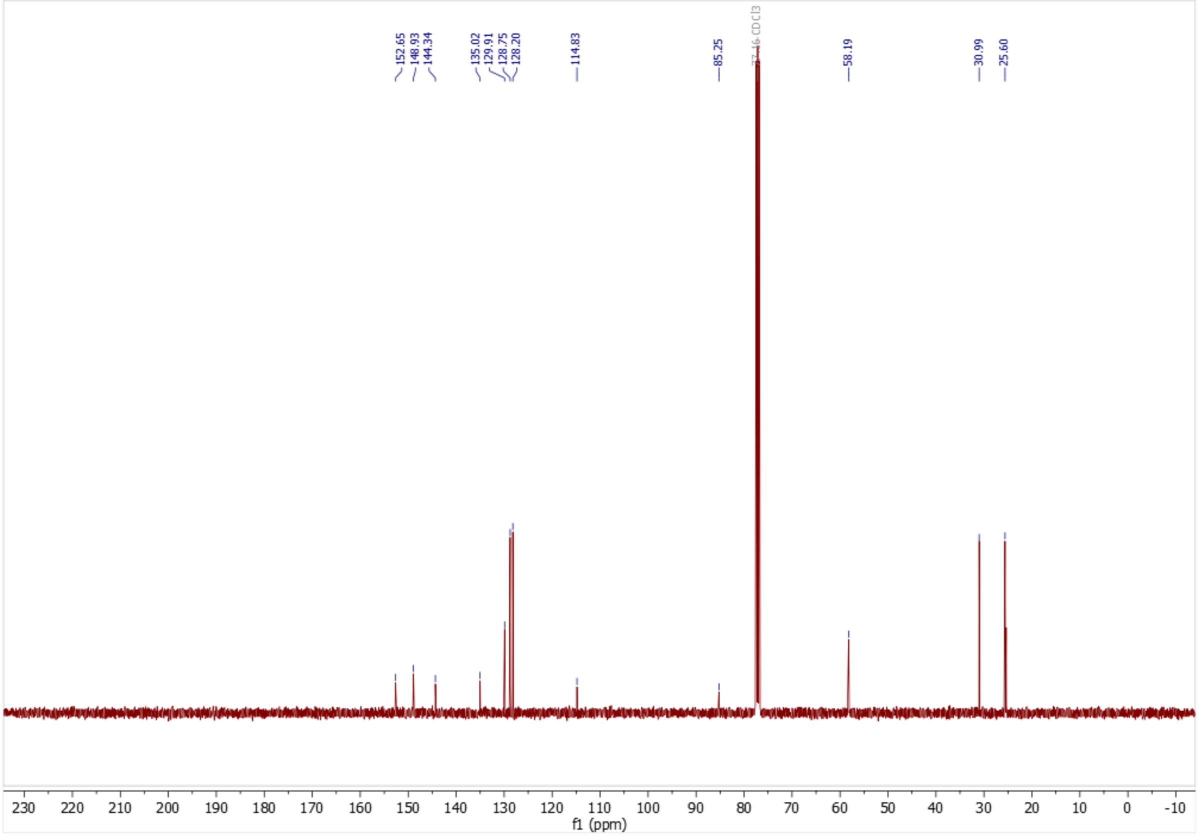

25a (EtOAc crossed)

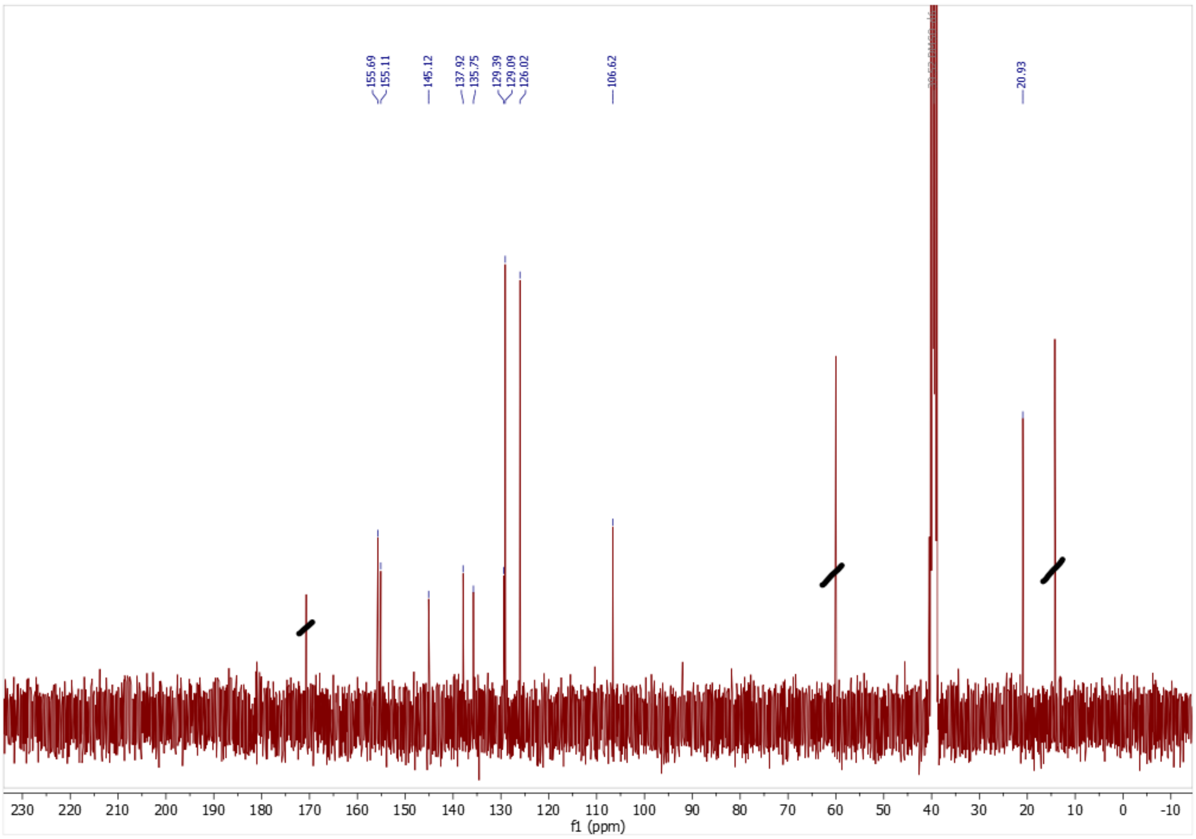

25c

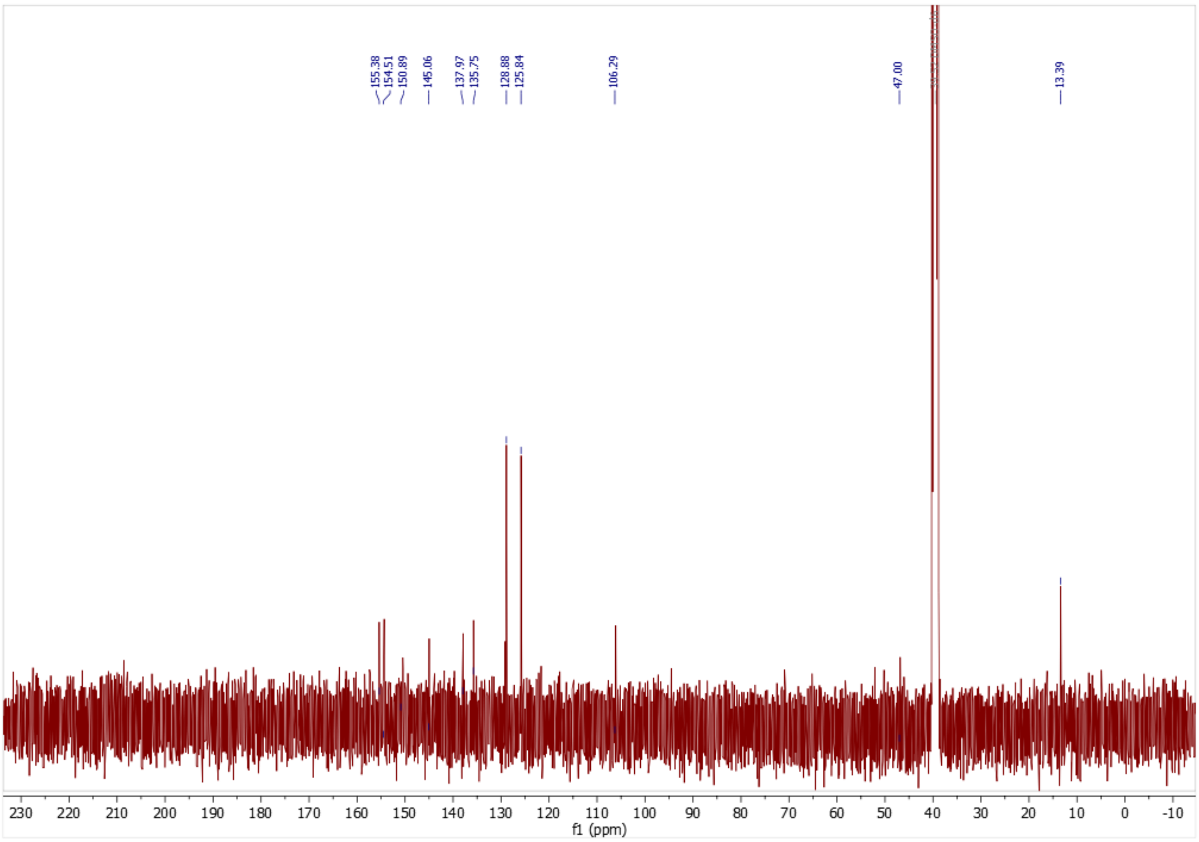

155.38
154.51
150.89
145.06
137.97
135.75
128.88
125.84
106.29
47.00
13.39
230 220 210 200 190 180 170 160 150 140 130 120 110 100 90 80 70 60 50 40 30 20 10 0 -10
f1 (ppm)

25d

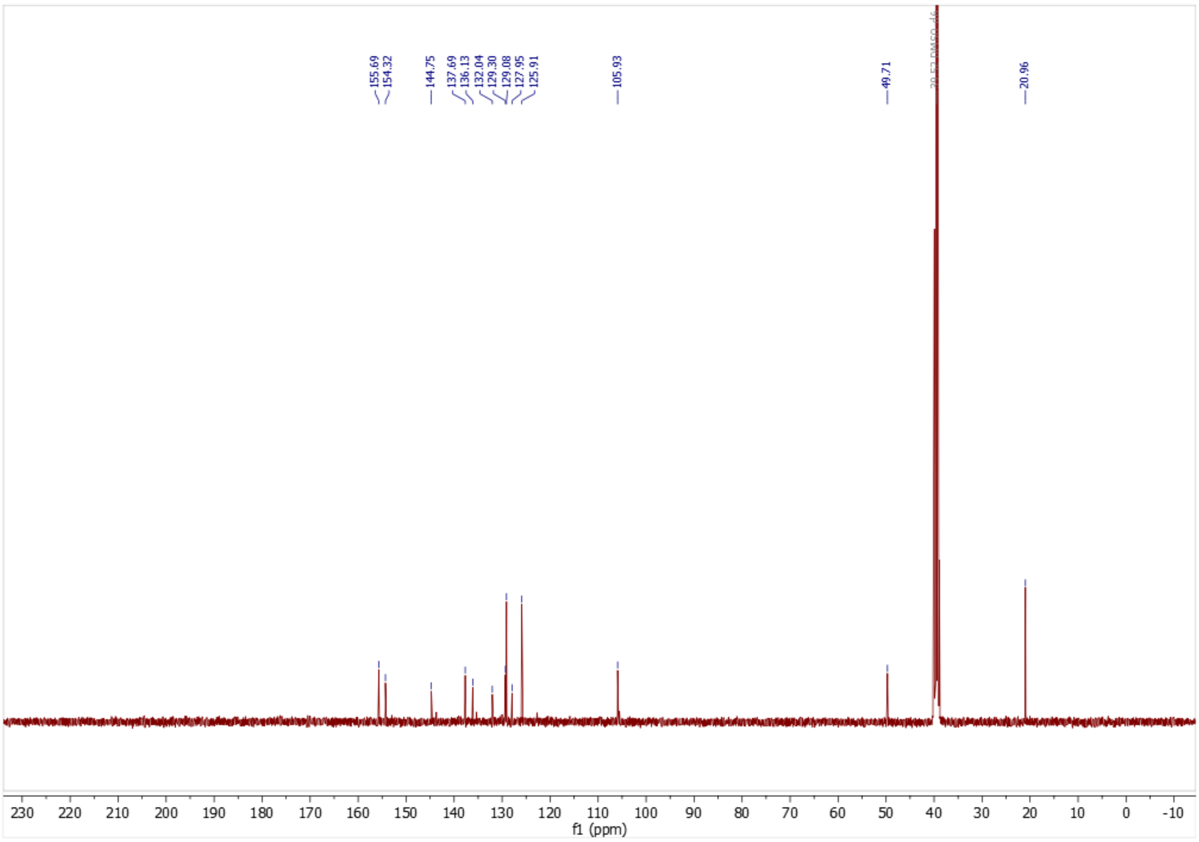

155.69
154.32
144.75
137.69
136.13
132.04
129.30
129.08
127.95
125.91
105.93
49.71
20.96
230 220 210 200 190 180 170 160 150 140 130 120 110 100 90 80 70 60 50 40 30 20 10 0 -10
f1 (ppm)

25e

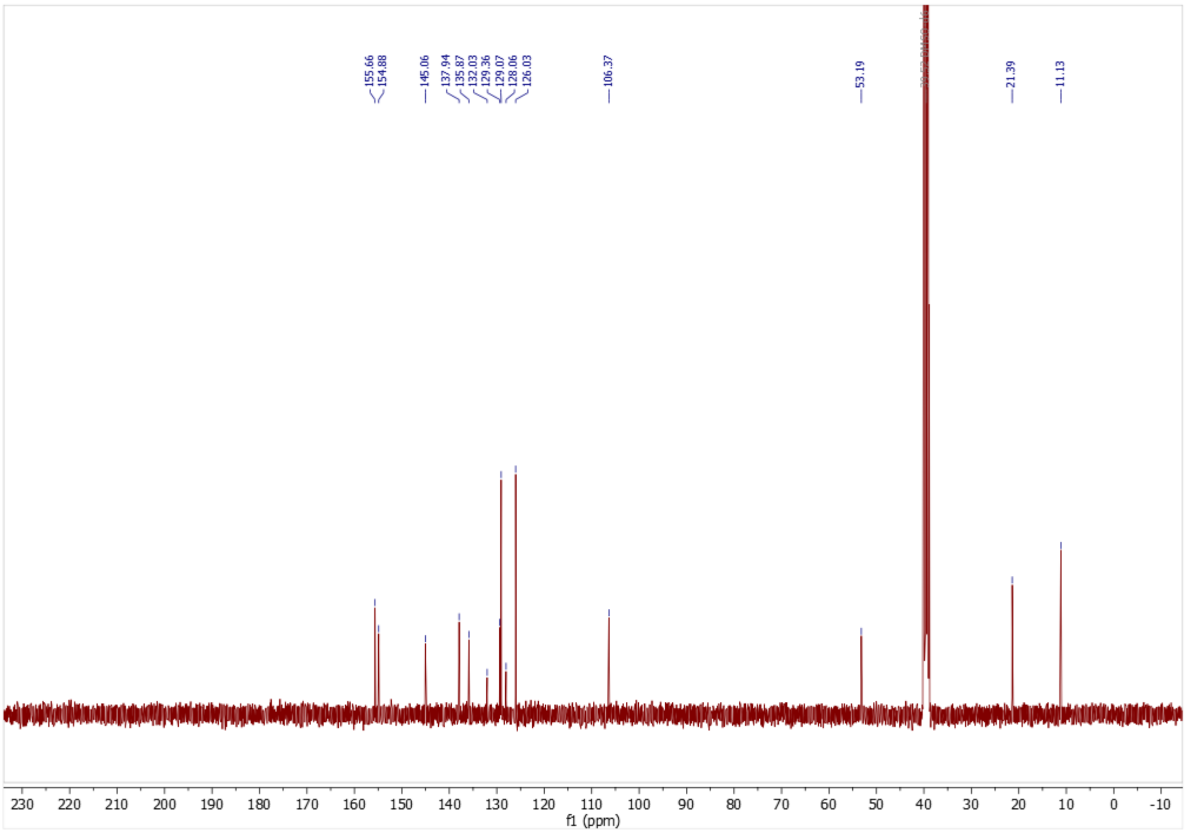
155.66
154.88
145.06
137.94
135.87
132.03
129.36
129.07
128.06
126.03
106.37
53.19
21.39
11.13
f1 (ppm)

25f

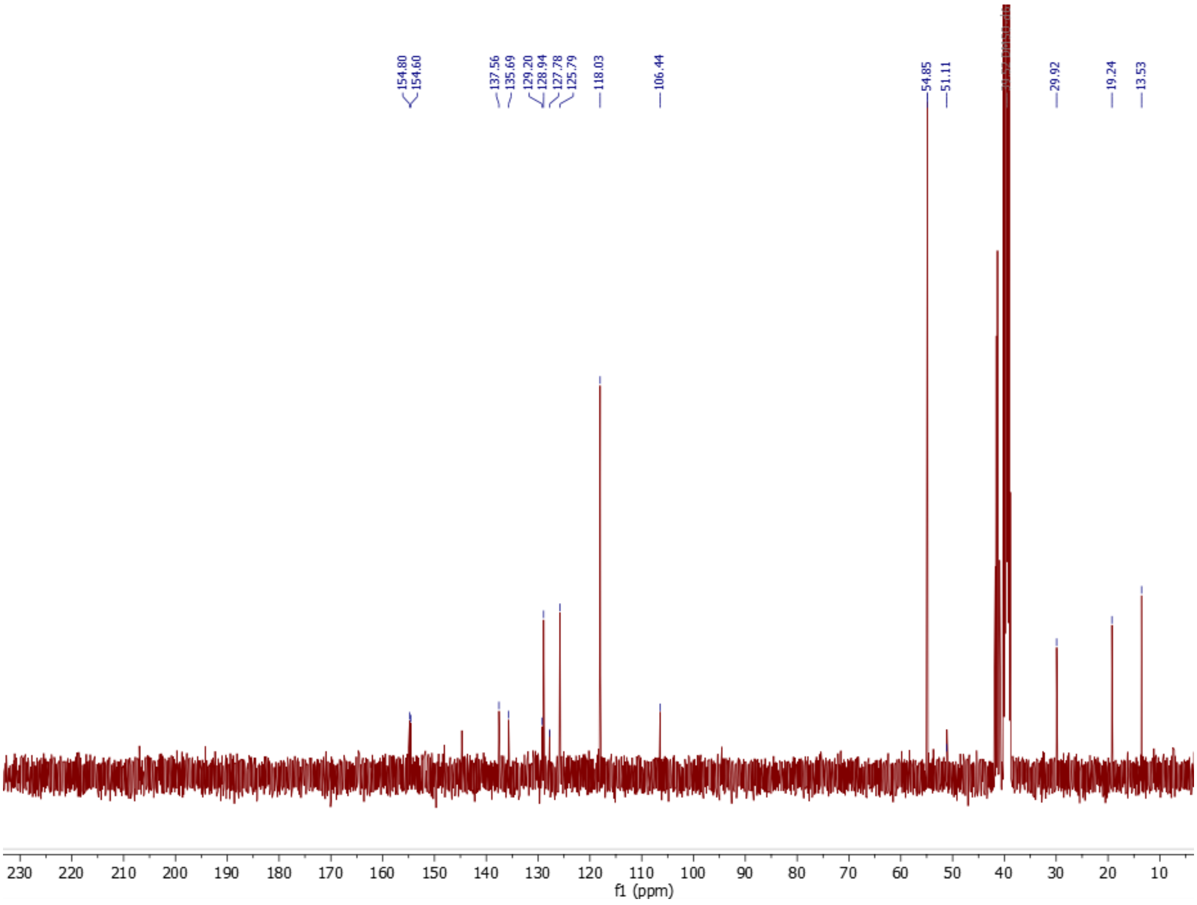
154.80
154.60
137.56
135.69
129.20
128.94
127.78
125.79
118.03
106.44
54.85
51.11
29.92
19.24
13.53
f1 (ppm)

27

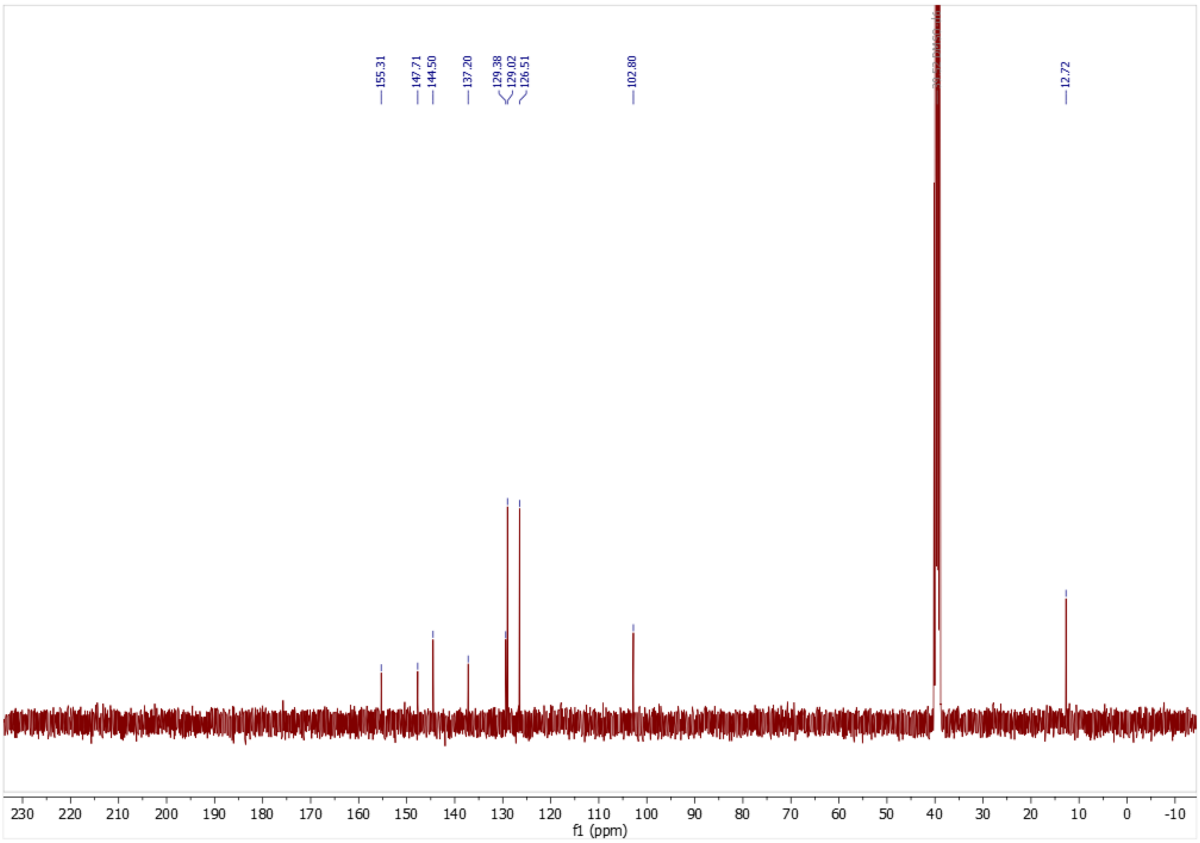
155.31
147.71
144.50
137.20
129.38
129.02
126.51
102.80
12.72
230 220 210 200 190 180 170 160 150 140 130 120 110 100 90 80 70 60 50 40 30 20 10 0 -10
f1 (ppm)

28

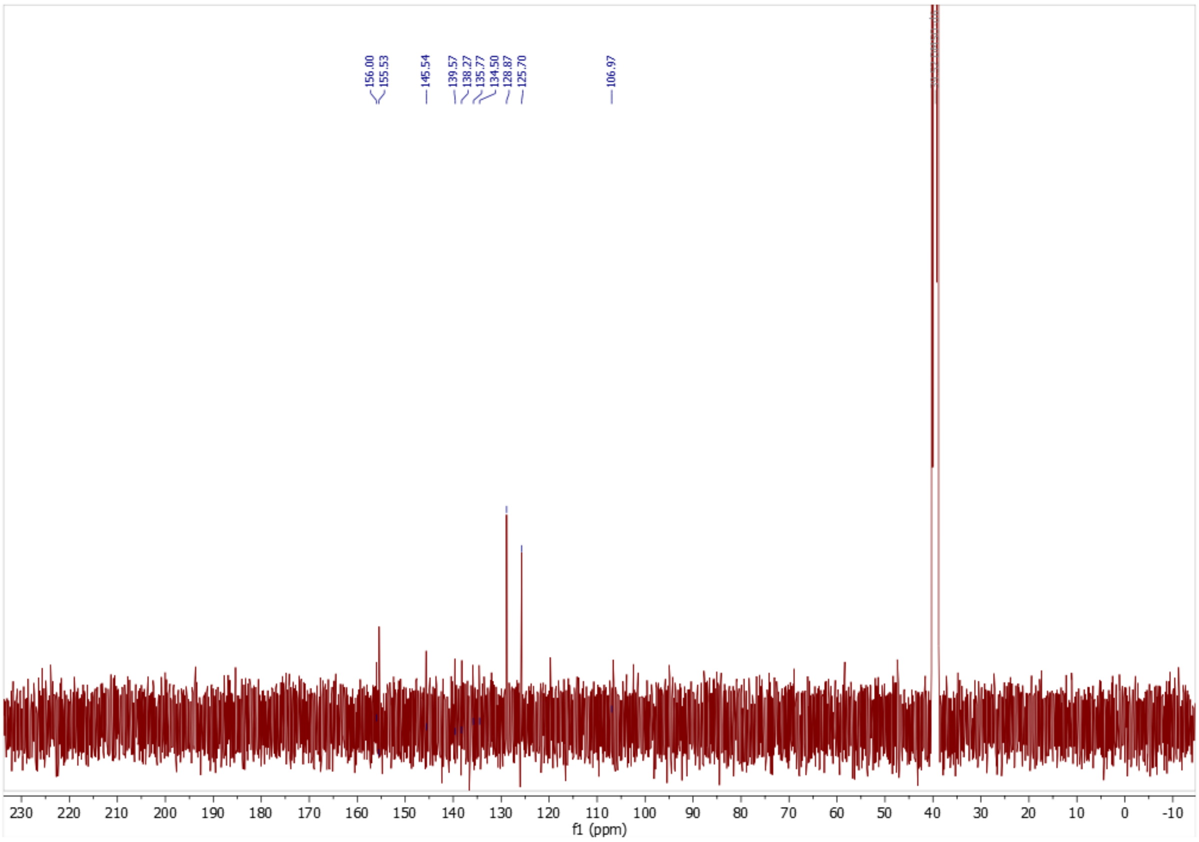
156.00
155.53
145.54
139.57
138.27
135.77
134.50
128.87
125.70
106.97
230 220 210 200 190 180 170 160 150 140 130 120 110 100 90 80 70 60 50 40 30 20 10 0 -10
f1 (ppm)

29a

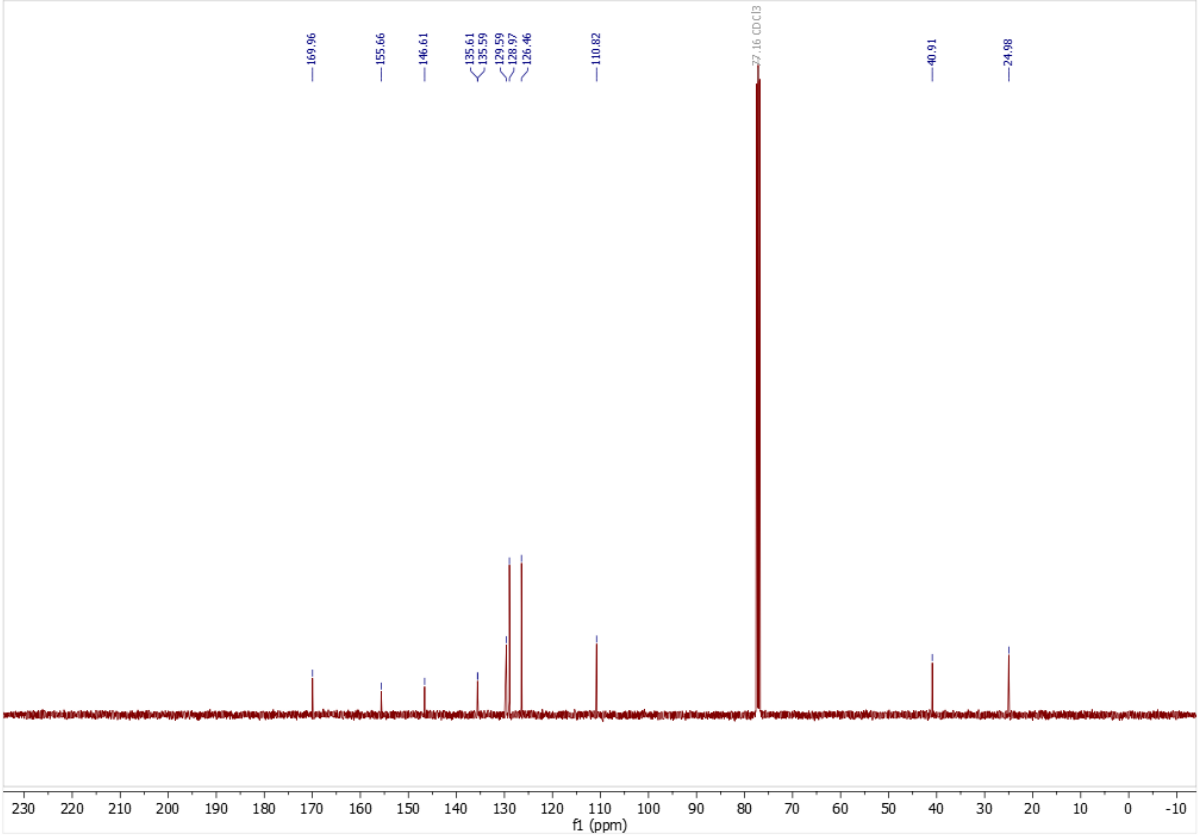
169.96
155.66
146.61
135.61
135.59
129.59
128.97
126.46
110.82
77.16 CDCl3
40.91
24.98
230 220 210 200 190 180 170 160 150 140 130 120 110 100 90 80 70 60 50 40 30 20 10 0 -10
f1 (ppm)

29b

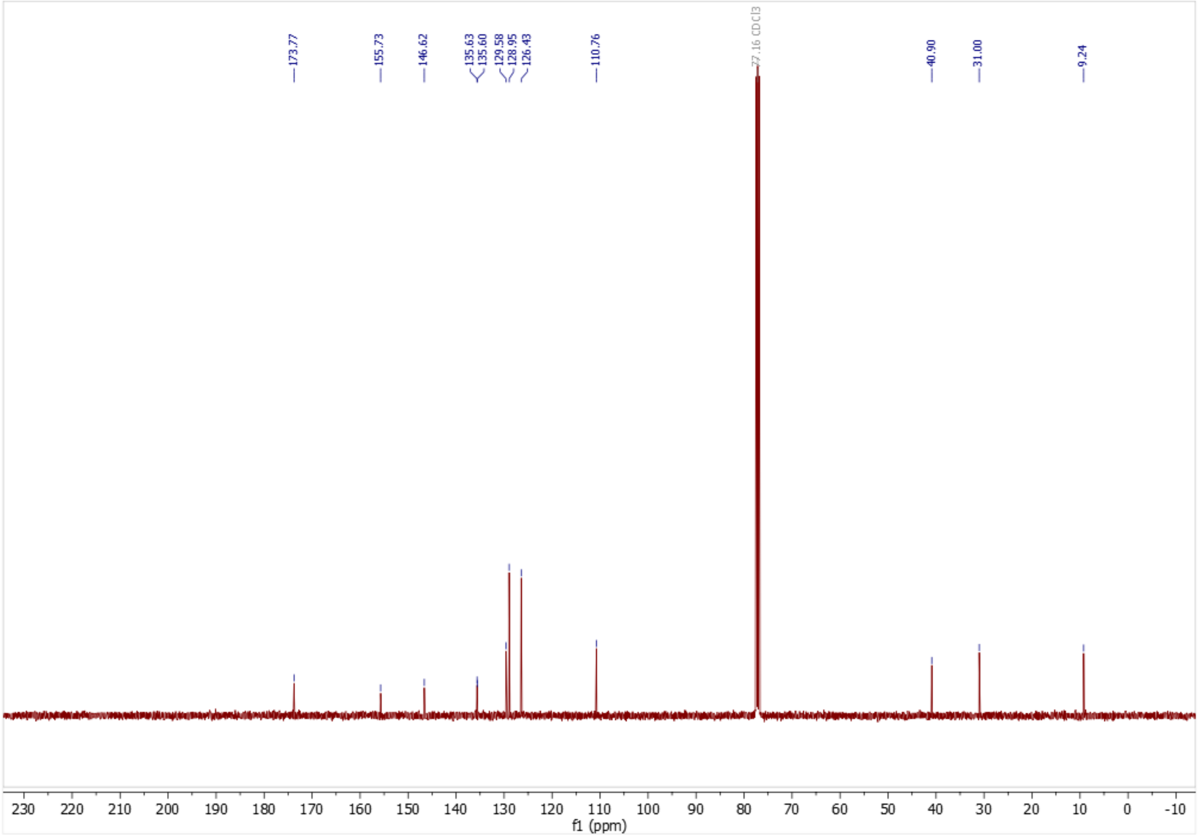
173.77
155.73
146.62
135.63
135.60
129.58
128.95
126.43
110.76
77.16 CDCl3
40.90
31.00
9.24
230 220 210 200 190 180 170 160 150 140 130 120 110 100 90 80 70 60 50 40 30 20 10 0 -10
f1 (ppm)

29c

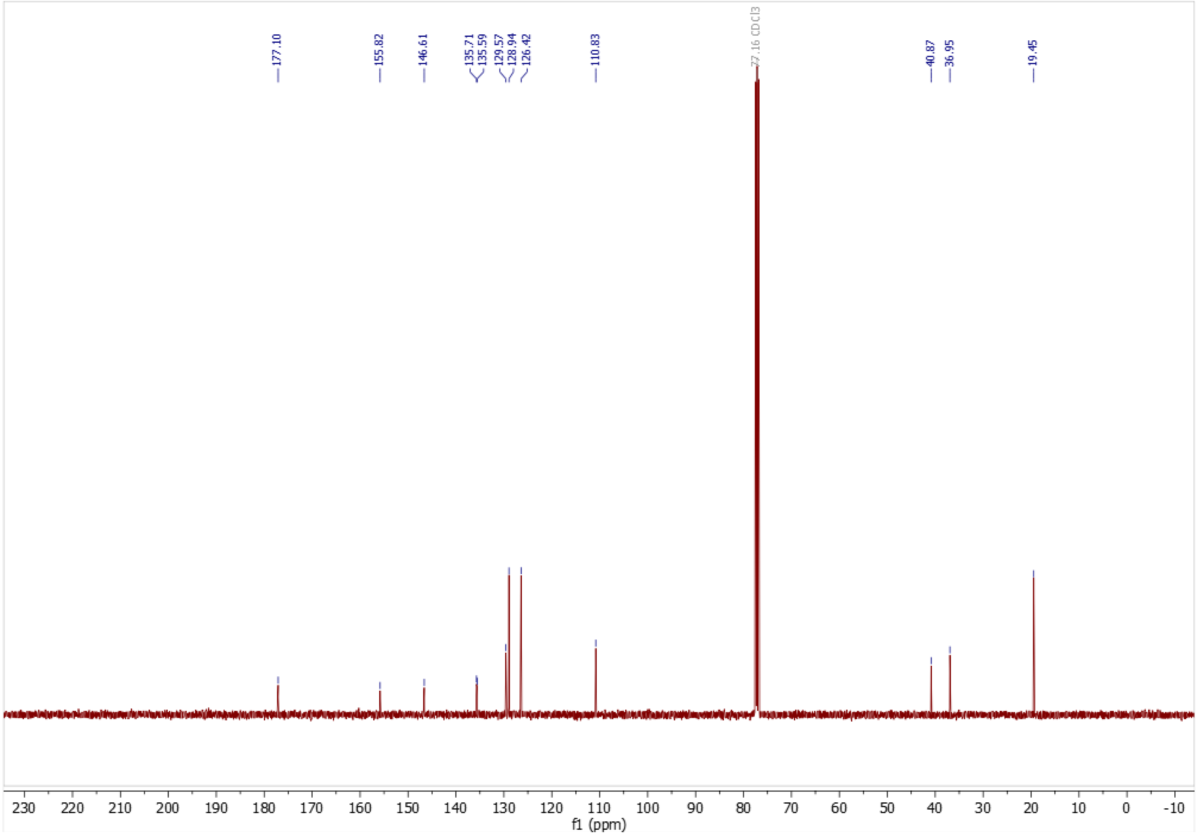
177.10
155.82
146.61
135.71
135.59
129.57
128.94
126.42
110.83
77.16 CDCl3
40.87
36.95
19.45
230 220 210 200 190 180 170 160 150 140 130 120 110 100 90 80 70 60 50 40 30 20 10 0 -10
f1 (ppm)

30b

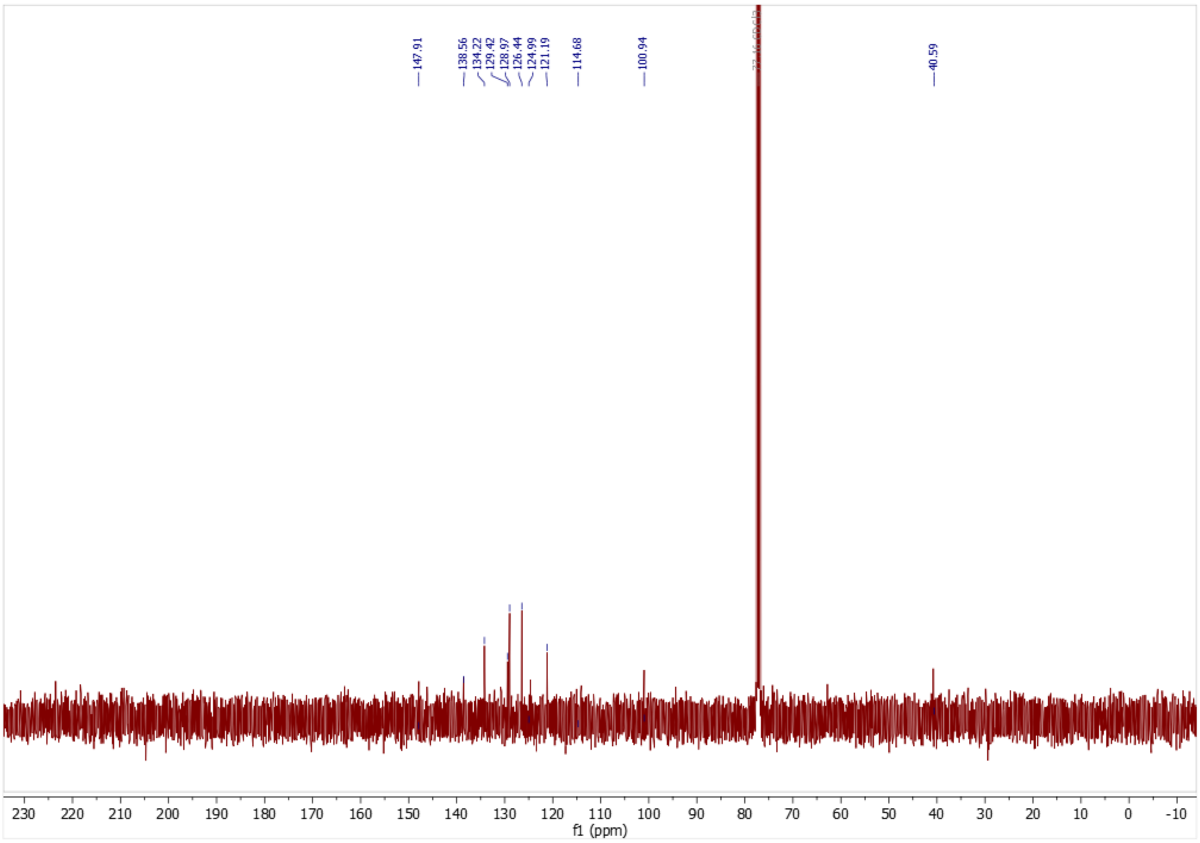
147.91
138.56
134.22
129.42
128.97
126.44
124.99
121.19
114.68
100.94
40.59
230 220 210 200 190 180 170 160 150 140 130 120 110 100 90 80 70 60 50 40 30 20 10 0 -10
f1 (ppm)

35

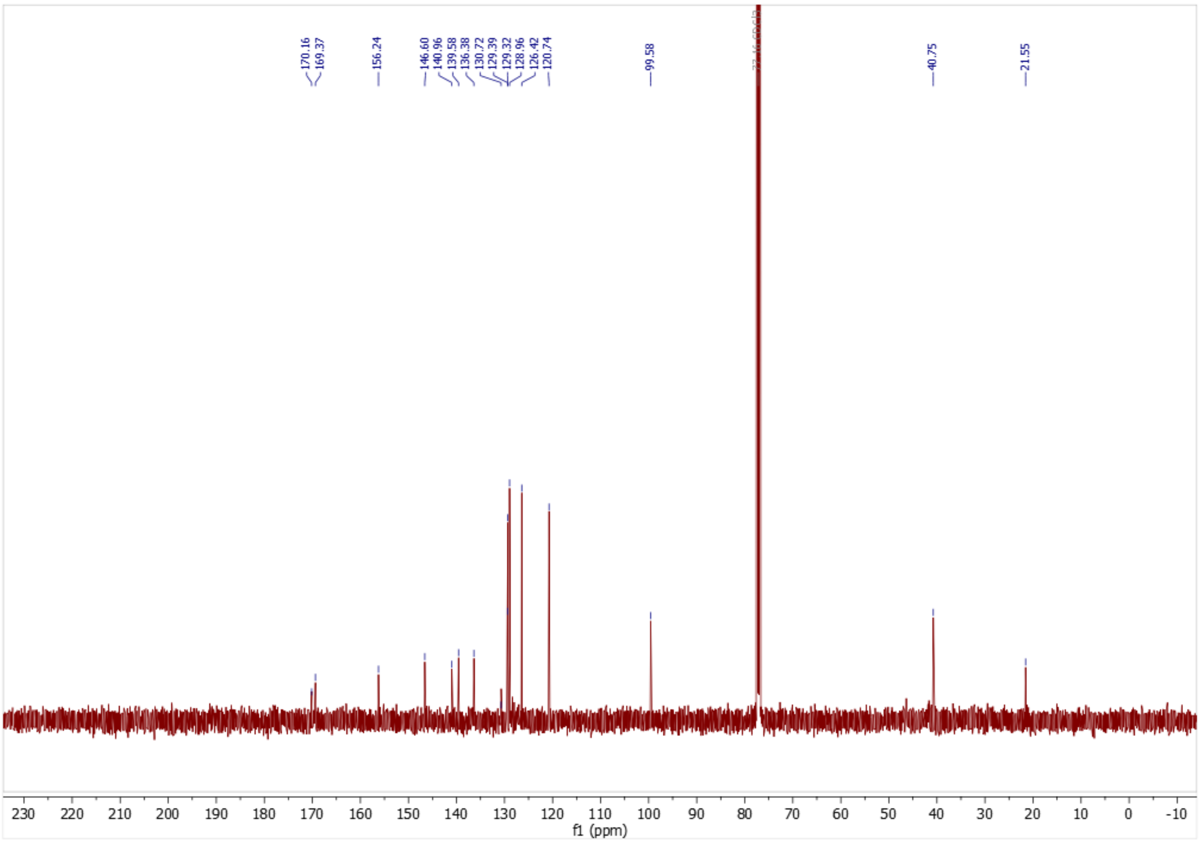
170.16
169.37
156.24
146.60
140.96
139.58
136.38
130.72
129.39
129.32
128.96
126.42
120.74
99.58
40.75
21.55
230 220 210 200 190 180 170 160 150 140 130 120 110 100 90 80 70 60 50 40 30 20 10 0 -10
f1 (ppm)

42a

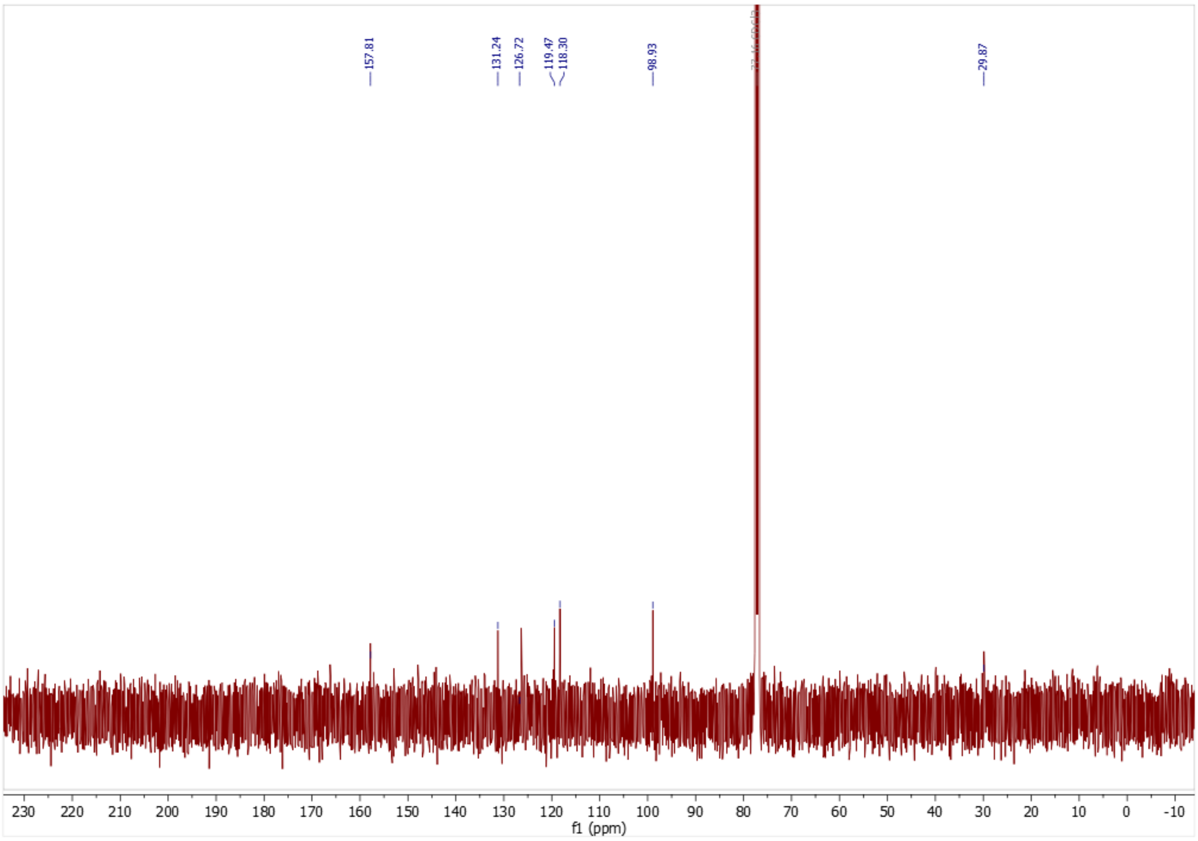
157.81
131.24
126.72
119.47
118.30
98.93
29.87
230 220 210 200 190 180 170 160 150 140 130 120 110 100 90 80 70 60 50 40 30 20 10 0 -10
f1 (ppm)

51

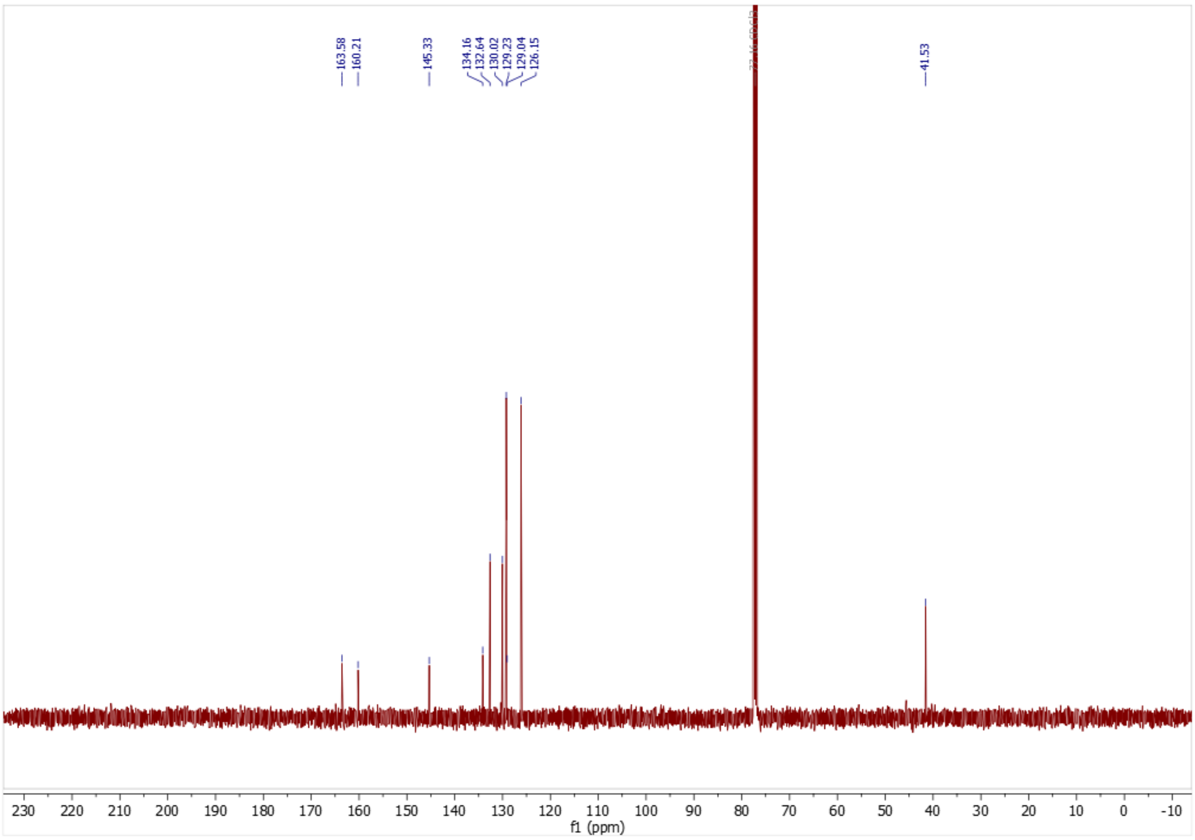
163.58
160.21
145.33
134.16
132.64
130.02
129.23
129.04
126.15
41.53
230 220 210 200 190 180 170 160 150 140 130 120 110 100 90 80 70 60 50 40 30 20 10 0 -10
f1 (ppm)

54

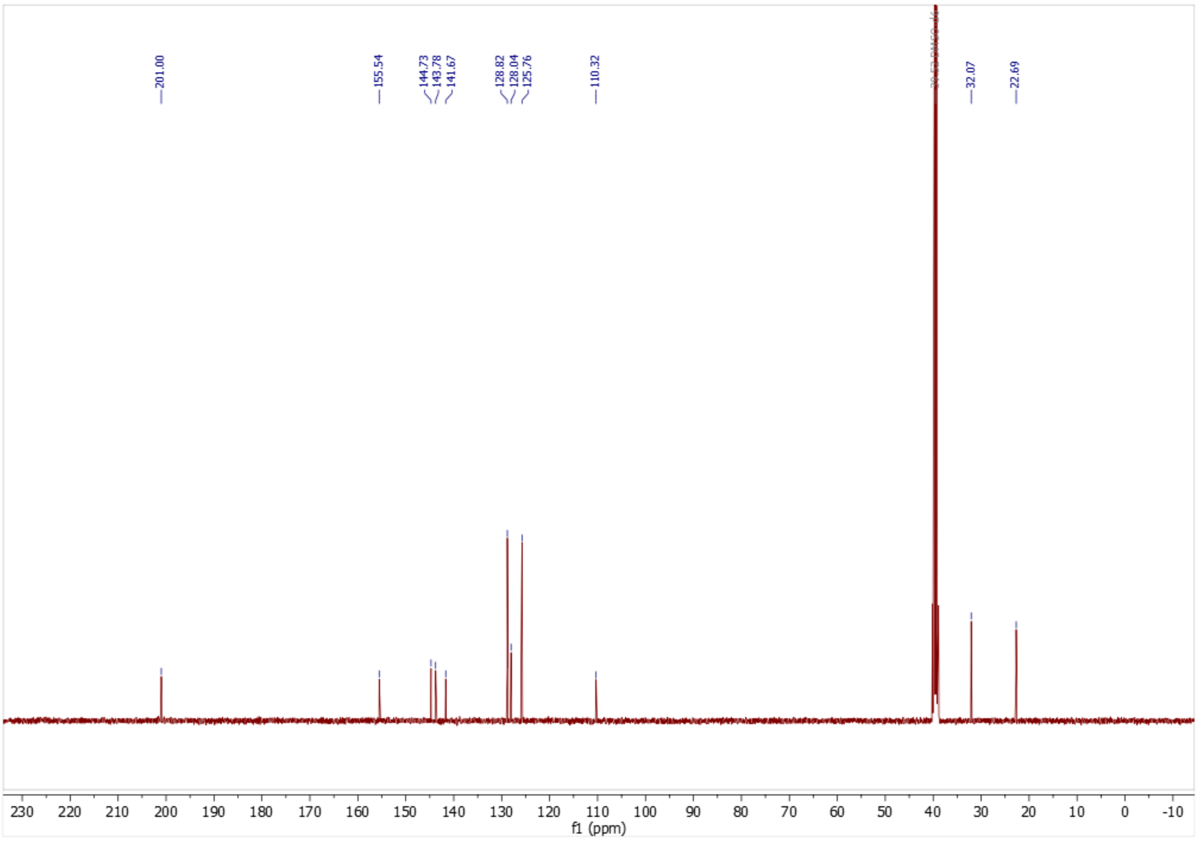
201.00
155.54
144.73
143.78
141.67
128.82
128.04
125.76
110.32
32.07
22.69
230 220 210 200 190 180 170 160 150 140 130 120 110 100 90 80 70 60 50 40 30 20 10 0 -10
f1 (ppm)

55

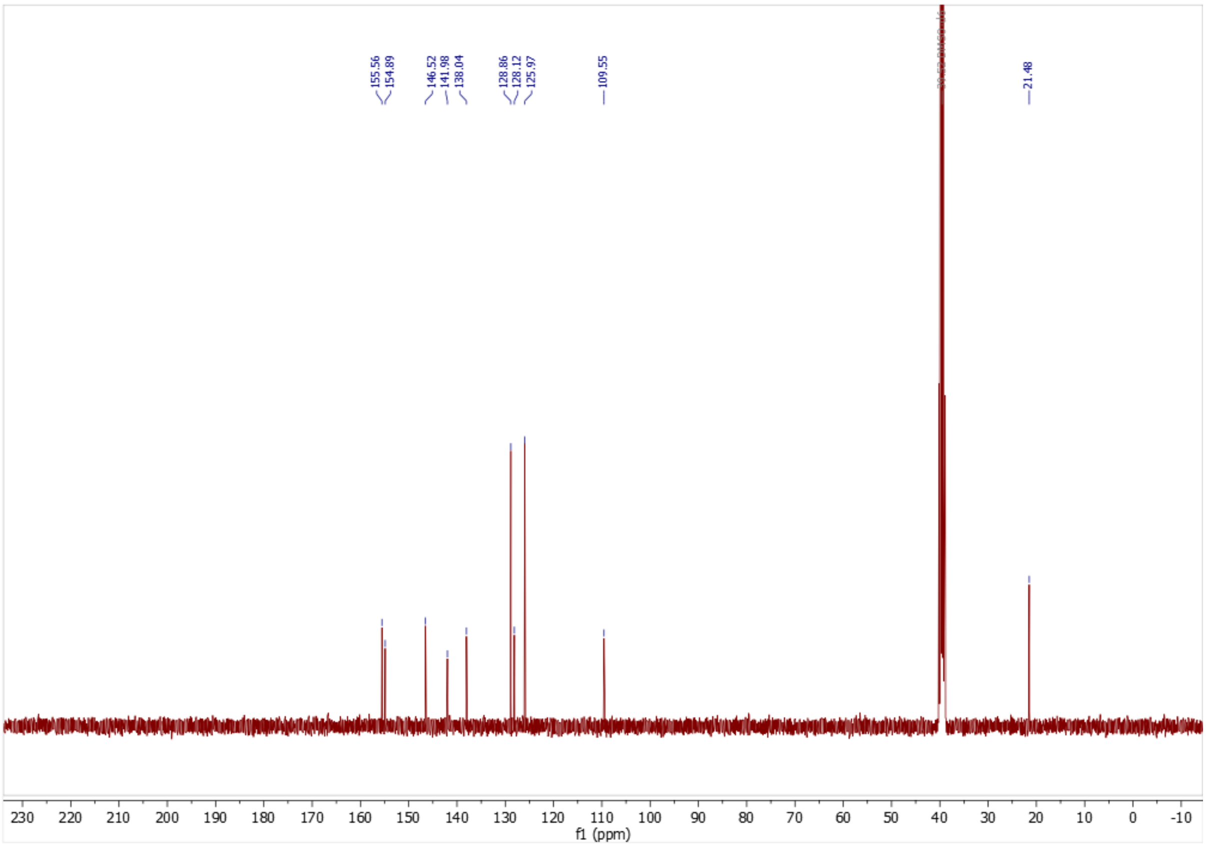
155.56
154.89
146.52
141.98
138.04
128.86
128.12
125.97
109.55
21.48
230 220 210 200 190 180 170 160 150 140 130 120 110 100 90 80 70 60 50 40 30 20 10 0 -10
f1 (ppm)

57

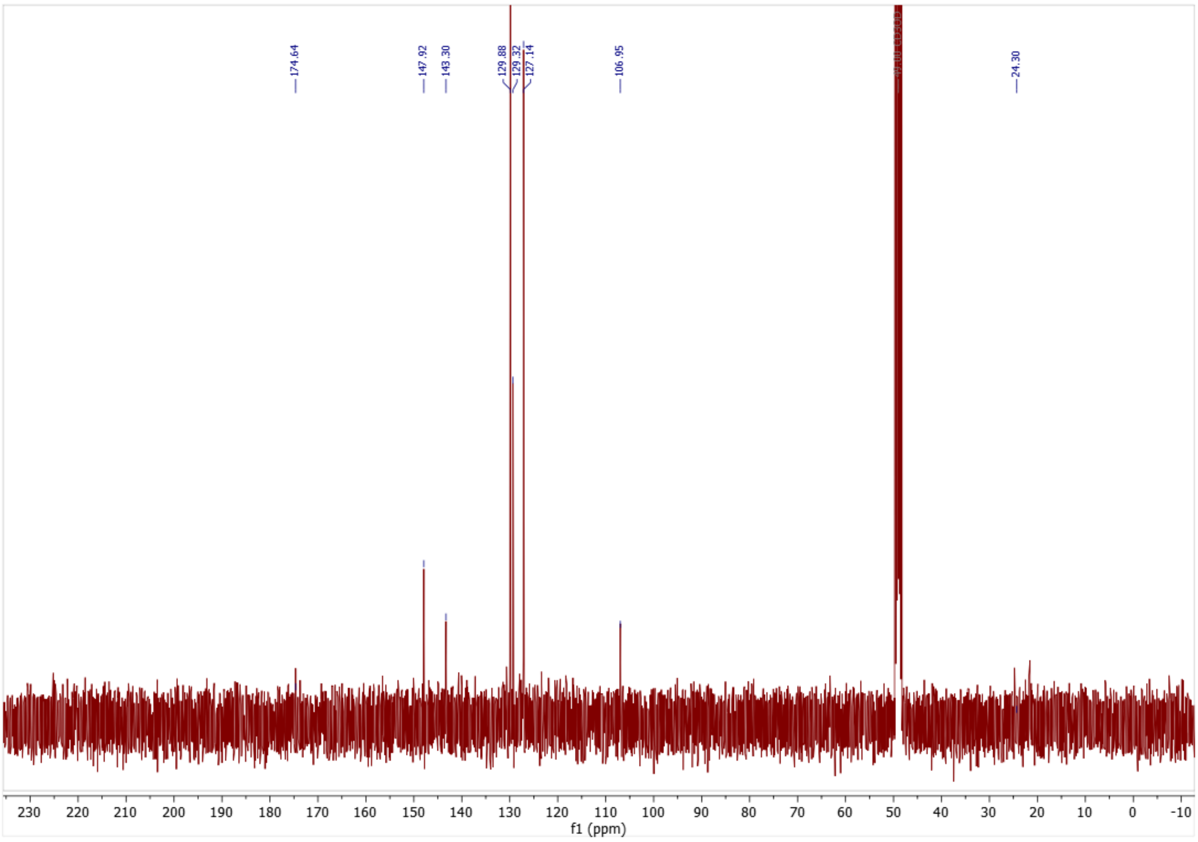
174.64
147.92
143.30
129.88
129.32
127.14
106.95
24.30
230 220 210 200 190 180 170 160 150 140 130 120 110 100 90 80 70 60 50 40 30 20 10 0 -10
f1 (ppm)

Mass spectra

4a

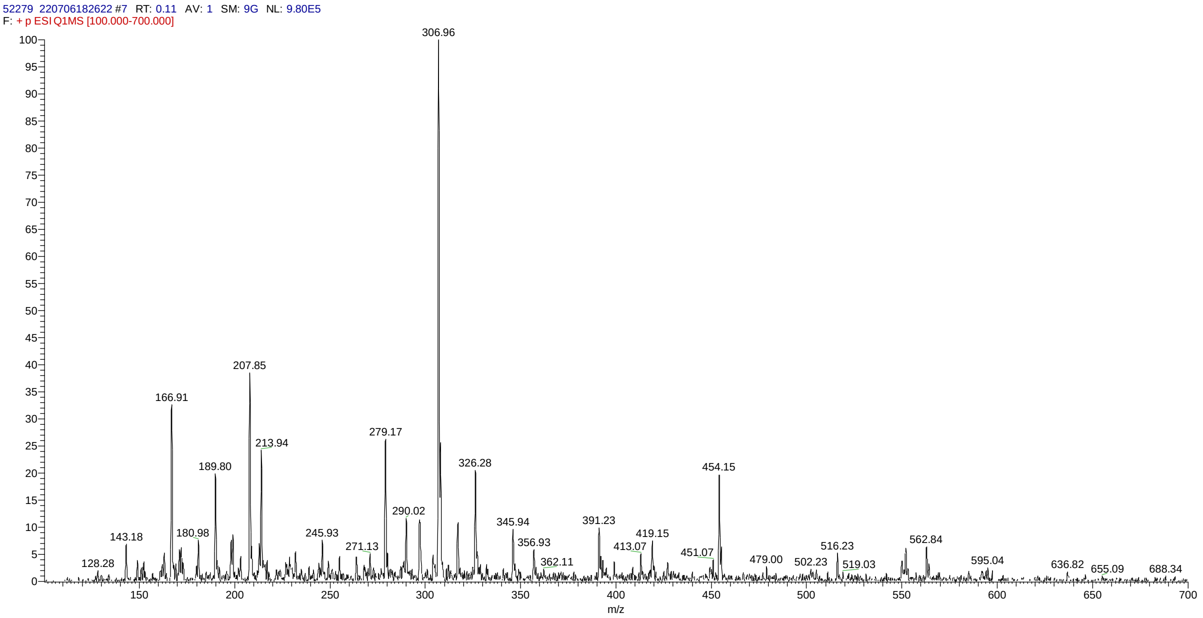

4b

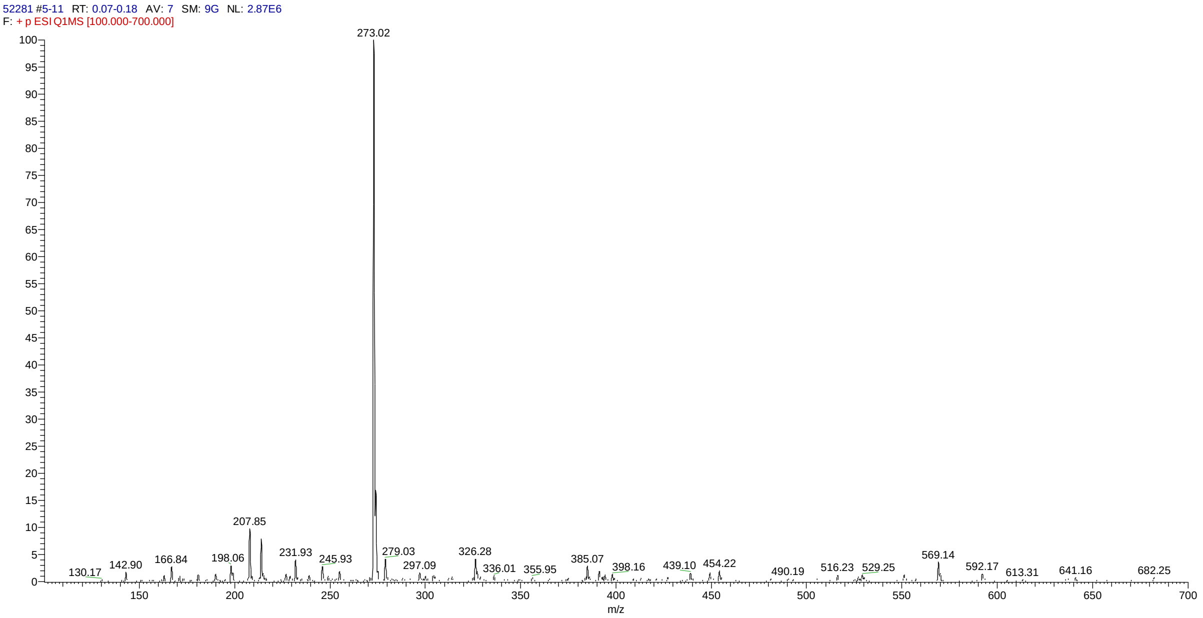

6

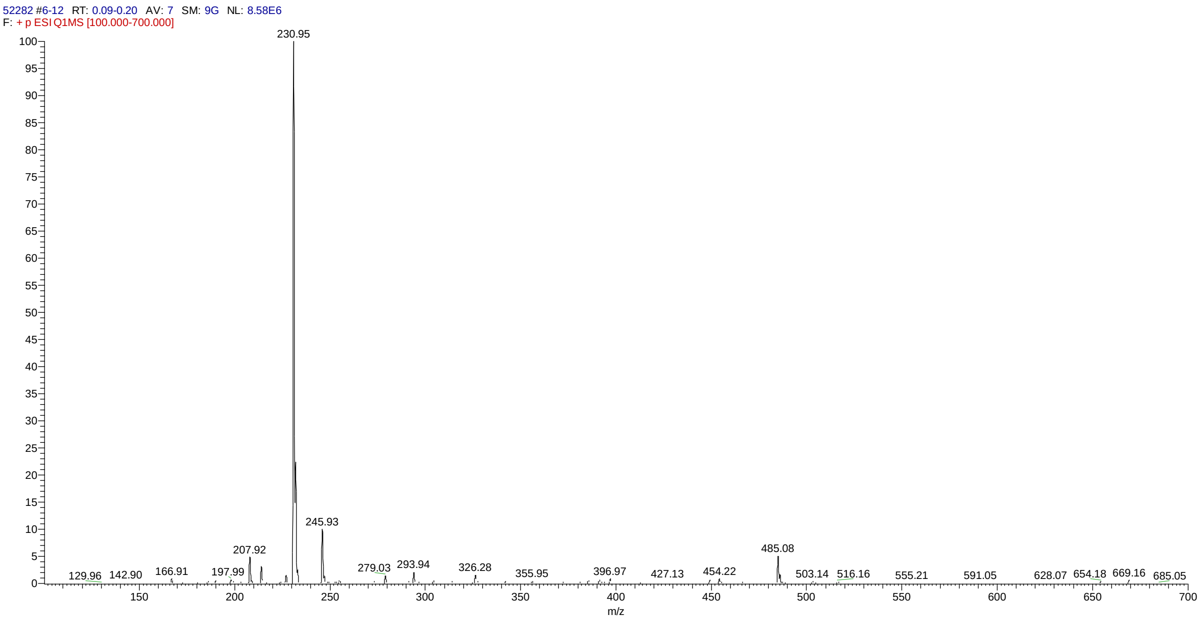
52282 #6-12 RT: 0.09-0.20 AV: 7 SM: 9G NL: 8.58E6
F: + p ESI Q1MS [100.000-700.000]
230.95
245.93
207.92
485.08
129.96 142.90 166.91 197.99 279.03 293.94 326.28 355.95 396.97 427.13 454.22 503.14 516.16 555.21 591.05 628.07 654.18 669.16 685.05
m/z

7

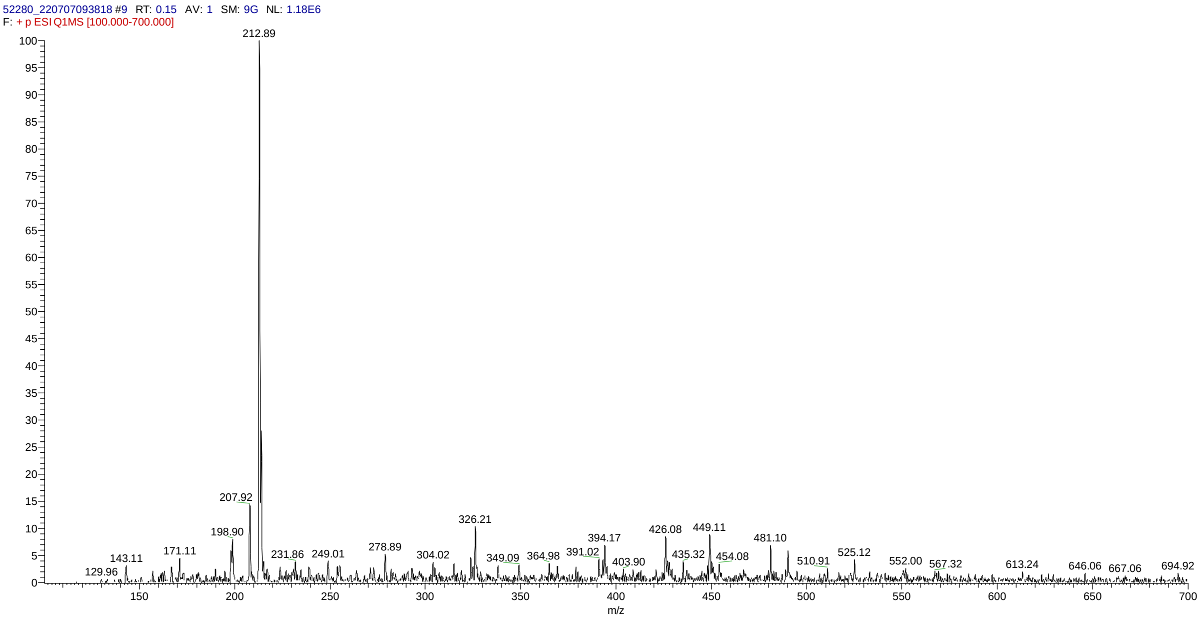
52280_220707093818 #9 RT: 0.15 AV: 1 SM: 9G NL: 1.18E6
F: + p ESI Q1MS [100.000-700.000]
212.89
207.92
198.90
326.21
143.11 171.11 129.96 231.86 249.01 278.89 304.02 349.09 364.98 391.02 394.17 403.90 426.08 435.32 449.11 454.08 481.10 510.91 525.12 552.00 567.32 613.24 646.06 667.06 694.92
m/z

16

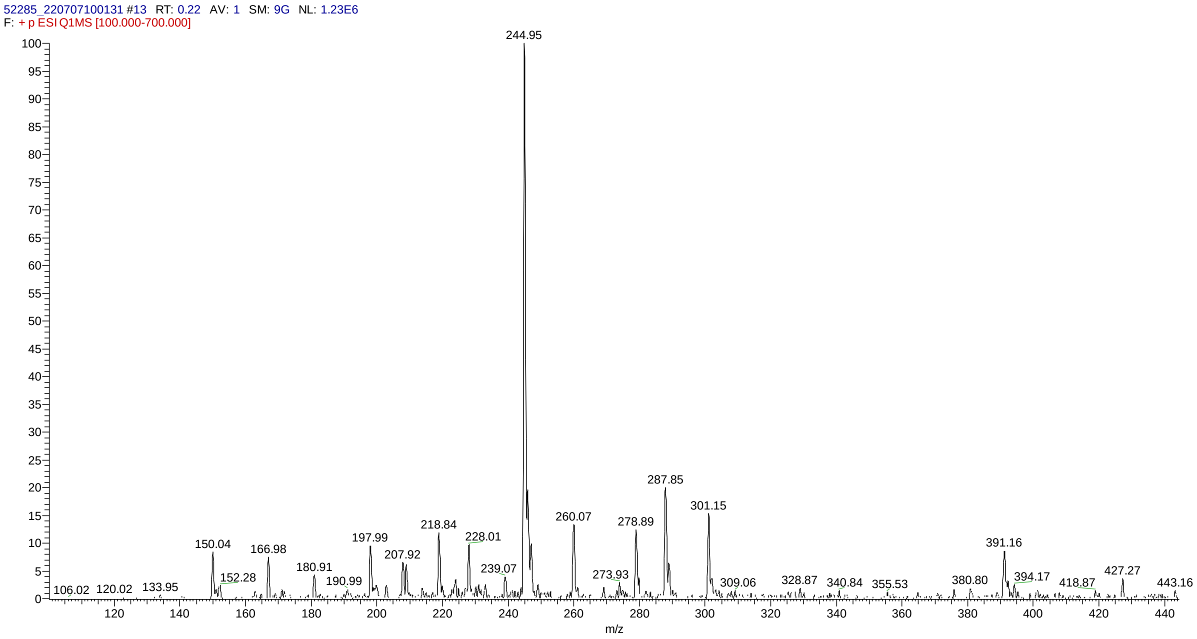
52285_220707100131 #13 RT: 0.22 AV: 1 SM: 9G NL: 1.23E6
F: + p ESI Q1MS [100.000-700.000]
244.95
287.85
301.15
260.07
278.89
218.84
228.01
197.99
207.92
150.04
166.98
180.91
391.16
427.27
239.07
273.93
m/z

17

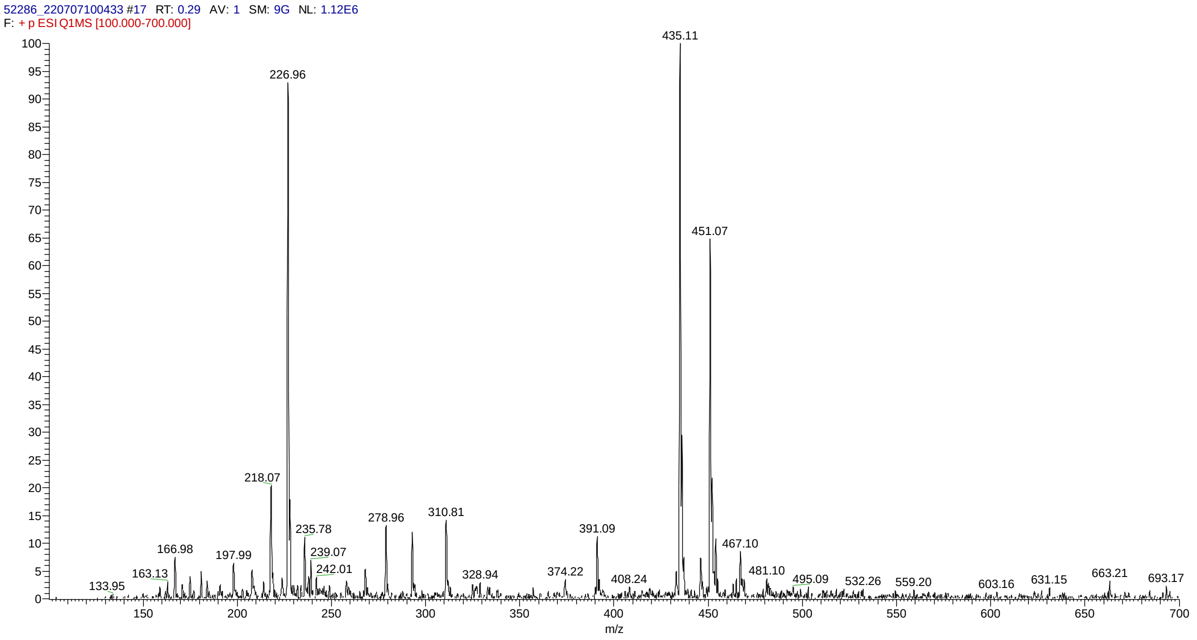
52286_220707100433 #17 RT: 0.29 AV: 1 SM: 9G NL: 1.12E6
F: + p ESI Q1MS [100.000-700.000]
435.11
226.96
451.07
218.07
310.81
278.96
235.78
391.09
467.10
166.98
197.99
239.07
242.01
663.21
693.17
m/z

22

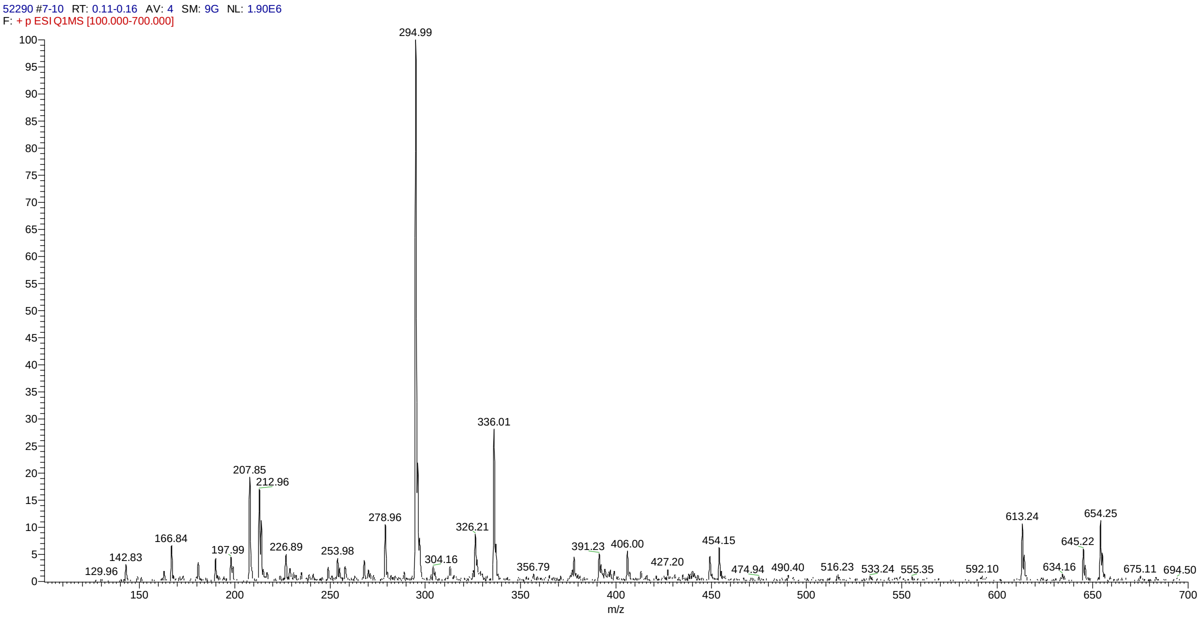
52290 #7-10 RT: 0.11-0.16 AV: 4 SM: 9G NL: 1.90E6
F: + p ESI Q1MS [100.000-700.000]
294.99
336.01
207.85
212.96
278.96
326.21
166.84
654.25
613.24
645.22
142.83
197.99
226.89
253.98
304.16
391.23
406.00
454.15
427.20
129.96
356.79
474.94
490.40
516.23
533.24
555.35
592.10
634.16
675.11
694.50
m/z

25a

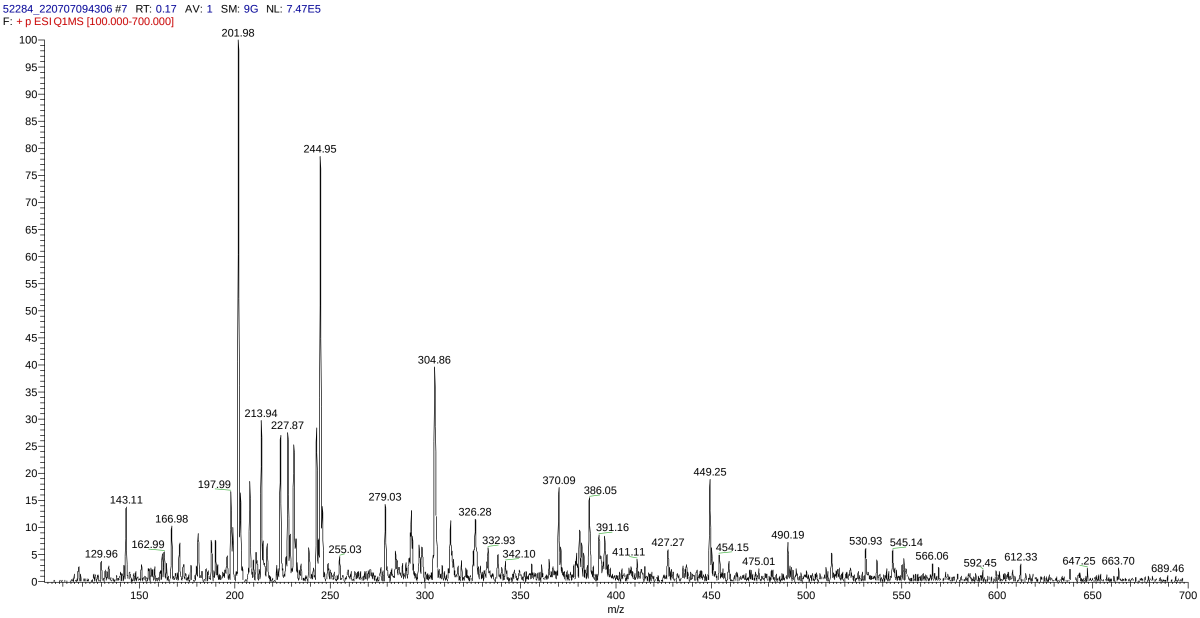
52284_220707094306 #7 RT: 0.17 AV: 1 SM: 9G NL: 7.47E5
F: + p ESI Q1MS [100.000-700.000]
201.98
244.95
304.86
213.94
227.87
197.99
279.03
370.09
386.05
449.25
143.11
166.98
326.28
391.16
490.19
129.96
162.99
255.03
332.93
342.10
411.11
427.27
454.15
475.01
530.93
545.14
566.06
592.45
612.33
647.25
663.70
689.46
m/z

25c

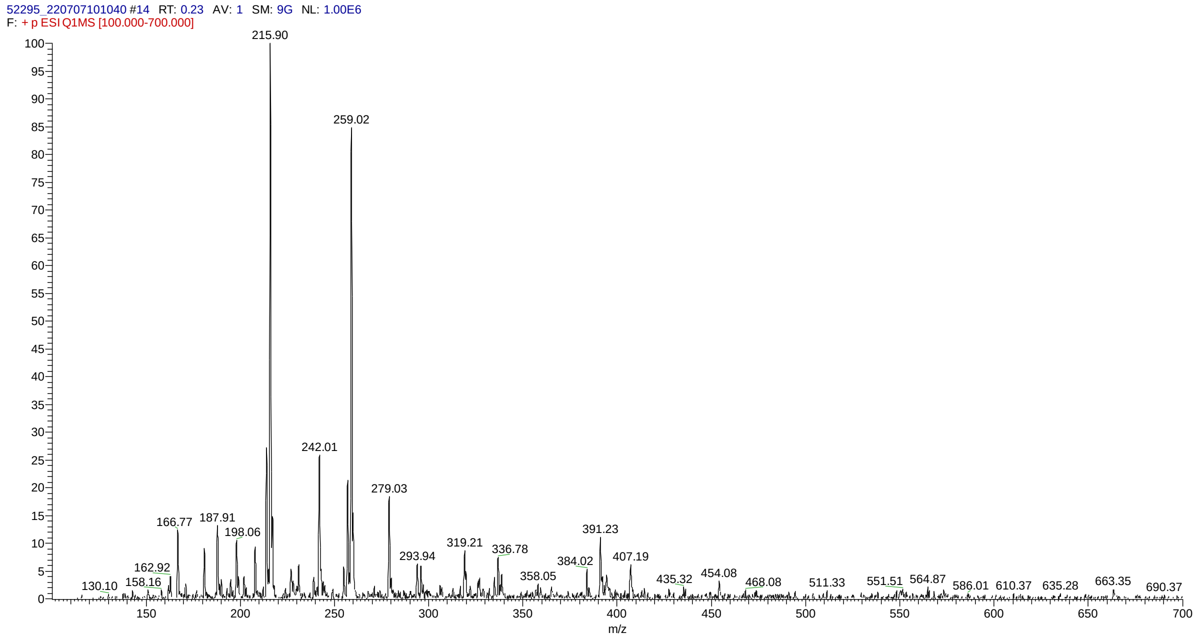
52295_220707101040 #14 RT: 0.23 AV: 1 SM: 9G NL: 1.00E6
F: + p ESI Q1MS [100.000-700.000]
215.90
259.02
242.01
279.03
166.77
187.91
198.06
162.92
158.16
130.10
293.94
319.21
336.78
358.05
384.02
391.23
407.19
435.32
454.08
468.08
511.33
551.51
564.87
586.01
610.37
635.28
663.35
690.37
m/z

25d

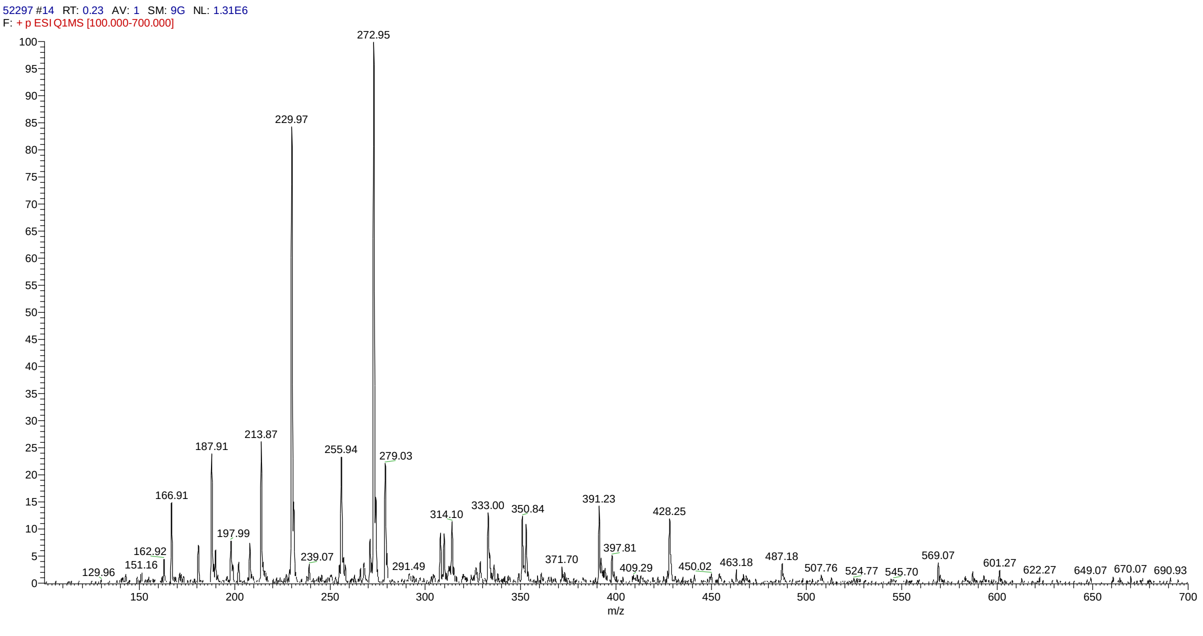
52297 #14 RT: 0.23 AV: 1 SM: 9G NL: 1.31E6
F: + p ESI Q1MS [100.000-700.000]
272.95
229.97
213.87
187.91
255.94
279.03
166.91
197.99
162.92
151.16
129.96
239.07
291.49
314.10
333.00
350.84
371.70
391.23
397.81
409.29
428.25
450.02
463.18
487.18
507.76
524.77
545.70
569.07
601.27
622.27
649.07
670.07
690.93
m/z

25e

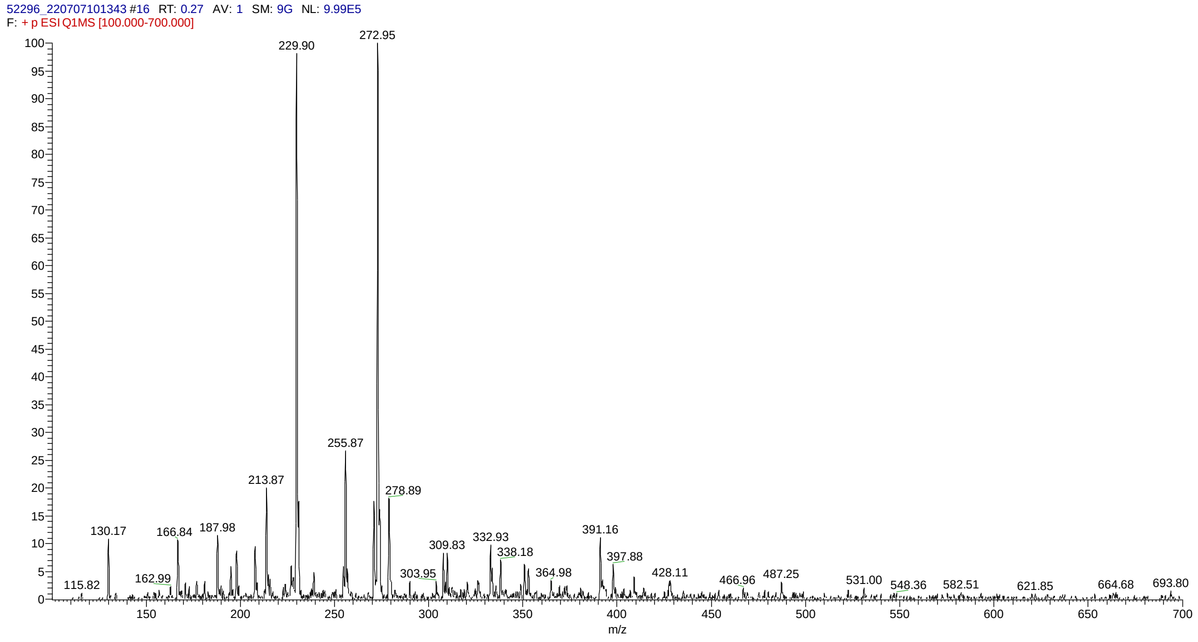
52296_220707101343 #16 RT: 0.27 AV: 1 SM: 9G NL: 9.99E5
F: + p ESI Q1MS [100.000-700.000]
272.95
229.90
255.87
213.87
278.89
130.17
166.84
187.98
391.16
309.83
332.93
338.18
397.88
115.82
162.99
303.95
364.98
428.11
466.96
487.25
531.00
548.36
582.51
621.85
664.68
693.80
m/z

25f

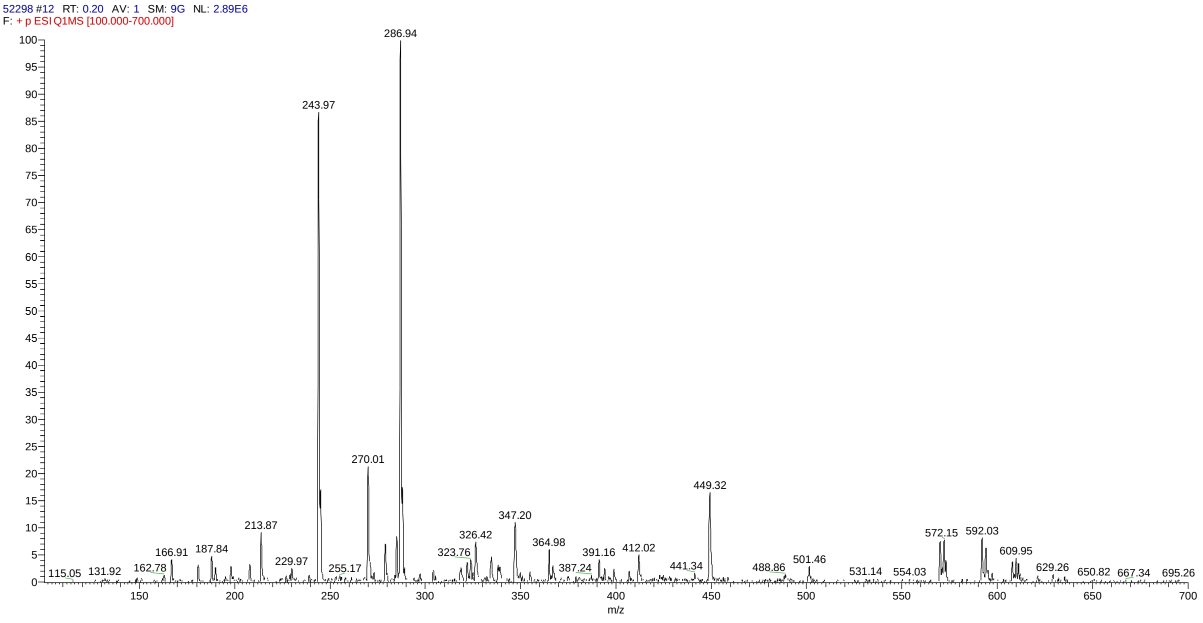
52298 #12 RT: 0.20 AV: 1 SM: 9G NL: 2.89E6
F: + p ESI Q1MS [100.000-700.000]
286.94
243.97
270.01
449.32
347.20
213.87
326.42
572.15
592.03
166.91
187.84
364.98
412.02
609.95
323.76
391.16
501.46
229.97
441.34
115.05
131.92
162.78
255.17
387.24
488.86
531.14
554.03
629.26
650.82
667.34
695.26
m/z

27

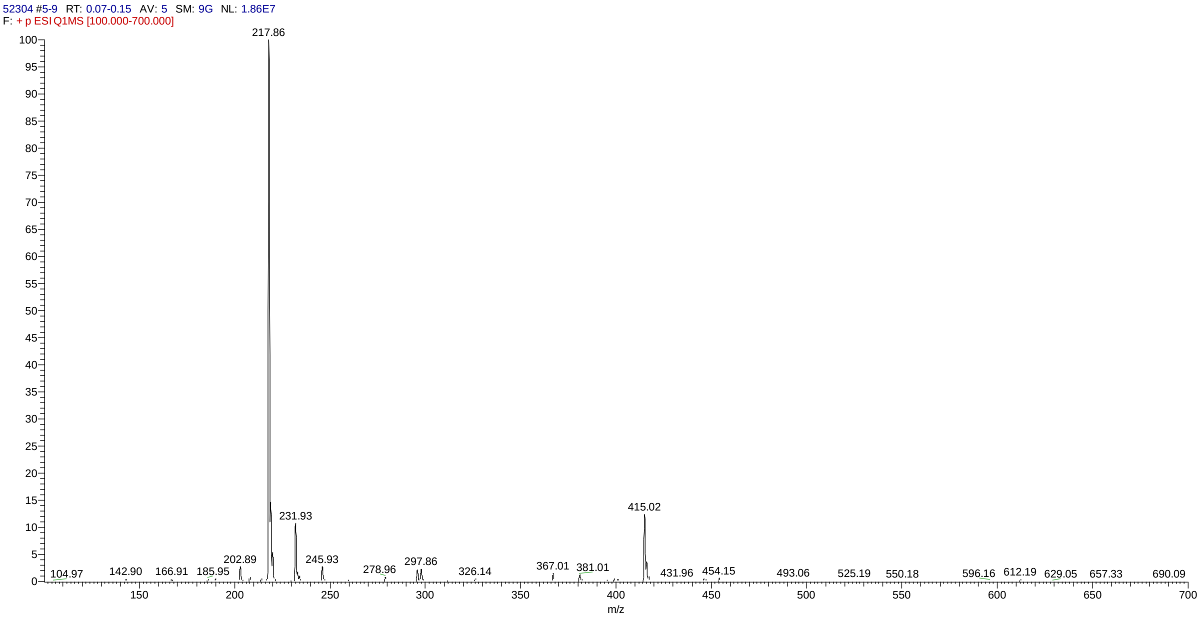
52304 #5-9 RT: 0.07-0.15 AV: 5 SM: 9G NL: 1.86E7
F: + p ESI Q1MS [100.000-700.000]
217.86
231.93
415.02
245.93
202.89
297.86
104.97
142.90
166.91
185.95
278.96
326.14
367.01
381.01
431.96
454.15
493.06
525.19
550.18
596.16
612.19
629.05
657.33
690.09
m/z

28

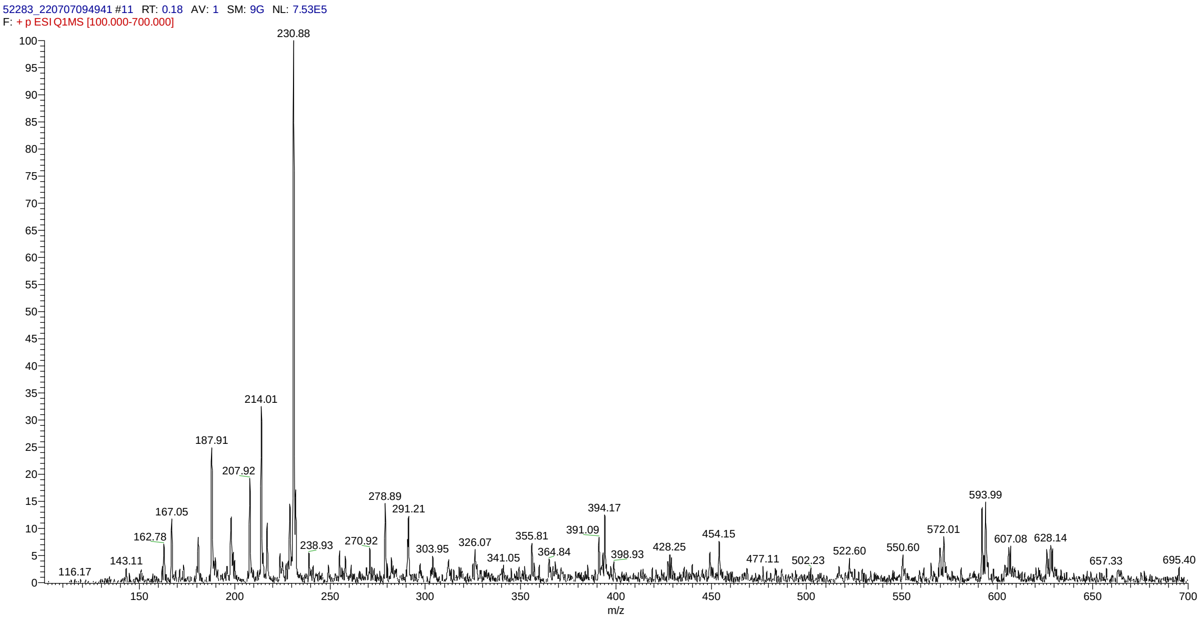
52283_220707094941 #11 RT: 0.18 AV: 1 SM: 9G NL: 7.53E5
F: + p ESI Q1MS [100.000-700.000]
230.88
214.01
187.91
207.92
167.05
162.78
143.11
116.17
278.89
291.21
238.93
270.92
303.95
326.07
341.05
355.81
364.84
391.09
394.17
398.93
428.25
454.15
477.11
502.23
522.60
550.60
572.01
593.99
607.08
628.14
657.33
695.40
m/z

29a

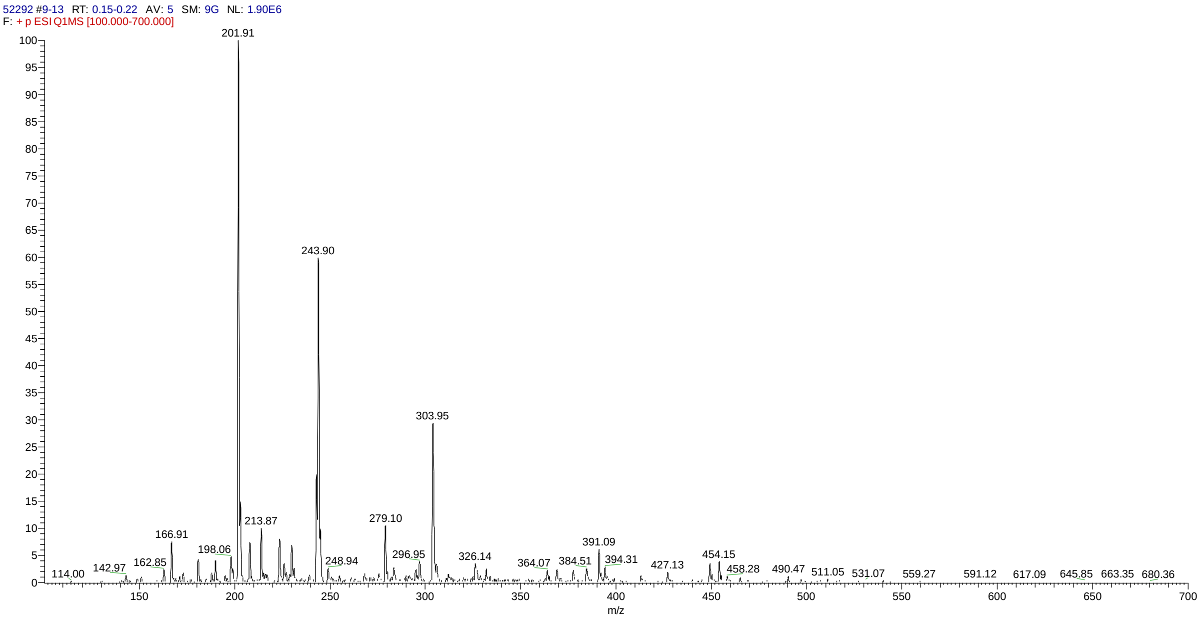
52292 #9-13 RT: 0.15-0.22 AV: 5 SM: 9G NL: 1.90E6
F: + p ESI Q1MS [100.000-700.000]
201.91
243.90
303.95
279.10
213.87
166.91
198.06
391.09
394.31
454.15
m/z

29b

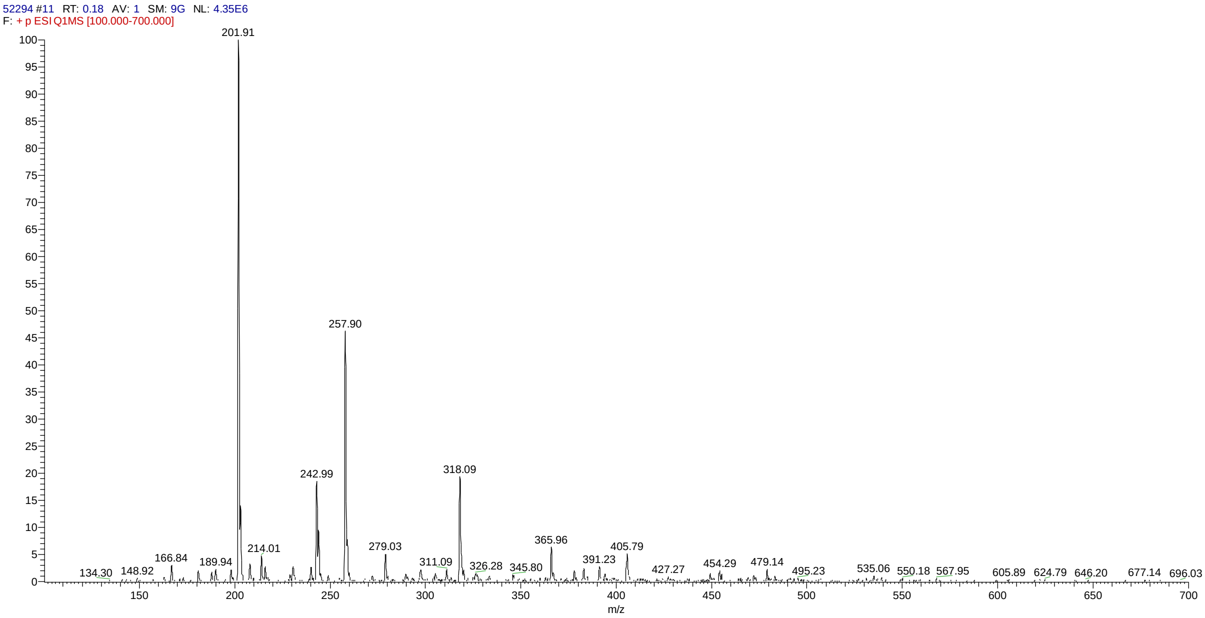
52294 #11 RT: 0.18 AV: 1 SM: 9G NL: 4.35E6
F: + p ESI Q1MS [100.000-700.000]
201.91
257.90
242.99
318.09
365.96
405.79
279.03
214.01
166.84
m/z

29c

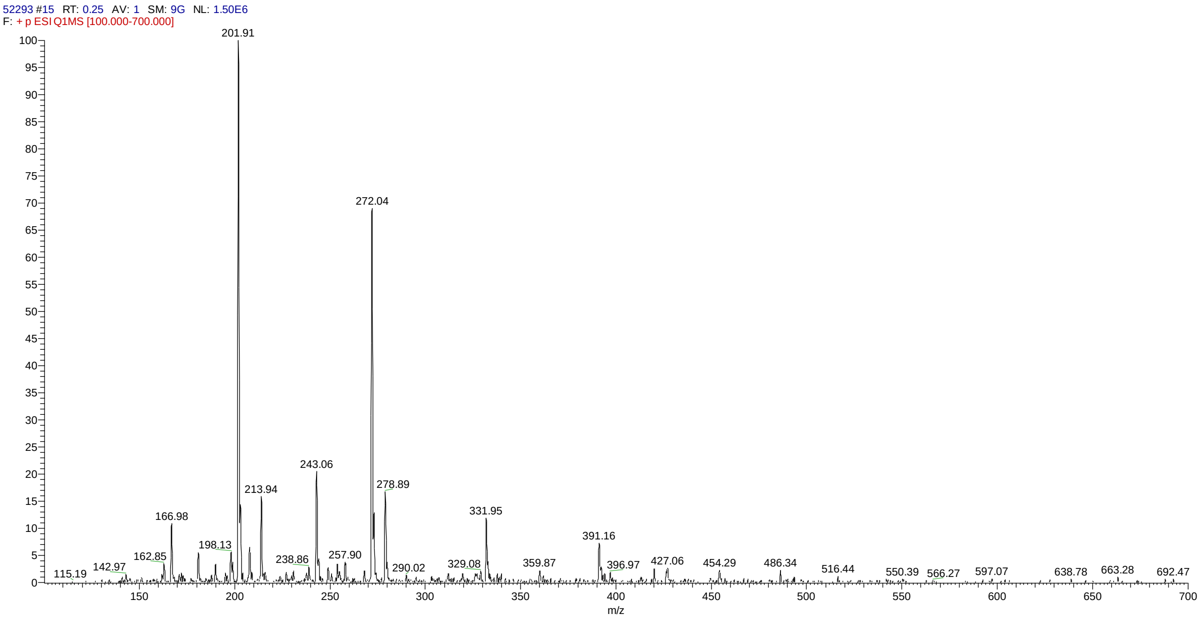
52293 #15 RT: 0.25 AV: 1 SM: 9G NL: 1.50E6
F: + p ESI Q1MS [100.000-700.000]
201.91
272.04
243.06
278.89
213.94
331.95
166.98
391.16
198.13
162.85
238.86
257.90
142.97
115.19
290.02
329.08
359.87
396.97
427.06
454.29
486.34
516.44
550.39
566.27
597.07
638.78
663.28
692.47
m/z

30a

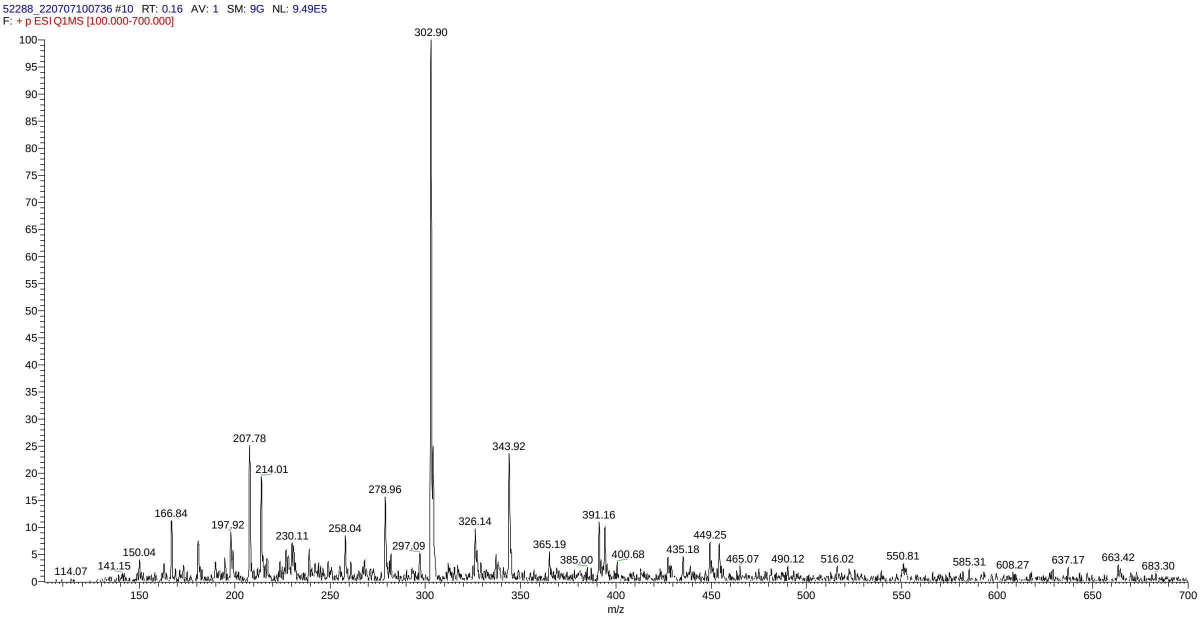
52288_220707100736 #10 RT: 0.16 AV: 1 SM: 9G NL: 9.49E5
F: + p ESI Q1MS [100.000-700.000]
302.90
207.78
343.92
214.01
278.96
166.84
197.92
230.11
258.04
326.14
391.16
150.04
141.15
114.07
297.09
365.19
385.00
400.68
435.18
449.25
465.07
490.12
516.02
550.81
585.31
608.27
637.17
663.42
683.30
m/z

30b

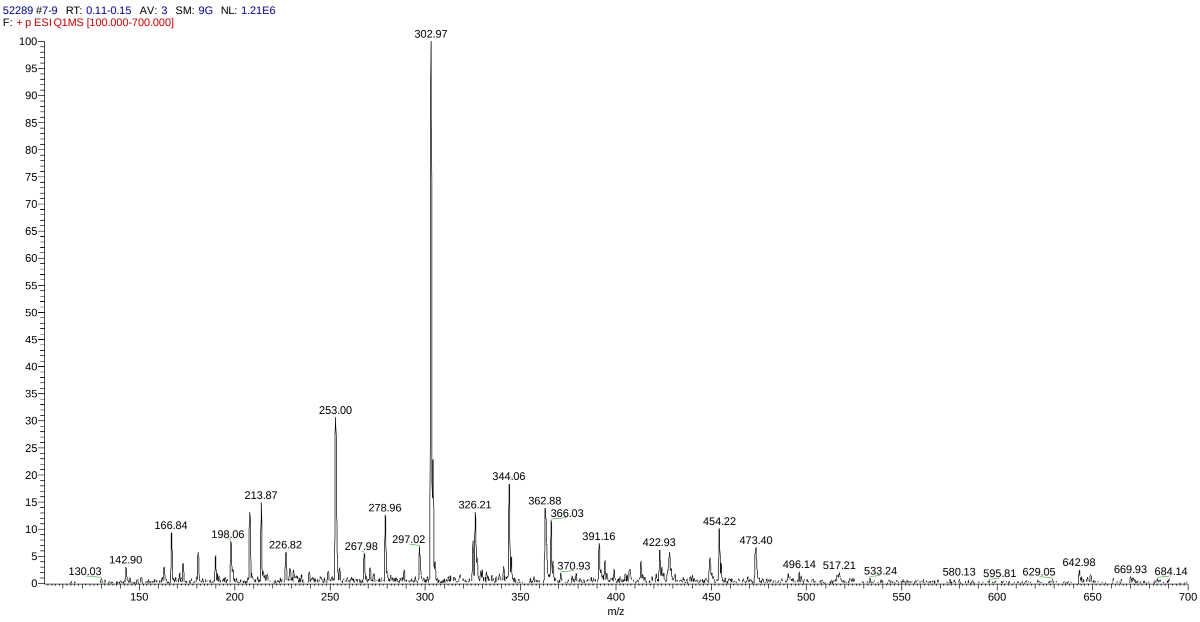
52289 #7-9 RT: 0.11-0.15 AV: 3 SM: 9G NL: 1.21E6
F: + p ESI Q1MS [100.000-700.000]
302.97
253.00
344.06
213.87
278.96
362.88
326.21
366.03
166.84
454.22
198.06
297.02
391.16
473.40
226.82
422.93
267.98
142.90
370.93
496.14
517.21
642.98
130.03
533.24
580.13
595.81
629.05
669.93
684.14
m/z

35

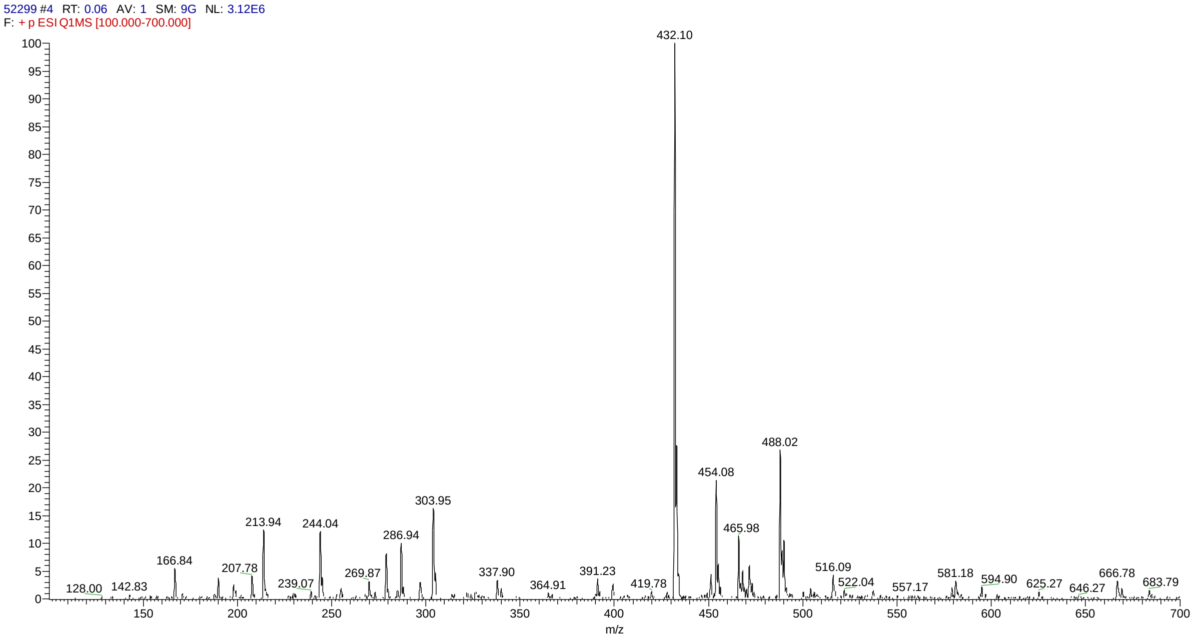
52299 #4 RT: 0.06 AV: 1 SM: 9G NL: 3.12E6
F: + p ESI Q1MS [100.000-700.000]
432.10
488.02
454.08
303.95
465.98
213.94
244.04
286.94
166.84
516.09
207.78
269.87
337.90
391.23
581.18
666.78
128.00
142.83
239.07
364.91
419.78
522.04
557.17
594.90
625.27
646.27
683.79
m/z

42a

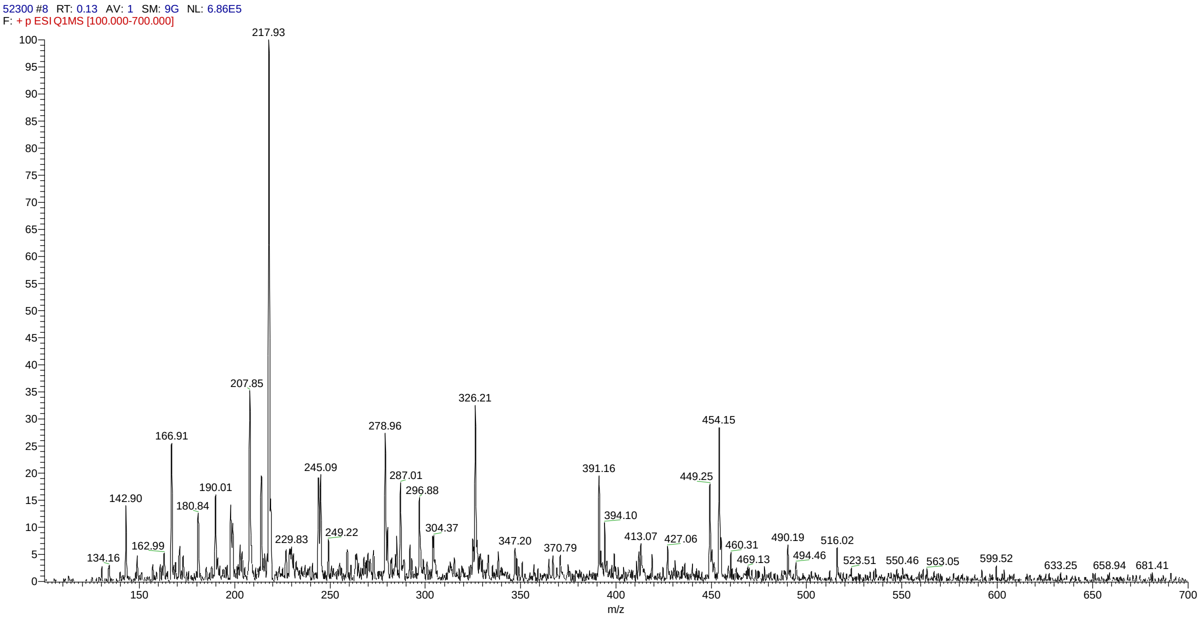
52300 #8 RT: 0.13 AV: 1 SM: 9G NL: 6.86E5
F: + p ESI Q1MS [100.000-700.000]
217.93
207.85
326.21
454.15
278.96
166.91
245.09
391.16
287.01
449.25
296.88
142.90
190.01
180.84
394.10
304.37
249.22
229.83
162.99
134.16
347.20
370.79
413.07
427.06
490.19
516.02
460.31
469.13
494.46
523.51
550.46
563.05
599.52
633.25
658.94
681.41
m/z

51

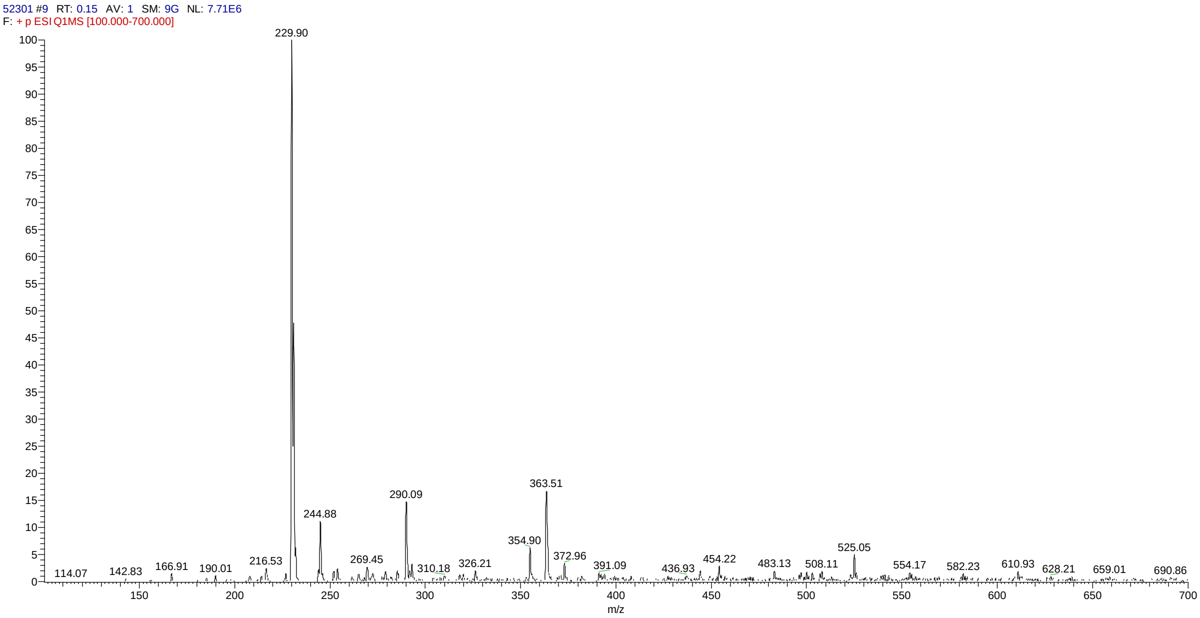
52301 #9 RT: 0.15 AV: 1 SM: 9G NL: 7.71E6
F: + p ESI Q1MS [100.000-700.000]
229.90
363.51
290.09
244.88
354.90
525.05
372.96
269.45
454.22
216.53
114.07
142.83
166.91
190.01
310.18
326.21
391.09
436.93
483.13
508.11
554.17
582.23
610.93
628.21
659.01
690.86
m/z

54

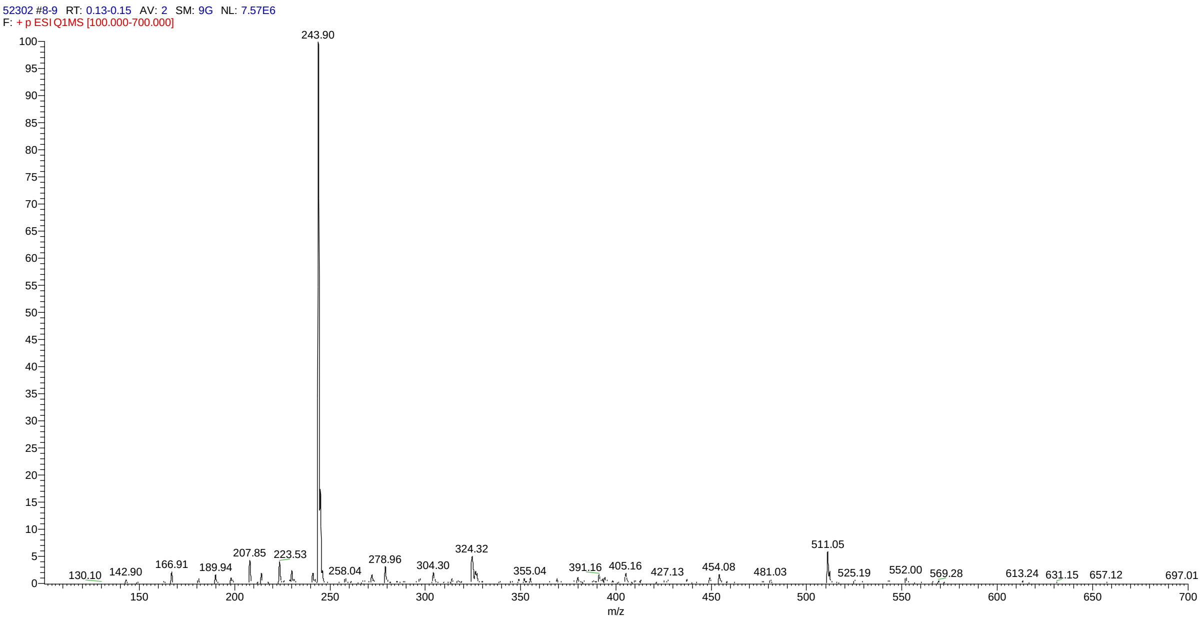
52302 #8-9 RT: 0.13-0.15 AV: 2 SM: 9G NL: 7.57E6
F: + p ESI Q1MS [100.000-700.000]
243.90
511.05
324.32
207.85
223.53
278.96
166.91
m/z

55

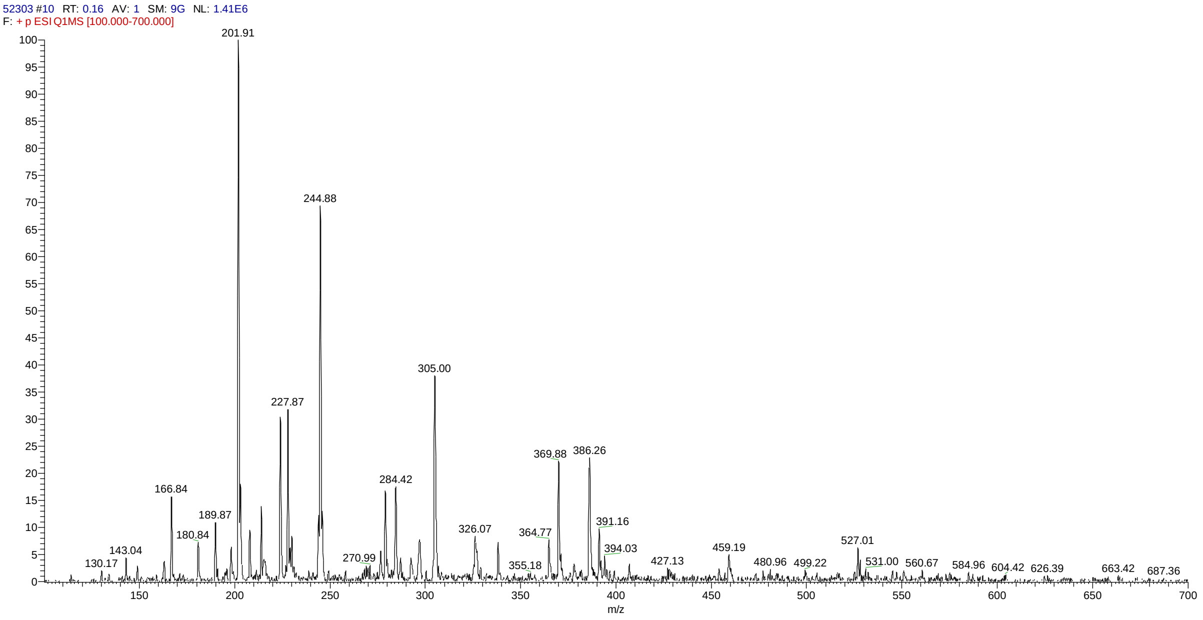
52303 #10 RT: 0.16 AV: 1 SM: 9G NL: 1.41E6
F: + p ESI Q1MS [100.000-700.000]
201.91
244.88
305.00
227.87
369.88
386.26
284.42
166.84
m/z

57

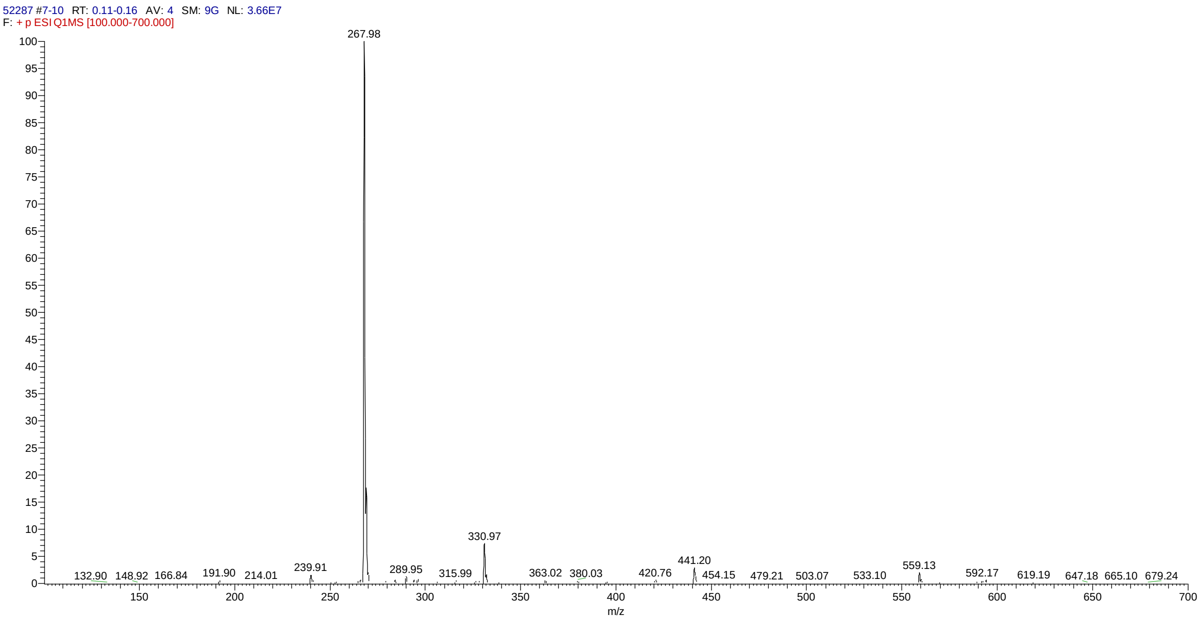
52287 #7-10 RT: 0.11-0.16 AV: 4 SM: 9G NL: 3.66E7
F: + p ESI Q1MS [100.000-700.000]
267.98
330.97
441.20
559.13
132.90 148.92 166.84 191.90 214.01 239.91 289.95 315.99 363.02 380.03 420.76 454.15 479.21 503.07 533.10 592.17 619.19 647.18 665.10 679.24
m/z

HPLC chromatograms

4a

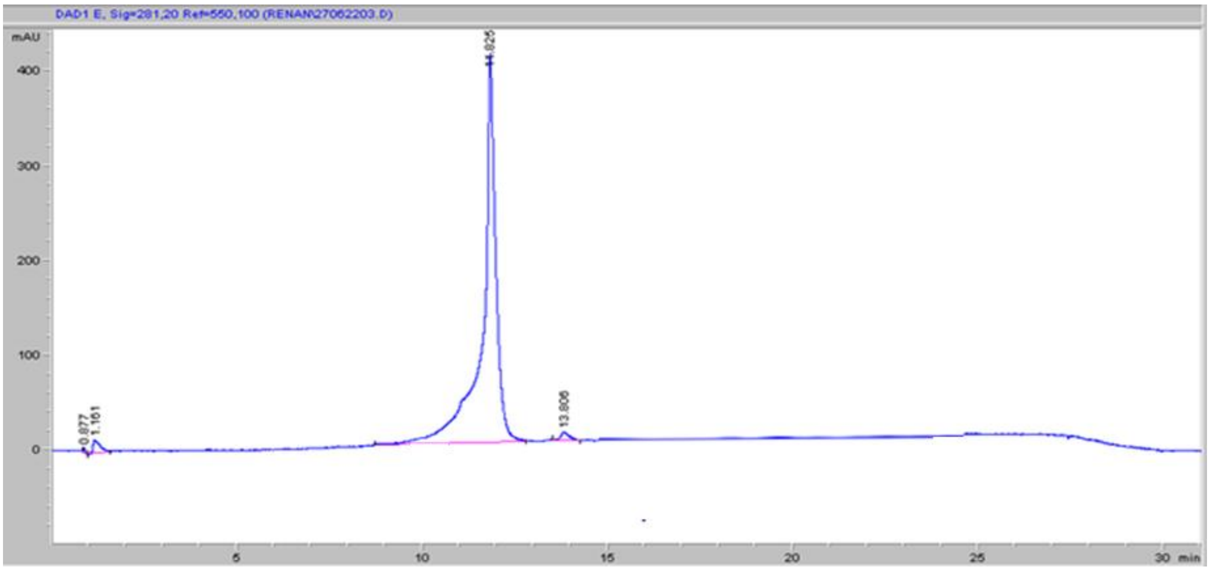

4b

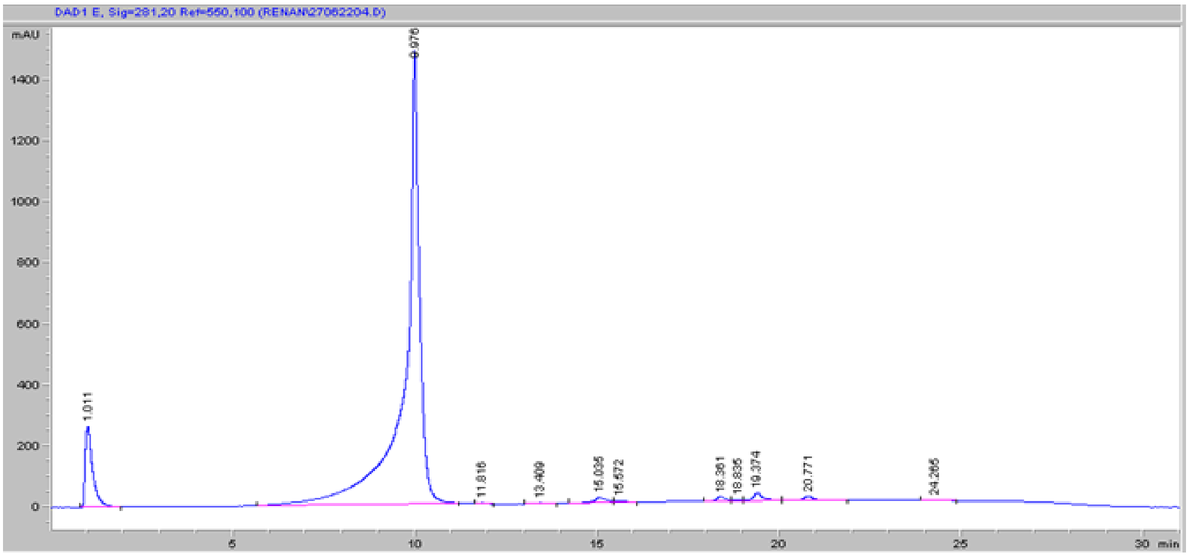

1.011 DMSO peak

6

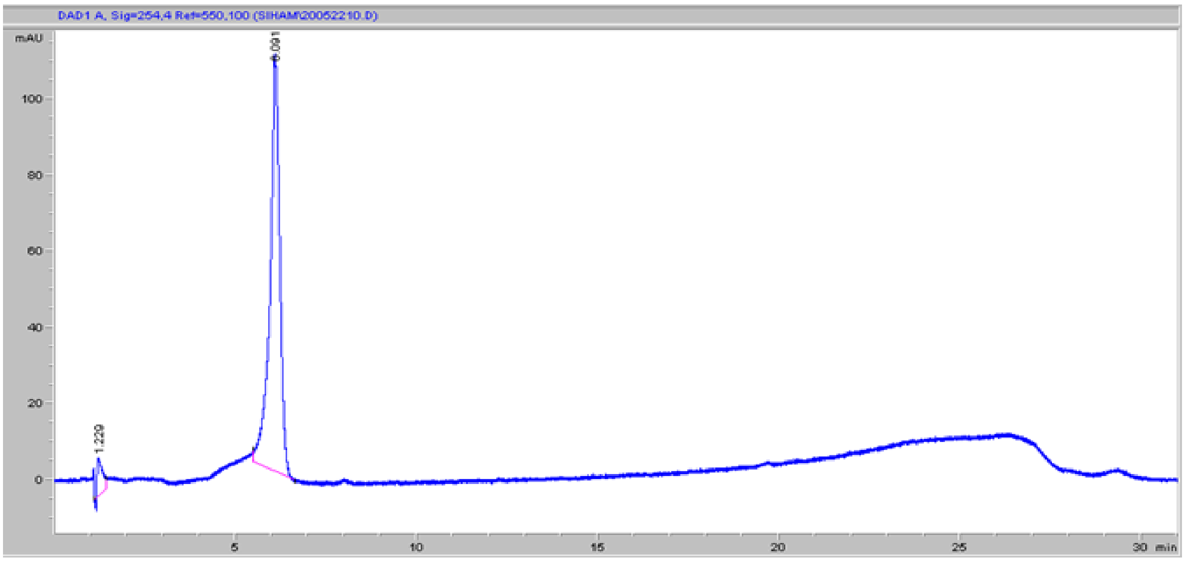
DAD1 A, Sig=254,4 Ref=550,100 (SIHAM\20052210.D)
mAU
100
80
60
40
20
0
1.229
6.091
5
10
15
20
25
30 min

7

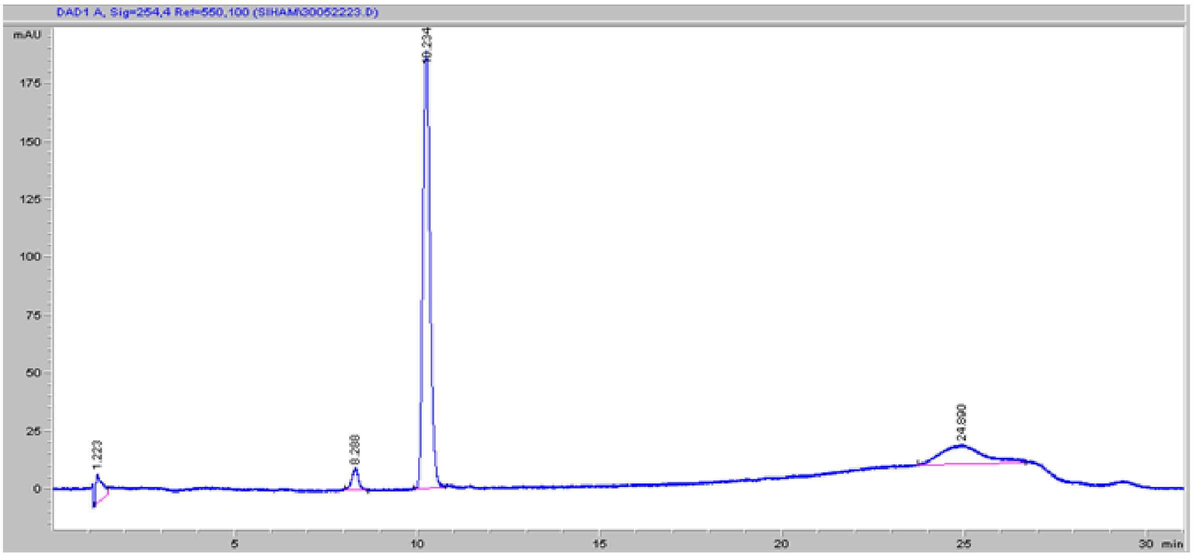
DAD1 A, Sig=254,4 Ref=550,100 (SIHAM\30052223.D)
mAU
175
150
125
100
75
50
25
0
1.223
8.288
10.234
24.890
5
10
15
20
25
30 min

16

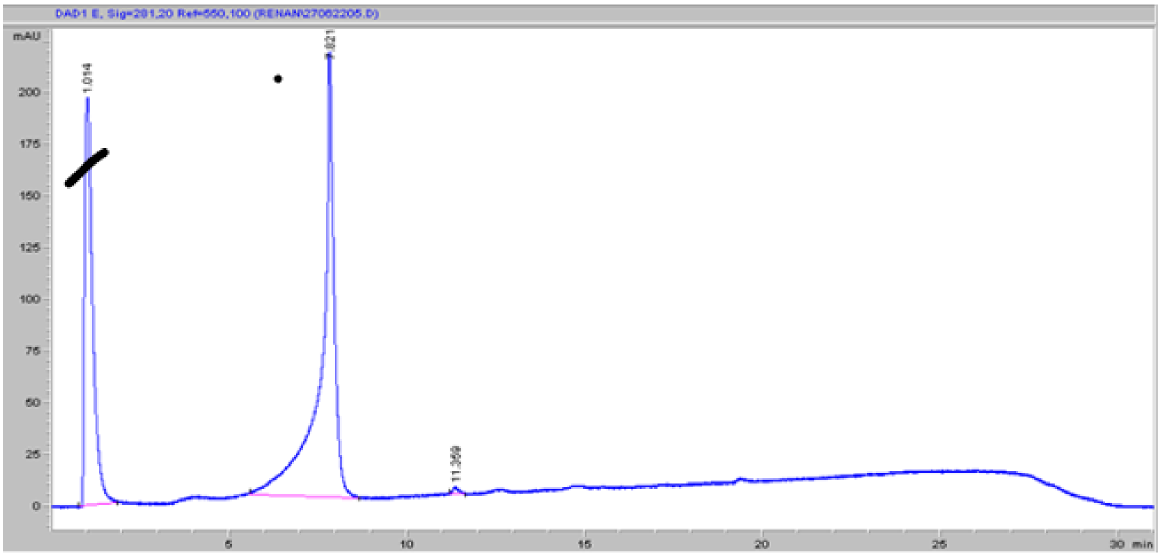

1.014 DMSO peak

17

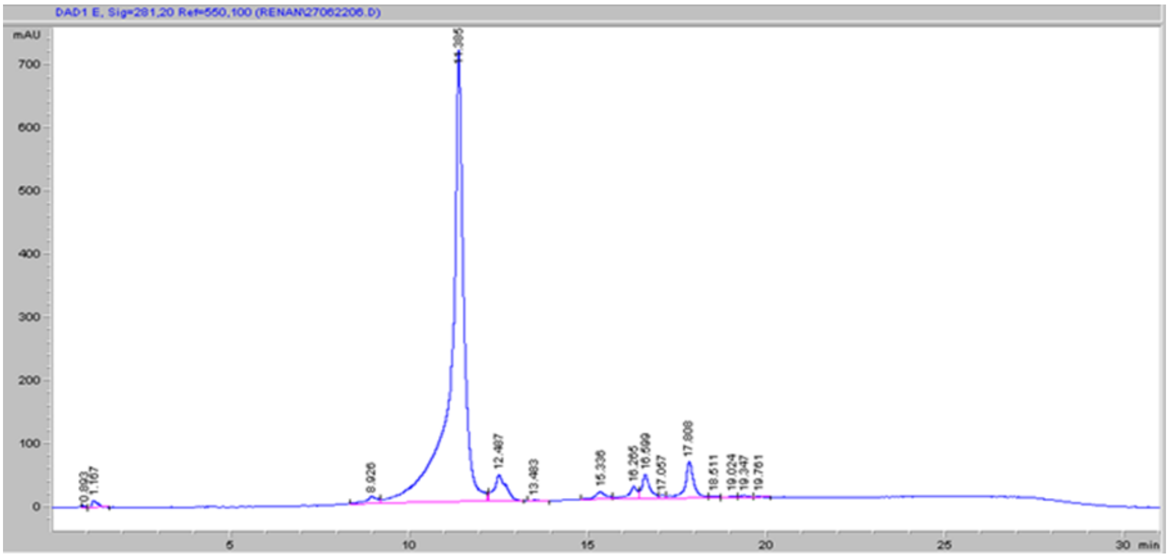

22

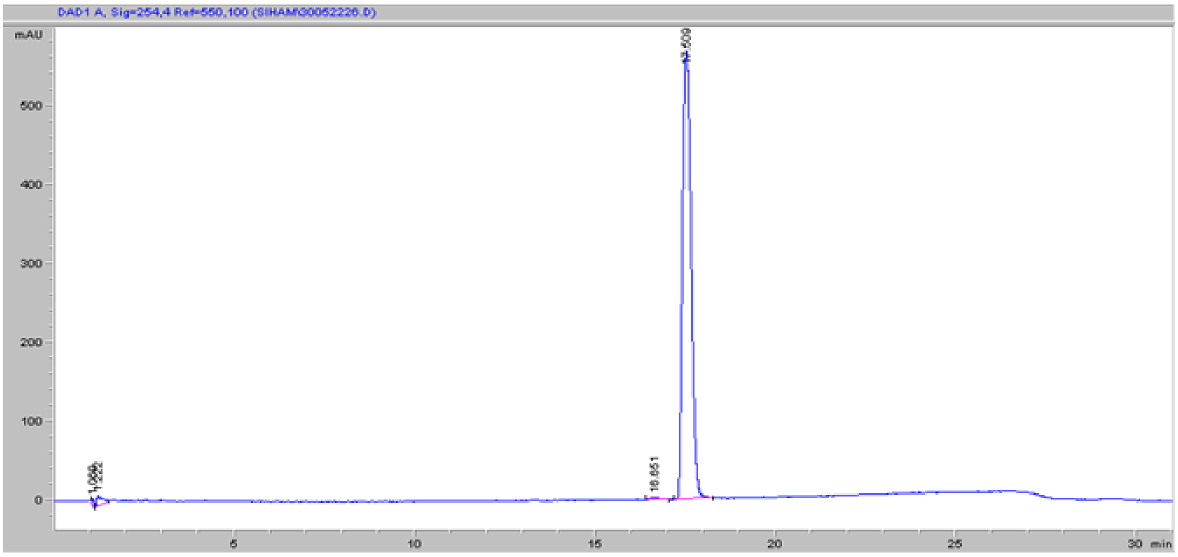
DAD1 A, Sig=254,4 Ref=550,100 (SIHAM\30052226.D)
mAU
500
400
300
200
100
0
16.651
17.509
5
10
15
20
25
30
min

27

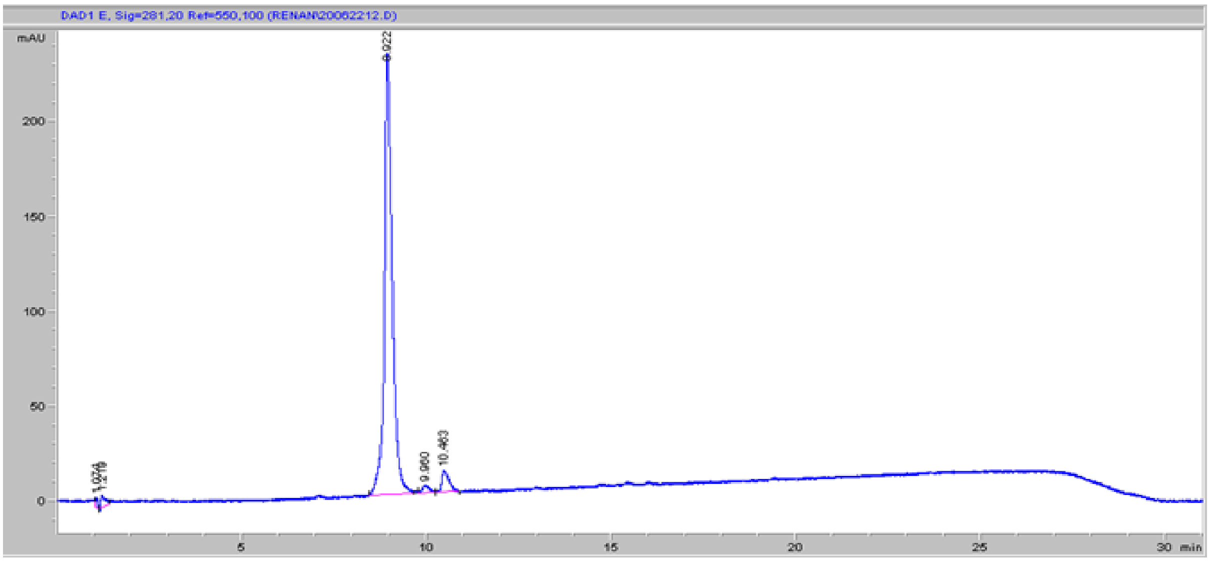
DAD1 E, Sig=281,20 Ref=550,100 (RENAN\20062212.D)
mAU
200
150
100
50
0
8.922
9.960
10.463
5
10
15
20
25
30
min

28

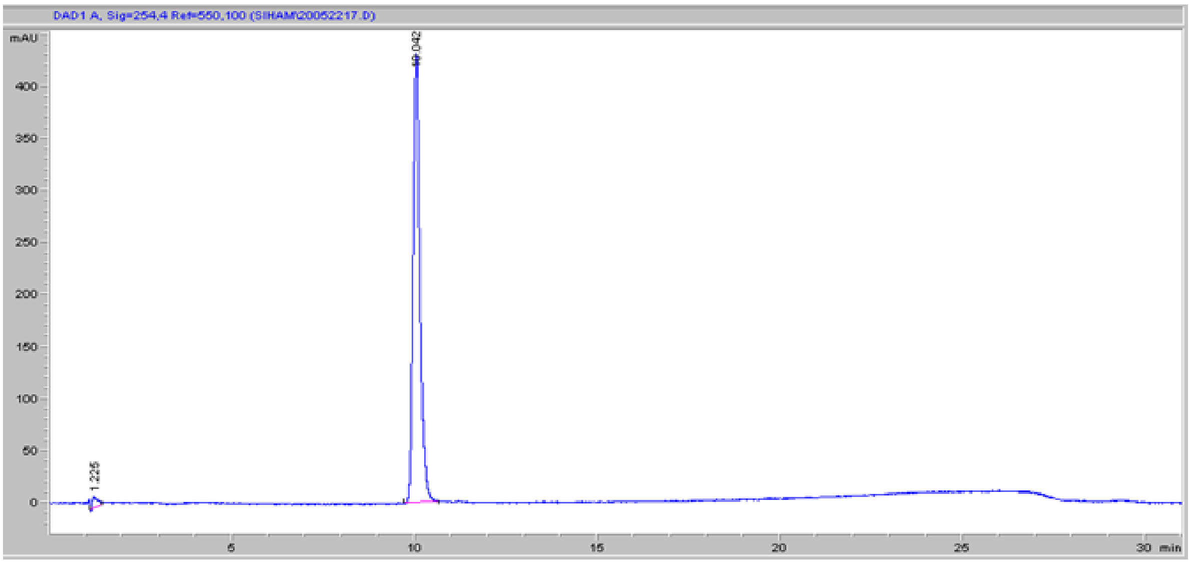
DAD1 A, Sig=254,4 Ref=550,100 (SIHAM\20052217.D)
mAU
400
350
300
250
200
150
100
50
0
1.225
5
10
15
20
25
30 min

29a

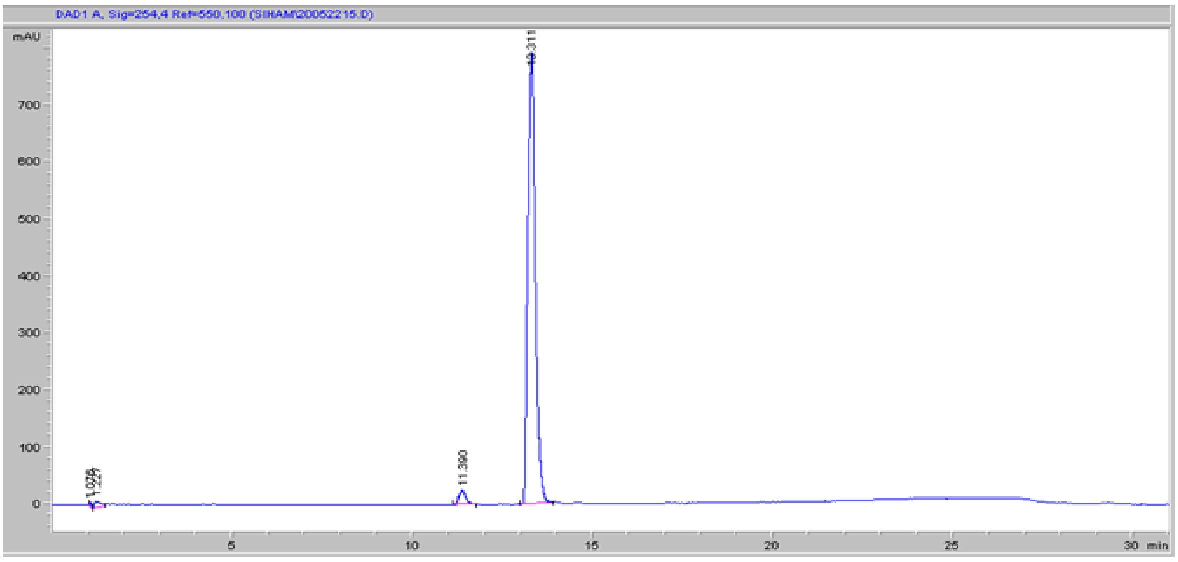
DAD1 A, Sig=254,4 Ref=550,100 (SIHAM\20052215.D)
mAU
700
600
500
400
300
200
100
0
11.390
5
10
15
20
25
30 min

29b

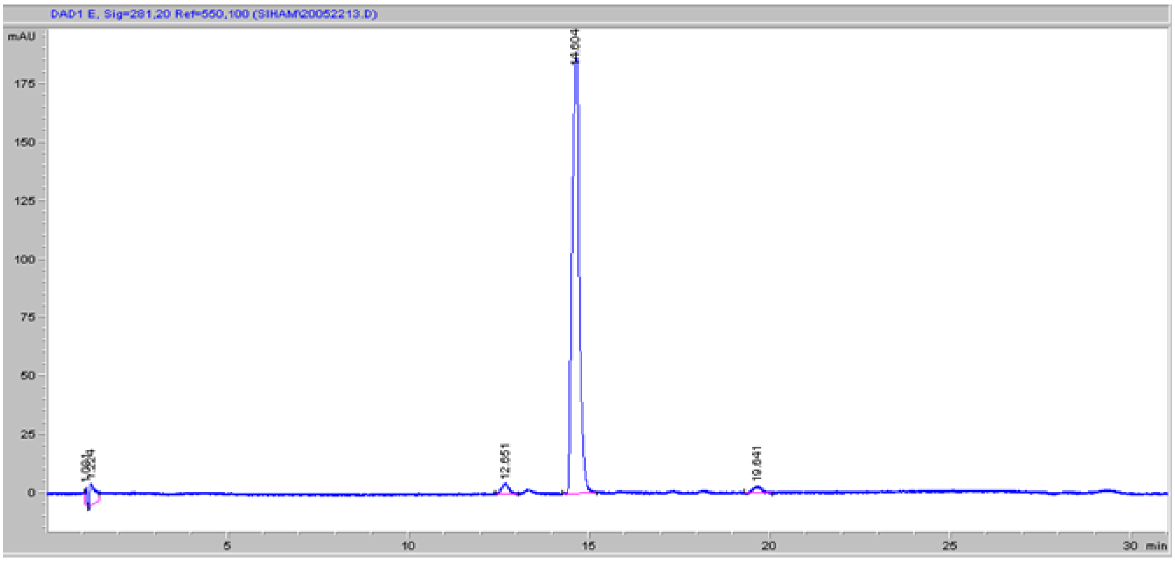
DAD1 E, Sig=281,20 Ref=550,100 (SIHAM\20052213.D)
mAU
175
150
125
100
75
50
25
0
5
10
15
20
25
30
min
12.651
14.604
19.641

29c

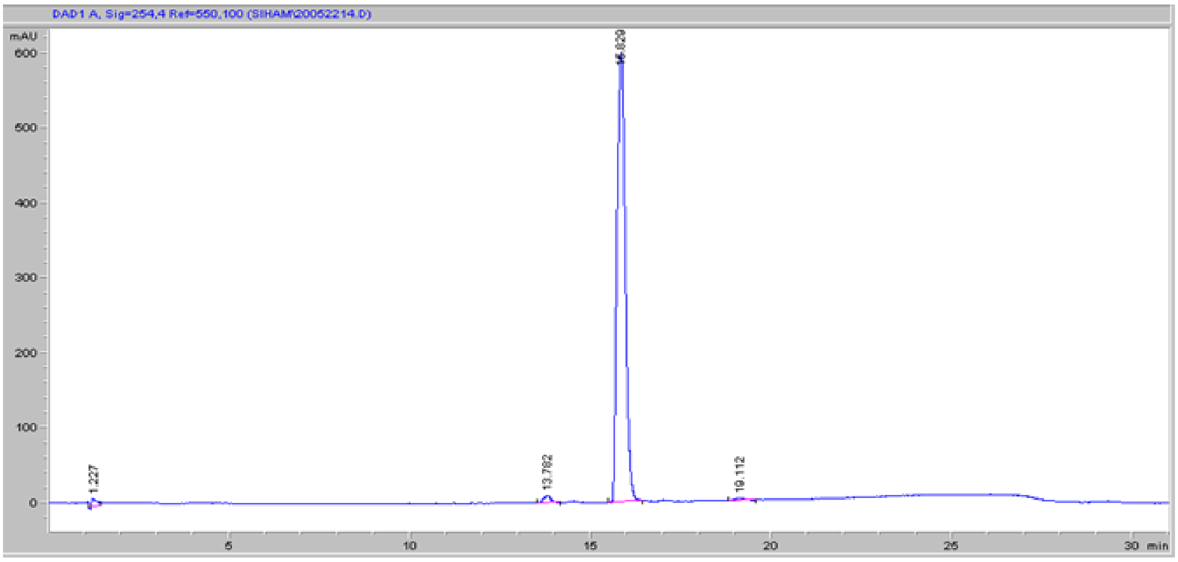
DAD1 A, Sig=254,4 Ref=550,100 (SIHAM\20052214.D)
mAU
600
500
400
300
200
100
0
5
10
15
20
25
30
min
1.227
13.782
19.112

30b

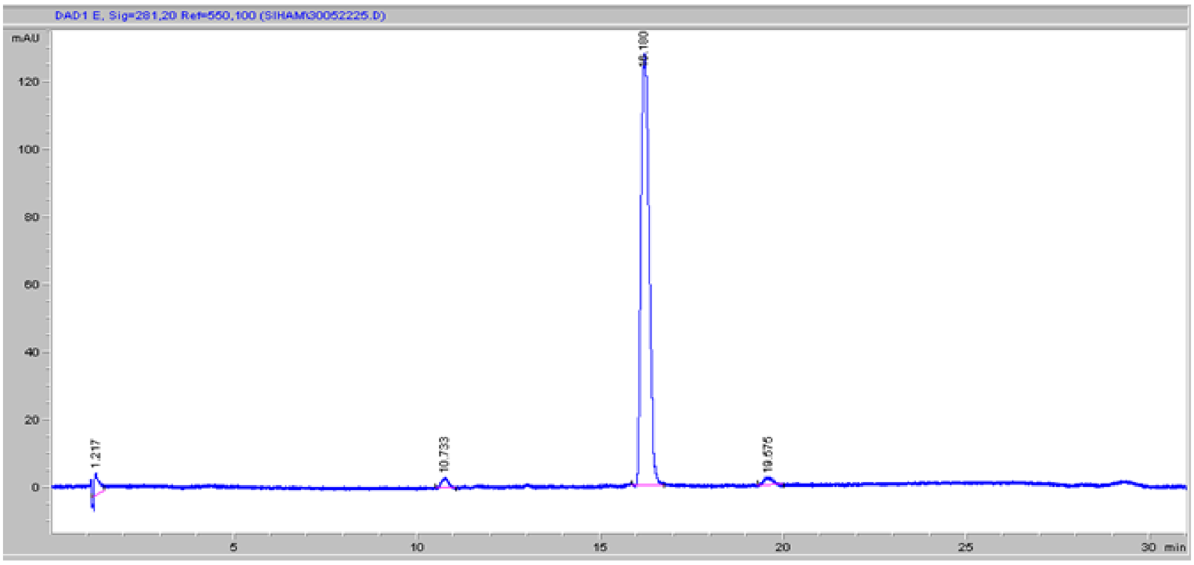
DAD1 E, Sig=281,20 Ref=550,100 (SIHAM\30052225.D)
mAU
120
100
80
60
40
20
0
1.217
10.733
19.575
5
10
15
20
25
30 min

35

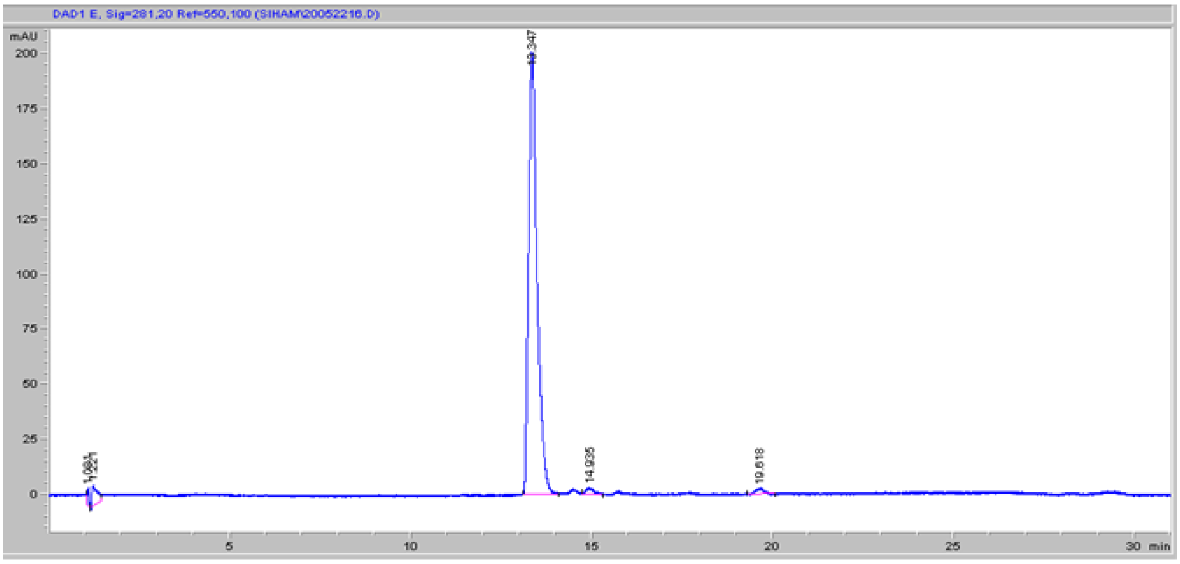
DAD1 E, Sig=281,20 Ref=550,100 (SIHAM\20052216.D)
mAU
200
175
150
125
100
75
50
25
0
14.935
19.618
5
10
15
20
25
30 min

42a

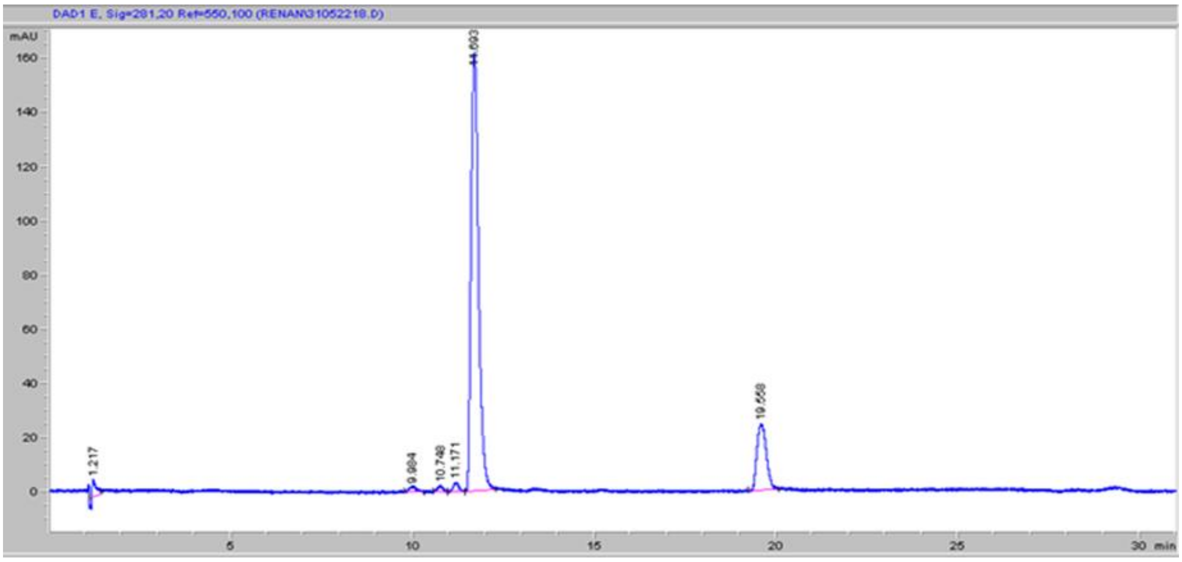
DAD1 E, Sig=281,20 Ref=550,100 (RENAN\31052218.D)
mAU
160
140
120
100
80
60
40
20
0
1.217
9.984
10.748
11.171
11.693
19.558
5
10
15
20
25
30
min

51

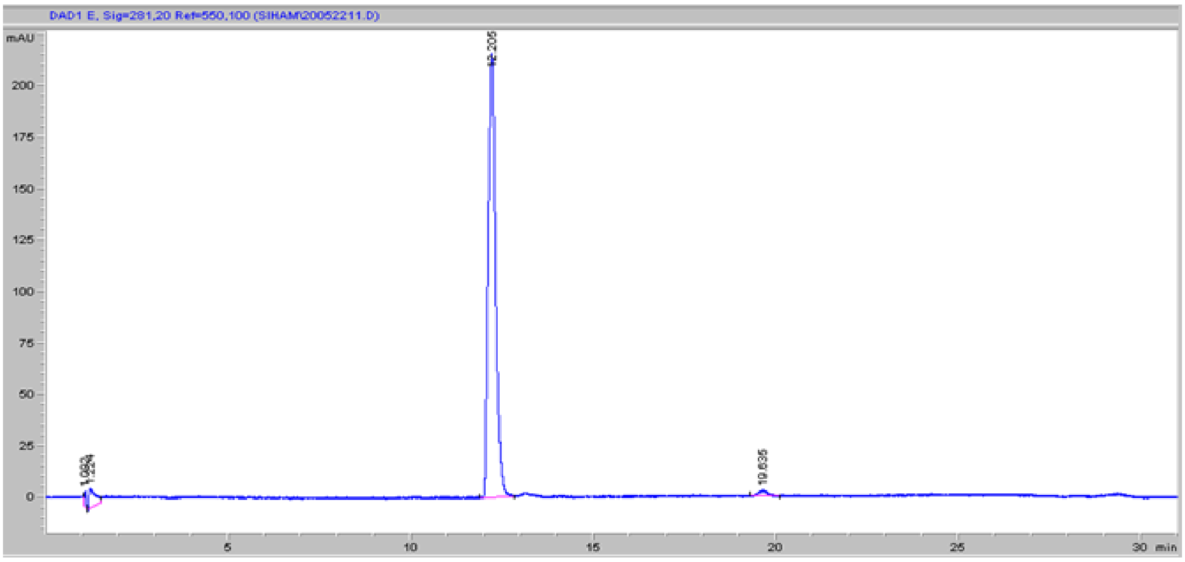
DAD1 E, Sig=281,20 Ref=550,100 (SIHAM\20052211.D)
mAU
200
175
150
125
100
75
50
25
0
12.205
19.635
5
10
15
20
25
30
min

54

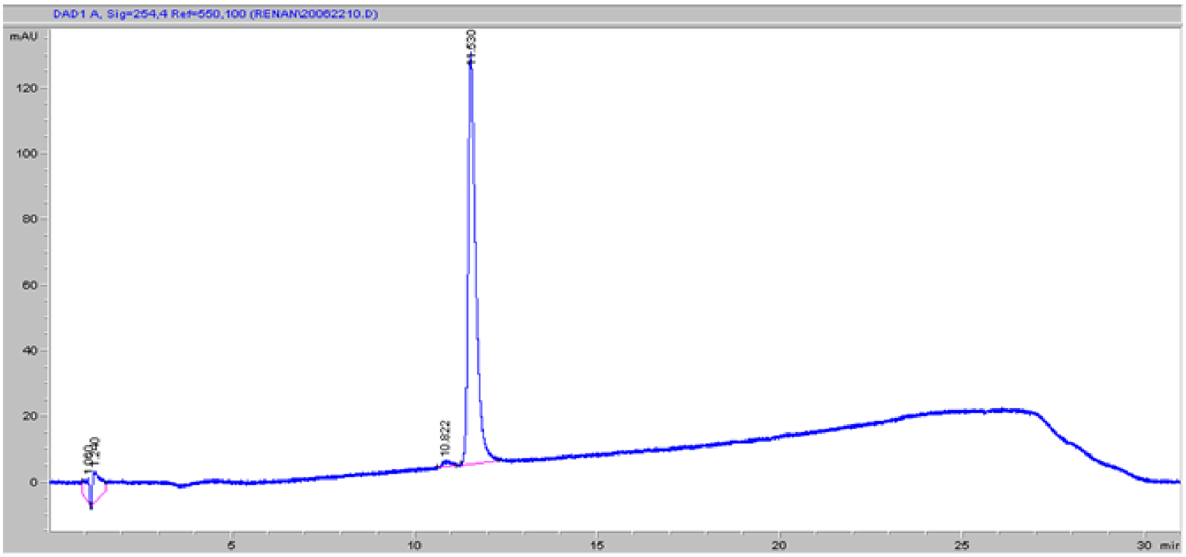
DAD1 A, Sig=254,4 Ref=550,100 (RENAN\20062210.D)
mAU
120
100
80
60
40
20
0
11.530
10.822
5
10
15
20
25
30 min

55

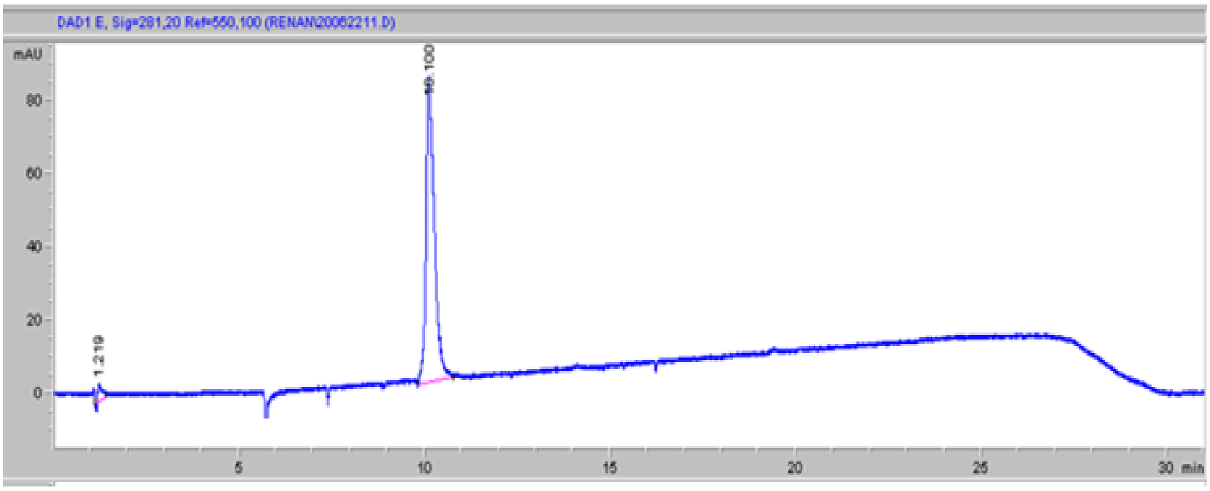
DAD1 E, Sig=281,20 Ref=550,100 (RENAN\20062211.D)
mAU
80
60
40
20
0
10.100
1.219
5
10
15
20
25
30 min

57

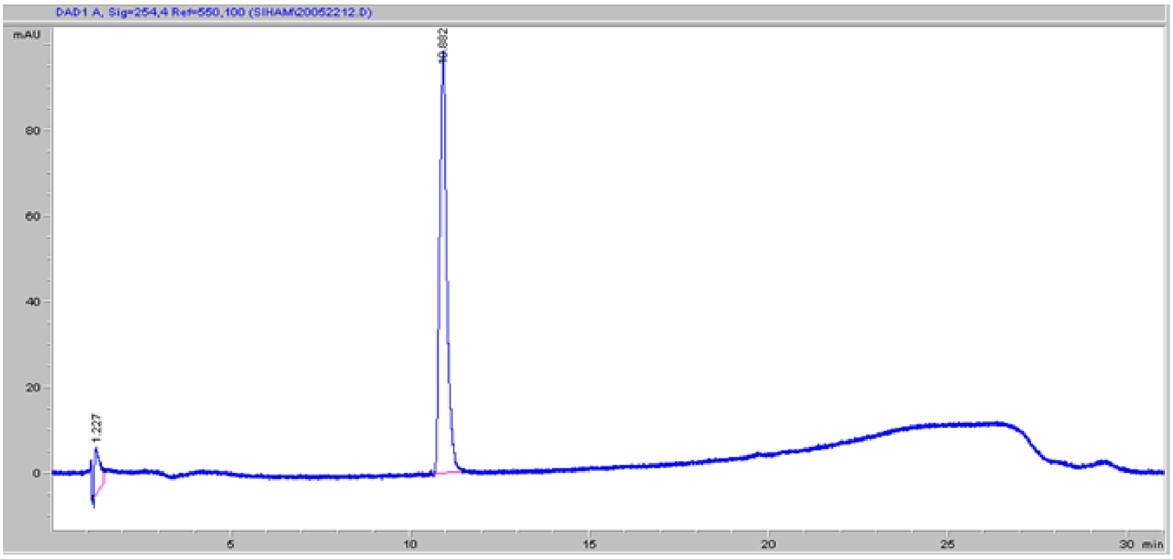

DAD1 A, Sig=254,4 Ref=550,100 (SIHAM\20052212.D)
mAU
80
60
40
20
0
1.227
10.882
5
10
15
20
25
30 min

Table S1. 50 best compounds generated with scaffold hopping replacement.

Rank	Structure	Score	Field Score	Shape Score
1	CN1N=C(C)C(SCC(N2CCCCC2)=O)CC1=O	0.881	0.837	0.925
2	FC(F)(F)c1cccc(SCC(=O)N2CCCCC2)c1	0.869	0.807	0.93
3	Clc1cccc(SCC(=O)N2CCCCC2)c1	0.861	0.792	0.931
4	Clc1ccc(S(=O)(=O)CC(=O)N2CCCCC2)cc1C(F)(F)F	0.853	0.813	0.893
5	Fc1ccc(N)c(SCC(=O)N2CCCCC2)c1	0.85	0.81	0.889
6	Fc1cccc(SCC(=O)N2CCCCC2)c1	0.849	0.787	0.912
7	Fc1cc(N)cc(SCC(=O)N2CCCCC2)c1	0.848	0.775	0.922
8	FC(F)(F)c1ccnc(SCC(=O)N2CCCCC2)n1	0.847	0.82	0.875
9	FC(F)(F)c1ccnc(S(=O)(=O)CC(=O)N2CCCCC2)c1	0.846	0.782	0.909
11	O=C(N1CCCCC1)CC/C=C/CCCC	0.844	0.811	0.877
10	Brc1ccc(N)c(S(=O)(=O)CC(=O)N2CCCCC2)c1	0.844	0.806	0.882
12	O=S(=O)(CC(=O)N1CCCCC1)c2cccc3c2ncs3	0.836	0.773	0.899
13	FC(F)(F)c1ccc(c(S(=O)(=O)CC(=O)N2CCCCC2)c1)C	0.832	0.785	0.879
14	Clc1ccc(N)c(S(=O)(=O)CC(=O)N2CCCCC2)c1	0.83	0.779	0.88
15	O=S(=O)(CC(=O)N1CCCCC1)c2cccc(N(C)C)c2	0.829	0.766	0.892
17	O=S(=O)(c1cc(C(C)(C)C)ccc1C)CC(=O)N2CCCCC2	0.826	0.783	0.869
18	Fc1ccc(SCC(=O)N2CCCCC2)cc1F	0.826	0.761	0.89
16	O=C(N1CCCCC1)COc2cccc(N(C)C)c2	0.826	0.758	0.894
19	O=C(N1CCCCC1)CSc2cccc(N)c2	0.826	0.755	0.896
20	FC(F)(F)c1ccc(N)c(C(=O)CC(=O)N2CCCCC2)c1	0.825	0.74	0.911
21	OCc1cccc(SCC(=O)N2CCCCC2)c1	0.825	0.71	0.94
23	O=C(N1CCCCC1)CC(c2nccc(n2)C)C	0.823	0.763	0.883
22	FC(F)(F)c1cccc(C(O)CC(=O)N2CCCCC2)c1	0.823	0.76	0.886
25	O=C(N1CCCCC1)CC(=O)c2cc(N)cc(OC)c2	0.821	0.724	0.918
24	Fc1cc(cc(CCC(=O)N2CCCCC2)c1)C(F)(F)F	0.821	0.721	0.921
26	O=C(N1CCCCC1)CSc2cccc(c2N)C	0.82	0.764	0.875
27	Clc1cc(nc(SCC(=O)N2CCCCC2)n1)N	0.818	0.789	0.847
28	FC(F)(F)c1cc(nc(SCC(=O)N2CCCCC2)n1)C	0.817	0.726	0.907
29	FC(F)(F)c1cc(C(=O)CC(=O)N2CCCCC2)ccc1C	0.816	0.749	0.884
30	O=S(=O)(N1CCCC1)NCCC(=O)N2CCCCC2	0.815	0.748	0.883
33	O=S(=O)(CC(=O)N1CCCCC1)c2cccc(OC)c2	0.815	0.742	0.888
31	O=C(N1CCCCC1)CC(=O)c2cccc(OC)n2	0.815	0.736	0.895
32	FC(F)(F)c1ccc(F)c(C(=O)CC(=O)N2CCCCC2)c1	0.815	0.733	0.898
34	O=C(N1CCCCC1)CCCc2c(oc(n2)C)C	0.814	0.74	0.888
36	Clc1cccc(S(=O)(=O)CC(=O)N2CCCCC2)c1	0.813	0.771	0.855
35	O=C(N1CCCCC1)CC[NH2+]CCCSC	0.813	0.761	0.866
37	O=C(N1CCCCC1)CCCNc2cccc[nH+]2	0.813	0.747	0.878
38	Fc1cc(N)cc(CCC(=O)N2CCCCC2)c1	0.812	0.727	0.897
39	O=C(N1CCCCC1)CC(=O)c2cncc(OC)n2	0.812	0.721	0.902
43	O=C(N1CCCCC1)CSc2nc(OC)cc(OC)n2	0.811	0.745	0.876
42	O=C(N1CCCCC1)CSc2ccc3CCCc3c2	0.811	0.74	0.882
40	O=C(N1CCCCC1)CCSc2nccc(n2)C	0.811	0.733	0.889
41	O=C(N1CCCCC1)CCCc2cccc(n2)C	0.811	0.732	0.889
44	O=C(N1CCCCC1)CCCc2nc(cc(n2)C)C	0.809	0.737	0.88

45	O=C(N1CCCCC1)COc2cccc(c2)C=O	0.809	0.728	0.889
46	FC(F)(F)c1cc(S(=O)(=O)CC(=O)N2CCCCC2)ccc1C	0.808	0.749	0.866
48	I/C=C/CCC(=O)N1CCCCC1	0.807	0.775	0.839
47	O=C(N1CCCCC1)CSc2nsc(n2)N	0.807	0.766	0.848
50	O=C(N1CCCCC1)CSc2cnccc2	0.807	0.736	0.877
